# β-Galactosidase- and Photo-Activatable Fluorescent Probes for Protein Labeling and Super-Resolution STED Microscopy in Living Cells

**DOI:** 10.3390/molecules29153596

**Published:** 2024-07-30

**Authors:** Taukeer A. Khan, Stefan Stoldt, Mariano L. Bossi, Vladimir N. Belov, Stefan W. Hell

**Affiliations:** 1 Department of NanoBiophotonics, Max Planck Institute for Multidisciplinary Sciences (MPI-NAT), Am Fassberg 11, 37077 Göttingen, Germanyvladimir.belov@mpinat.mpg.de (V.N.B.);; 2 Department of Optical Nanoscopy, Max Planck Institute for Medical Research (MPI-MR), Jahnstrasse 29, 69120 Heidelberg, Germany; mariano.bossi@mr.mpg.de

**Keywords:** fluorescence, photoactivation, enzyme activation, STED microscopy, organic synthesis, fluorescent dyes

## Abstract

We report on the synthesis of two fluorescent probes which can be activated by β-Galactosidase (β-Gal) enzymes and/or light. The probes contained 2-nitro-4-oxybenzyl and 3-nitro-4-oxybenzyl fragments, with β-Gal residues linked to C-4. We performed the enzymatic and photoactivation of the probes in a cuvette and compared them, prior to the labeling of *Vimentin–Halo* fusion protein in live cells with overexpressed β-galactosidase. The dye fluorescence afforded the observation of enzyme activity by means of confocal and super-resolution optical microscopy based on stimulated emission depletion (STED). The tracing of enzymatic activity with the retention of activated fluorescent products inside cells was combined with super-resolution imaging as a tool for use in biomedicine and life science.

## 1. Introduction

Owing to high detection sensitivity, spatiotemporal resolution, and non-invasive real-time read-out, fluorescence techniques provide a powerful tool with which to visualize the dynamic processes in living specimens [1,2,3]. Many human diseases, especially cancer, have proven to be strongly associated with enzyme activities. Enzymes play a fundamental and often crucial role in the clinical diagnosis and treatment of diseases [3,4,5,6,7,8,9], and their activities are measured by means of fluorescence read-out [10,11,12]. Responsive fluorescent probes stay in an *off* state until they are turned *on* by interacting with a specific biological target (e.g., enzyme). Nowadays, STED microscopy has become a routine super-resolution technique, but it has not yet become widely used in experiments involving enzymatically activatable probes.

β-Galactosidase (β-Gal), a typical glycoside hydrolase enzyme, is involved in the catalytic hydrolysis of glycosidic bonds; it converts lactose into galactose [6,13]. Previous studies have shown that levels of β-galactosidase are upregulated in cancer, particularly ovarian cancer [5,13,14]. In addition, the senescence-associated β-galactosidase (SA-β-Gal) expressed in lysosomes is commonly used as a biomarker that identifies senescent cells and aging tissues [15,16], and these cells exhibit a higher level of β-Gal activity compared to normal human cells [17]. The development of accurate, rapid, and efficient methods for visualizing, monitoring, and tracking the activity of β-Gal is important for early cancer diagnosis. Various fluorescent probes have been developed to assess activity and visualize β-Gal distribution in cancer and senescent cells [5,6,13,18,19,20,21].

**Scheme 1:**
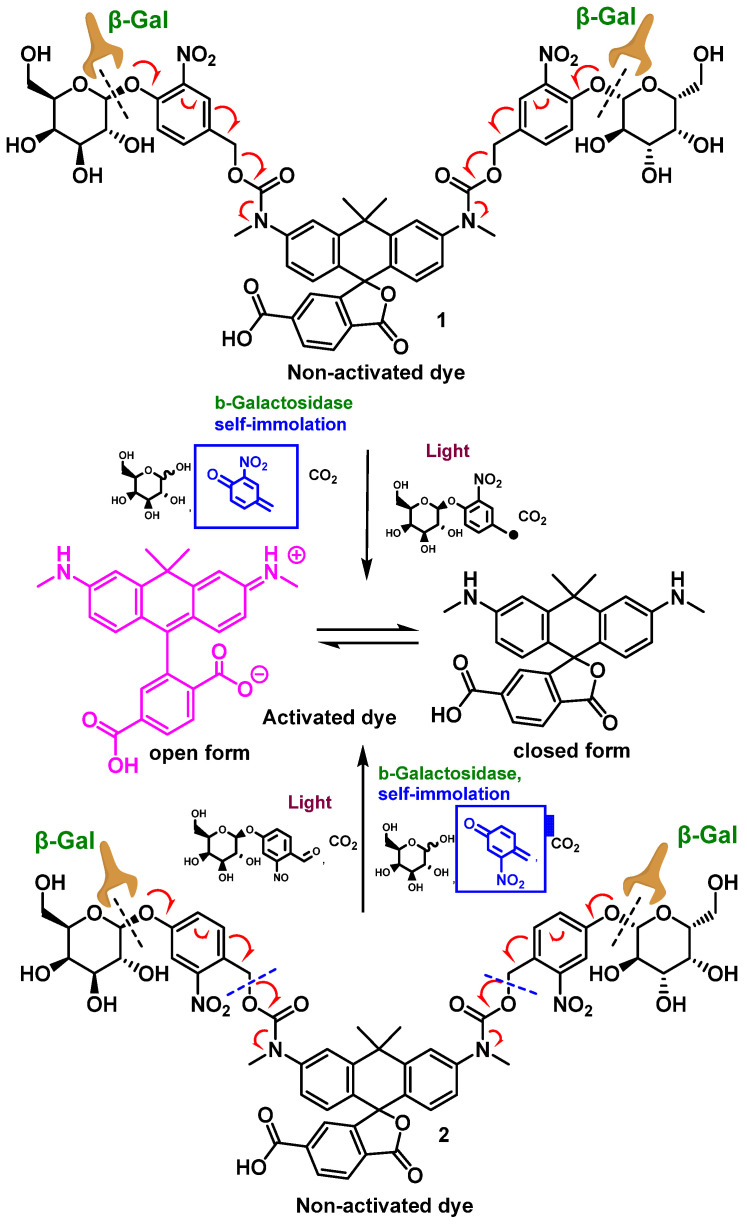
Enzyme/light-activatable probes (**1**, **2**) for sensing β-Gal activity and dual response (enzyme + light). The self-detachable 3- or 2-nitro-4-oxybenzyl groups are shown in blue (enzymatic activation).

One of the drawbacks of the reported probes is their poor retention of activated fluorescent products within cells. To prevent diffusion out of cells, biorthogonal reaction-based strategies, like click reaction and quinone methide chemistry, were applied [18,22]. The covalent attachment of fluorophores to an enzyme as a tool with which to retain dyes within cells possibly causes enzyme dysfunction and leads to its inactivation [23,24]. Some existing probes are membrane-impermeable and show low responses [5]. Hence, there is a need for new fluorogenic probes to provide the on-site and long-term visualization of β-Gal activity in living cells and retain fluorescent products inside cells. Addressing this challenge, we prepared two structurally related fluorescent probes used to target β-Gal in living cells. For that, we selected a bright, photostable and bio-compatible fluorophore (Scheme 1) and masked (“caged”) its fluorescence emission by the addition of two enzymatically cleavable groups connected with photocleavable units. Both probes are dual-responsive and can be activated using enzymes and/or light. We found that a slight change in the structure of the caging group (at the position of the nitro group in the phenyl ring) resulted in sharp variation in the activation profile. The first probe (Scheme 1) is more responsive to β-Gal than the second one, while the second one is more responsive to violet and UV light (365–405 nm) than the first one. The dual response (enzyme + light) may be used in experiments when the enzymatic activity is low, and the probe localization, distribution or dynamics may be revealed or tracked by “light injections” (photolysis).

## 2. Results and Discussion

### 2.1. Design and Synthesis

As a dye, we selected carborhodamine 580CP, which emits at 607 nm (emission maxima). The dye 580CP is bright and photostable with a very good imaging performance in conventional (widefield, confocal) and super-resolution STED microscopy [25]. In our previous reports, we incorporated 580CP into the structure of actin and nucleopore probes for STED and MINFLUX nanoscopy [26]. Apart from the dye, two galactose residues were incorporated as enzyme recognition units and connected with two photocleavable caging groups to fully eliminate the emission of the dye by keeping it in a spirolactone (masked) form (Scheme 1).

The activatable probes shown in Scheme 1 contain two enzymatically cleavable galactose units, bound with two “self-immolative” [27] groups (3- or 2-nitrobenzyl) through glycosidic bonds (at C-4 in benzyl). The carbamate groups are attached to methylene carbons and keep the dye in a non-fluorescent “closed-ring” form, which can be subsequently activated upon detaching the sugar units using a β-Gal enzyme or photolysis. The structure of the cleavable group in compound **1** [(3-nitro-4-oxybenzyl)oxycarbonyl] is quite similar to the structure of the photocleavable (2-nitro-4-oxybenzyl)oxycarbonyl group in compound **2** [26,28,29]. However, the cleavage mechanisms for enzyme reactions and photoreactions are different. The β-Gal-responsive unit is attached to C-4 in benzyl using an oxygen atom. Upon the detachment of the sugar units, caused by the action of β-Gal, the caging groups are cleaved via “self-immolation”. The functionality of *2*-nitrophenyl-β-D-galactopyranoside is due to its high affinity for β-Gal enzyme [30]. 

**Scheme 2:**
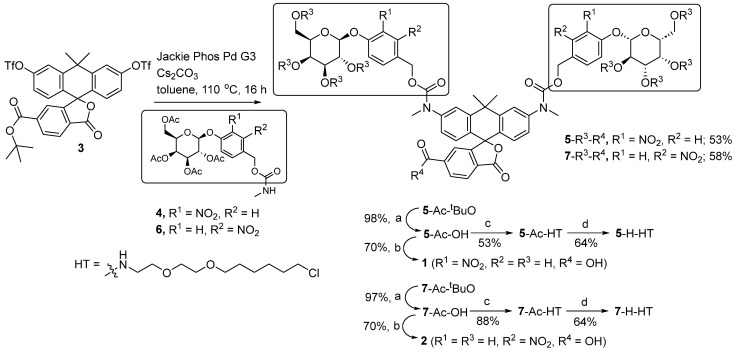
The synthesis of carboxylic acids **1** and **2** with the various positions of the nitro group (R^1^, R^2^) in the enzymatically and/or photocleavable caging groups (shown in frames), as well as their analogs **5**-H-HT and **7**-H-HT, decorated with a HaloTag^TM^ ligand. Reagents and conditions: (a) CF_3_CO_2_H, CH_2_Cl_2_, rt, 16 h; (b) LiOH, aq. MeOH, rt, 1.5 h; (c) PyBOP, DIPEA, DMF, H_2_N(CH_2_CH_2_O)_2_(CH_2_)_6_Cl, rt, 2 h; (d) LiOH, H_2_O, MeOH, rt, 6 h.

Activatable probes (compounds **1** and **2** in Scheme 1) contain a free COOH group for attaching a HaloTag ligand (in our case; see Scheme 2) or preparing bioconjugates. We wanted to create cell-permeable probes. The spirolactone form of the fluorophore has a zero net charge (in conjugates when carboxylate is derivatized). The absence of negative charge favors cell permeability. On the other hand, the molecular mass and molecular size could not be reduced due to the presence of fluorophore and two caging groups. These factors and the polycyclic structure reduce solubility in water and cell permeability. Fortunately, the use of two sugar residues with eight hydroxyl groups would increase the probe polarity and hydrophilic properties (compared to analogues with unsubstituted or methoxy-substituted (2-nitrobenzyl)oxycarbonyl groups) [26,29]. Upon decoration with a HaloTag ligand (Scheme 2), the probe is expected to be retained within cells containing HaloTag constructs (in our case, *Vimentin–Halo* fusion). Thus, intracellular capture is considered to be important in observing the activity of β-Gal in living cells for longer times without the back-diffusion of the probe through the cell membrane. Dye fluorescence is expected enable the assessment and visualization of enzyme activity (and its evolution in time) by means of confocal and super-resolution STED microscopy.

In addition to probe **1** (Scheme 1), we planned to prepare its analog (compound **2**), which is also dual-responsive: it can be activated by β-Gal or light (Scheme 1). The dual-responsive probes may be used in experiments when the enzymatic activity is low. In these cases, the maximal level of observable emission can be assessed by photolysis. By changing the position of the nitro group in the 3-nitro-4-oxybenzyl residue in compound **1**, we prepared the 2-nitrobenzyl analog (compound **2**), which undergoes photolysis much more readily than compound **1**. 

For probe **2**, enzyme activation proceeds similarly to that in probe **1**, while UV light irradiation is supposed to involve proton transfer from CH_2_ to NO_2_ and the cleavage of C-O bond between benzyl and the carbamate residue, liberating the substituted nitroso benzaldehyde, CO_2_, and the activated dye. This is shown in Scheme 1 [31]. The pendant COOH groups in probes **1**, **2** can be used to attach any functionality (Scheme 2) required for the labeling of biomacromolecules or other objects of interest.

### 2.2. Synthesis of Probes 1 and 2

The syntheses of carboxylic acids **1** and **2** with the various positions of the nitro group (R^1^, R^2^) in the enzymatically and/or photocleavable caging groups (shown in frames in Scheme 2), as well as their analogs **5**-H-HT and **7**-H-HT—decorated with a HaloTag^TM^ ligand—are shown in Scheme 2. Compounds **4** and **6—**sugar-substituted (at C-4) 3- and 2-nitrobenzyl carbamates [32]**—**were prepared and combined in a Buchwald-Hartwig amidation reaction (catalyzed by Pd-JackiePhos) [33] with compound **3** [6′-(*tert*-butoxycarbonyl) carbofluorescein bis-triflate] [26] to produce esters **5**-Ac-*^t^*BuO and **7**-Ac-*^t^*BuO, the desired caged 580CP dye derivatives with protecting groups. The deprotection of 6′-*tert*-butyl carboxylates gave masked dyes **5**-Ac-OH and **7**-Ac-OH. The sugar moieties in these dye-carboxylic acid derivatives were still acetylated and could be deprotected via mild basic hydrolysis (or acetyl transfer in methanol) to obtain the target carboxylic acids **1** and **2** (Scheme 2). 

Finally, we prepared activatable probes with a group (ligand), enabling the selective and irreversible (covalent) labeling of HaloTag [34] fusion proteins in living cells. For that, compounds **5**-Ac-OH and **7**-Ac-OH were reacted with the HaloTag amine (O2) to obtain amides **5**-Ac-HT and **7**-Ac-HT, and after the cleavage of acetyl groups from sugar moieties, the probes **5**-H-HT and **7**-H-HT were obtained (Scheme 2). The detailed synthetic procedures are given in the Supporting Information (Schemes S3 and S6). As a target protein in living cells, we used Vimentin–HaloTag fusion, which provides fast reaction [35,36]. There are several stable cell lines that have this protein tag, which is fused with Vimentin, proteins in nuclear pore complexes, and other targets [37]. The structures of all compounds were confirmed via NMR spectra. The copies of ^1^H- and ^13^C-NMR spectra are given in Supporting Information. The constitution and purity degrees of probes **1** and **2** were determined via HR-MS and HPLC analyses, respectively. The transformations (enzymatic, (photo)chemical) of probes **1** and **2** were monitored by UV-Vis and fluorescence spectroscopy, as well as HPLC and LCMS analysis.

### 2.3. Activation and Optical Characterization

**Figure 1:**
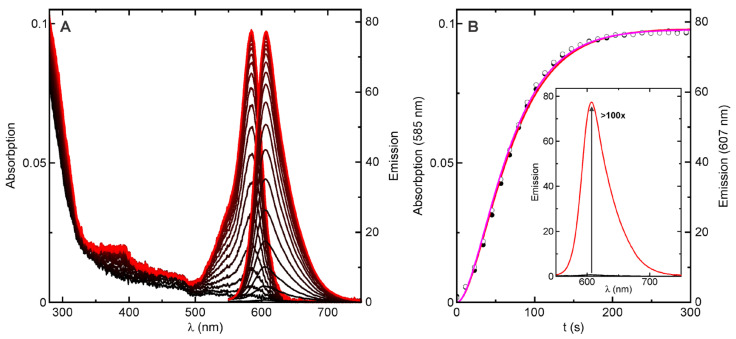
Activation of carboxylic acid **1.** (**A**) Absorption (**left**) and emission (**right**) increases in a 1.3 µM solution of compound **1** in aqueous phosphate buffer (pH = 7) at 37 °C, recorded upon stepwise addition of β-galactosidase (3 units/mL). (**B**) Absorption (filled circles, left axis) and emission (hollow circles, right axis) increases at the corresponding maxima, fitted to a double exponential function (red and purple lines) and monitored in real time (0–5 min). The inset shows the enhancement of the emission signal (black baseline: emission intensity before the enzyme addition; red line: emission intensity upon saturation observed in the enzymatic cleavage of caging groups).

The activation of the probes **1** and **2** and their response towards β-Gal was tested using absorption and fluorescence spectroscopy. First, we investigated the enzymatic activation of carboxylic acid **1** by β-Gal in a cuvette experiment. For that, we confirmed the enzyme activity in a control run with *o*-nitrophenyl-β-D-galactopyranoside (ONPG) (see Scheme S1 and Figure S1 in Supporting Information). With low concentrations of a model substrate (ONPG), the reaction kinetics can be approximated with a simple monoexponential function (see Figure S1). Then, we determined the response of carboxylic acid **1** (with NO_2_ group in the *ortho*-position to galactose residue) to β-Gal enzyme by monitoring the absorption and fluorescence spectra in an aqueous phosphate buffer (100 mM, pH = 7) at 37 °C (Figure 1). A remarkable increase in the emission intensity was observed, and thus the workability of this assay was demonstrated. 

The sigmoidal shape of the absorption and emission responses was consistent with two consecutive steps involving a non-emissive and colorless intermediate. Unsurprisingly, only the final product absorbed and emitted light in the visible range. For compound **1**, a reasonable fit was obtained for a double exponential function with the same kinetic constant for both consecutive steps. Similar values of the pseudo-first-order rates were observed for ONPG (0.046 s^−1^) and compound **1** (0.031 s^−1^) under identical conditions (Figure S1). Thus, the enzymatic cleavage of each of the two glycosidic bonds proceeds independently, within the uncertainty level, and at the studied range of substrate and enzyme concentrations (Figure S2). For the reaction of compound **1**, we found that the absorption changes at 450 nm, dominated by the *o*-nitroso benzaldehyde byproduct, follow a similar trend to that of the model substrate ONPG (i.e., a pseudo-monoexponential behavior). This enabled the calculation of initial rates and the estimation of the Michaelis–Menten constant K_M_ (Figure S3). At 37 °C and pH = 7, we obtained a value of 0.115 mM for compound **1**, which was about half of the value found for the reference ONPG standard (0.240 mM). Overall, compound **1** possesses an efficient double enzymatic “cage” that minimizes the background emissions and produces an increase in the fluorescence signal of at least 2 orders of magnitude upon activation (Figure 1B).

### 2.4. Comparison of Enzymatic activation and Photoactivation of Probes 1 and 2

**Figure 2:**
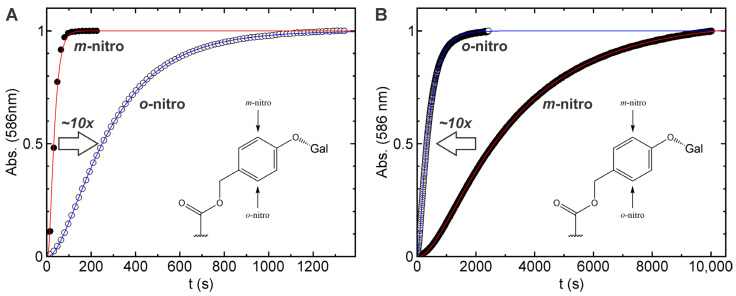
Comparison of enzymatic activation (**A**) and photoactivation (**B**) rates of carboxylic acids **1** and **2.** The structures of self-detachable 3- or 2-nitro-4-oxybenzyl groups are shown in blue (for enzymatic activation) in Scheme 1.

Both probes (**1** and **2**, Scheme 1) have linked β-galactose moieties and are activatable by the action of β-Gal enzyme. In a comparative study of enzymatic activation, we observed that under similar conditions, probe **1** underwent ca. ~10 times faster activation than probe **2** (Figure 2A). The main reason for this difference is that the *para*-quinone methide residue (leaving group) in probe **1** is stabilized by the electron-acceptor nitro group. The two structures of *para*-quinone methides that were based on 3- or 2-nitro-4-oxybenzyl groups are shown in blue frames in Scheme 1. The parent *para*-quinone methide O=C_6_H_4_=CH_2_ ↔ ^−^O–C_6_H_4_–CH_2_^+^ has a negative charge for the oxygen atom and a positive one for the CH_2_ group. Thus, the nitro group in the *ortho* position to oxygen has a stabilizing effect, and the same group in the *ortho* position to CH_2_ destabilizes the leaving group. Therefore, probe **1** has a better leaving group than probe **2** and possesses higher affinity for β-Gal [32]. This explanation is appropriate if we assume that the hydrolytic cleavage of a glycosidic bond by β-Gal is the rate-limiting step, and that the stability of the leaving group correlates with the activation energy.

We compared the photoactivation and enzyme activation rates of compounds (**1** and **2**) under identical conditions (Figure 2A,B and Figure S4). To our surprise, the enzymatic probe **1** could also be activated by light, though the activation rate was found to be 10 times lower in comparison to that of probe **2** (Figure 2B). This was fitted to two consecutive steps of photoinduced reaction models (Figure 2B), yielding uncaging quantum yields of 9.2 × 10^−4^ and 8.7 × 10^−3^ for compound **1**, and of 7.5 × 10^−3^ and 7.4 × 10^−2^ for compound **2**. An LCMS analysis of the irradiated solution showed a clean reaction to the expected product (580CP) in both cases (Figure S5).

### 2.5. Labeling and Bio-Imaging 

#### Confocal and STED Imaging with Probe **5**-H-HT

After confirming the performance of probe **1** in cuvette experiments, we studied its ability to trace β-galactosidase activity in living cells. We selected U-2 OS cells expressing *Vimentin–HaloTag* fusion as a model. Vimentin is a type III intermediate filament protein involved in various cellular functions, such as maintaining cell shape, stabilizing cytoskeletal interactions, and regulating cell division and migration [38,39,40]. Vimentin can also modulate immune responses, extracellular matrix adhesions, cellular contractility, and biomechanics [40]. Changes in Vimentin morphology are associated with many pathologies, such as cataracts, Crohn’s disease, rheumatoid arthritis, HIV, and cancer [38,39,40,41]. Vimentin can act as a biomarker and a drug target for some of these conditions [41].

**Figure 3:**
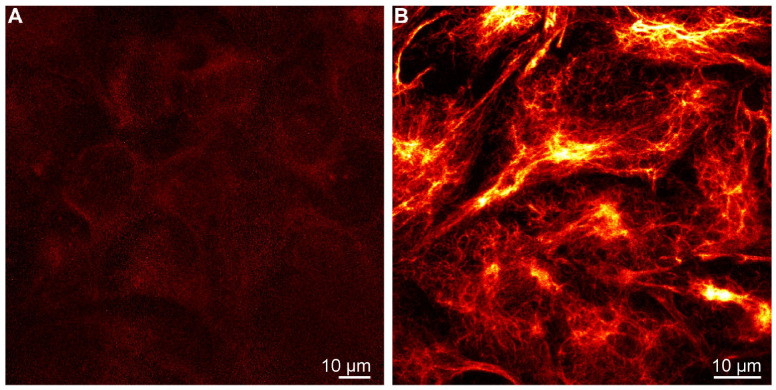
Compound **5**-H-HT in confocal fluorescence microscopy in living cells. Confocal maximum projections of U-2 OS cells with *Vimentin–Halo* fusion from the endogenous locus without (**A**) and with (**B**) overexpression of β-galactosidase from a plasmid. Cells were labeled with 1 µM of compound **5**-H-HT. Scale bars: (**A**,**B**) 10 µm.

In order to overexpress β-galactosidase in U-2 OS cells, the commercially available plasmid pSV-β-galactosidase control vector (Promega, Fitchburg, WI, USA, Cat.Nr. E1081) was used for transfection. Here, the expression of the *lac*Z gene, which encodes the β-Gal, is driven by the simian virus 40 (SV40) as an early promoter and enhancer. Cells with and without overexpressed β-Gal were incubated with compound **5**-H-HT (1 μM) for 30 min and then washed for 30 min. As expected, compound **5**-H-HT showed a significant fluorescence in live cells, with a stronger signal in cells overexpressing β-Gal than in control cells (Figure 3). Probe **5**-H-HT was shown to stay in an OFF state prior to β-Gal activation and did not show any (photo)cytotoxicity. As expected, a high β-Gal level in U-2 OS cells upon overexpression initiates the hydrolysis of glycosidic linkage between β-D-galactopyranose and the phenyl group of the masking unit, which leads to the reaction cascade (elimination of *para*-quinone methide, hydrolysis of urethane, opening of the *spiro*-lactone ring) and the release of the highly emissive uncaged dye.

Compound **5**-H-HT was demonstrated to be cell-permeable and to specifically label *Vimentin*–Halo fusion proteins in living U-2 OS cells. By means of confocal microscopy, we observed its transformation from a non-emissive to fluorescent state in the presence of β-galactosidase. Due to its covalent binding with the HaloTag, the activated dye did not diffuse from the cells. Due to the red emission of the dye, we planned to use the probe in super-resolution STED imaging.

**Figure 4:**
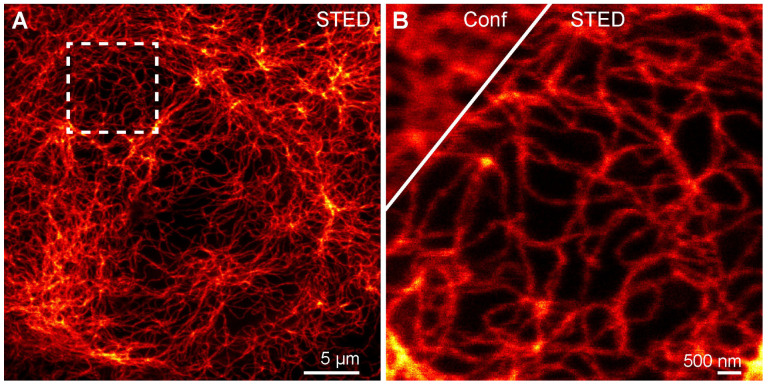
Compound **5**-H-HT in live cell STED microscopy. Confocal and STED images of a U-2 OS cell with *Vimentin–Halo* fusion from the endogenous locus, and overexpressing β-galactosidase from a plasmid. Cells were labeled with 1 µM of compound **5**-H-HT. (**A**) STED image, (**B**) inset from A with Conf. and STED. Scale bars: (**A**) 5 µm; inset (**B**) 500 nm.

We tested this assumption by using living U-2 OS cells that displayed the *Vimentin–Halo* fusion protein and overexpressed β-galactosidase from a plasmid. Labeling was performed as described above, employing 1 µM of compound **5**-H-HT for 30 min followed by a washing step. Subsequently, the living cells were imaged in a STED microscope (Figure 4). The excitation of the dye was performed with a 561 nm laser, and the super-resolution effect was achieved by using a very powerful 775 nm STED laser and detection in the 580–630 nm range. It became apparent that enzymatically activated compound **5**-H-HT clearly produces *Vimentin*–Halo fusions with improved optical resolutions and excellent signal-to-noise ratios, providing diffraction-unlimited images, in comparison with its confocal counterpart (Figure 4B).

### 2.6. Confocal and STED Super-Resolution Imaging with Probe **7**-H-HT

**Figure 5:**
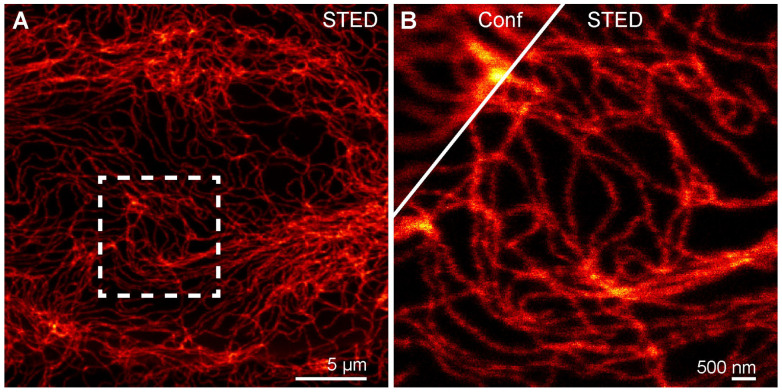
Compound **7**-H-HT in live cell confocal and STED microscopy. Confocal and STED image of a U-2 OS cell with *Vimentin–Halo* fusion from the endogenous locus and additionally overexpressing β-galactosidase from a plasmid. Cells were labeled with 1 µM of compound **7**-H-HT. (**A**) STED image, (**B**) inset with Conf. and STED. (**A**) 5 µm; Inset (**B**) 500 nm.

After proving the enzymatic and photoactivation ability of probe **2** in cuvette experiments (Figure 2), we studied the behavior of probe **2** in live cell experiments and its ability to sense β-Gal. We expect that the dual-responsive probe **7**-H-HT should also perform well in live cells in terms of labeling, β-Gal sensing, and bio-imaging. For this, U-2 OS cells expressing *Vimentin–Halo* and overexpressing β-Gal were incubated with compound **7**-H-HT (1 μM) for 30 min and then washed for 30 min. As expected, a high β-Gal level in U-2 OS cells led to hydrolysis of the glycosidic linkage between β-D-galactopyranose and the phenyl group of the masking unit, the release of free dye, and thus the detection of β-Gal activity (Figure 5). 

By means of confocal microscopy, we observed that compound **7**-H-HT was cell-permeable and specifically labeled U2OS cells that were stably expressing Vimentin–HaloTag fusion protein. Due to covalent labeling of the HaloTag, the activated dye was retained at the site of labeling inside the cells. The U-2 OS cells incubated with the probe **7**-H-HT were imaged in a STED microscope under conditions similar to those of probe **5**-H-HT. The enzymatically activated dye produced good-quality STED images of *Vimentin*–Halo fusions (Figure 5).

## 3. Conclusions

Biocompatible, water-soluble, and cell-permeable fluorescent probes for detection and imaging enzyme activities in organelles and tissues are in high demand in biomedical research. When assessing and tracking enzyme activities in living cells over time, it is important to retain fluorescent reporters inside cell. There are studies [18,22] addressing this challenge, but they have restrictions and drawbacks [23,24]. Our approach is based on the covalent attachment of the probe (non-activated fluorescent reporter) to the target (a certain protein in a living cell). Therefore, this precludes the undesired diffusion of the probe (non-activated and activated) out of the cell and can enable the placid observation of cellular processes, with an option of super-resolution microscopy. 

We designed and prepared β-Gal-responsive “caged” fluorescent probes **1** and **2** along the straightforward, short, and high-yielding synthesis routes. The probes are structurally similar; they differ only in the position of the nitro group in the phenyl ring of the cleavable group. We compared photoactivation and enzyme activation rates of compounds (**1** and **2**) under identical conditions. The relative activation rates depend on the position of the acceptor (nitro) group in *para*-quinone methide O=C_6_H_4_=CH_2_ ↔ ^−^O–C_6_H_4_–CH_2_^+^, and these regularities may be used in the structural design of other enzyme and photoactivatable probes. Both probes displayed a strong increase in emission intensity (from orange to red) upon enzymatic activation with β-galactosidase in an aqueous buffer solution. The conjugation of the probes with a HaloTag^TM^ ligand enabled the specific labeling of a Vimentin–HaloTag fusion protein in live cells. In a more general sense, this approach may be used in designing photoactivatable and/or enzyme-responsive fluorescent probes comprising bright and photostable dyes and targeting enzyme in living system by means of super-resolution microscopy (with variable caging groups based on urethane protection). Importantly, the probes **1** and **2** were found to be biocompatible and cell-permeable. Probe **2** is more responsive to light than probe **1**. Therefore, it is used as a reference in experiments when the enzymatic activity is lower, but a maximal level of observable emission is required for quantitation. We believe that the modular approach presented in this work is suitable for the development of other probes targeting various proteins of interest (by incorporating suitable tags) and for detecting and measuring the levels of enzyme activities, other than those of β-galactosidase. For that, the cage groups may be modified (e.g., sugar structures changed). The enzymatic response can be traced by means of confocal and super-resolution fluorescence microscopy, thereby providing sensitive diagnostic tools for the diseases and pathological states. 

## 4. Materials and Methods

### 4.1. General 

**NMR spectra** were recorded at 25 °C with an Agilent 400-MR spectrometer at 400 MHz (^1^H), and 100.6 MHz (^13^C). ^13^C NMR spectra (125 MHz) were also acquired using a Bruker Avance III HD 500 MHz (BBO Prodigy probe) instrument. Chemical shifts (δ) are reported in ppm. All ^1^H-NMR spectra are referenced to tetramethylsilane (TMS; δ = 0 ppm) using the signals of added TMS (0.03% *v*/*v*) or the residual proton signal of CHCl_3_ (7.26 ppm) in CDCl_3_, CHD_2_OD (3.31 ppm) in CD_3_OD, CHD_2_CN (1.94 ppm) in CD_3_CN, and DMSO-d_5_ (2.50 ppm) in DMSO-d_6_. ^13^C NMR spectra are referenced to TMS (δ = 0 ppm) using the signals of added TMS (0.03% *v*/*v*) or the solvent: CDCl_3_ (77.16 ppm), CD_3_OD (49.00 ppm), or DMSO-d_6_ (39.52 ppm). Multiplicities of signals are described as follows: s = singlet; d = doublet; t = triplet; q = quartet; p = pentet; m = multiplet or overlap of non-equivalent resonances; and br. = broad signal. Coupling constants (*J*) are given in Hz.

**ESI-MS** were recorded on a Varian 500-MS spectrometer (Agilent, Santa Clara, CA, USA). **ESI-HRMS** were recorded on a MICROTOF spectrometer (Bruker, Billerica, MA, USA) equipped with an ESI ion source (Apollo, Chennai, India) and a direct injector with an LC autosampler known as Agilent RR 1200.

**High-performance liquid chromatography:** Analytical HPLC was performed on a Knauer Azura liquid chromatography system with a binary P6.1L pump, UV diode array detector DAD 6.1L, an injection valve with a 20 μL loop, and two electrical switching valves V2.1S with 6-port multiposition valve heads. Analytical columns: Knauer Eurospher II 100-5 C18, 5 μm, 150 × 4 mm or Interchim Uptisphere Strategy C18-HQ, 10 μm, 250 × 4.6 mm, and flow rate 1.2 mL/min, unless stated otherwise.

**The preparative HPLC reverse phase** was performed on an Interchim puriFlash 4250 2X preparative HPLC/Flash hybrid system (Advion-Interchim Scientific, Montluçon, France) with a 2 mL or 5 mL injection loop, a 200–800 nm UV-Vis detector, and an integrated ELSD detector. Preparative columns: Interchim Eurosphere II C18H 5 μm, 250 × 16 mm, flow rate 10 mL/min; and Eurosphere II C18, 5 μm, 250 × 20 mm, and flow rate 20 mL/min, unless specified otherwise.

**Preparative flash column chromatography:** Automated flash column chromatography was performed in the normal phase with an Isolera One system (Biotage AG, Stockholm, Sweden) using commercially available cartridges (RediSep Rf series from Teledyne ISCO, Puriflash Silica HP 30 μm series from Interchim and Biotage Sfaer silica HC) and the solvent gradient was indicated.

### 4.2. Synthetic Procedures

The intermediate compound **3** was prepared according to a procedure described previously [26]. Synthetic protocols for carbamate **4** (Scheme S2), compound **5**-H-HT (Scheme 2), and carbamate **6** (Scheme S4), as well as the complete synthesis results of probe **2** and **7**-HT (Scheme 2), are provided in the Supporting Information (Schemes S5 and S6).

### 4.3. Probe 1

#### 4.3.1. Compound **5**-Ac-*^t^*BuO 

A mixture of compound **3** (20 mg, 28 µmol, 1 eq), compound **4** (38 mg, 66 µmol, 2.4 eq), Jackie Phos Pd G3 (4.8 mg, 4.1 µmol, 15 mol%), Jackie Phos Ligand (3.3 mg, 4.1 µmol, 15 mol%), Cs_2_CO_3_ (25.5 mg, 80 µmol, 2.8 eq), and freshly dried 3A MS (7 beads) were taken in a microwave vial (5 mL) and flushed with argon. Degassed dry toluene (1 mL) was added; the vial was sealed and heated at 110 °C for 16 h in an oil bath. Upon cooling, the resulting mixture was filtered on celite and washed with EtOAc (5×), and the filtrate was evaporated. The residue was purified via flash column chromatography on silica gel using 0–100% ethyl acetate in *n*-hexane to obtain compound **5**-Ac-*^t^*BuO (25 mg, yield: 58%) as an off-white solid. ^1^H NMR (400 MHz, CDCl_3_) δ 8.21 (dd, *J* = 8.0, 1.4 Hz, 1H), 8.07 (dd, *J* = 8.0, 0.7 Hz, 1H), 7.73 (t, *J* = 2.3 Hz, 2H), 7.65 (s, 1H), 7.58 (s, 2H), 7.36 (brs, 2H), 7.21 (dd, *J* = 8.8, 2.6 Hz, 2H), 7.08–7.01 (m, 2H), 6.74 (dd, *J* = 8.5, 1.0 Hz, 2H), 5.55–5.43 (m, 8H), 5.11–5.11 (m, 4H), 4.20–4.11 (m, 6H), 3.35 (s, 6H, N*CH_3_*), 2.18, 2.18 (s, 6H, CO*CH_3_*), 2.06, 2.06 (s, 6H, CO*CH_3_*), 2.05, 2.04 (s, 6H, CO*CH_3_*), 2.01, 2.01 (s, 6H, CO*CH_3_*), 1.84 (s, 3H, C(*CH_3_*)_2_), 1.72 (s, 3H, C(*CH_3_*)_2_), 1.55 (s, 9H, C(*CH_3_*)_3_). ^13^C NMR (101 MHz, CDCl_3_) δ 170.5, 170.4 (C), 170.1 (C), 170.0 (C), 169.3 (C), 169.2 (C), 164.1 (C), 156.3 (C), 154.7 (C), 154.5 (C), 148.1 (C), 145.8 (C), 143.9 (C), 143.9 (C), 138.2 (C), 130.7 (CH), 130.5 (CH), 129.2 (C), 128.5 (CH), 126.9 (C), 125.3 (CH), 124.8 (CH), 124.1 (CH), 122.4 (CH), 122.2 (CH), 112.8, 112.7 (CH), 99.0 (CH), 85.6 (C), 82.7 (C), 71.6, 71.5 (CH), 70.6 (CH), 68.3 (CH), 66.9 (CH), 64.1, 64.1 (CH_2_), 61.6, 61.6 (CH_2_), 38.3 (C), 37.6 CH_3_), 34.8 (CH_3_), 33.1 (CH_3_), 28.0 (CH_3_), 20.7, 20.7 (CH_3_), 20.6 (CH_3_), 20.6 (CH_3_), 20.5 (CH_3_). HRMS (ESI) calcd for C_74_H_78_N_4_O_32_ [M+Na]^+^ 1557.4491, found 1557.4499.

#### 4.3.2. Compound **5**-Ac-OH

Trifluoroacetic acid (0.3 mL) was added dropwise to a solution of **5**-Ac-*^t^*BuO (10 mg, 6.5 µmol) in CH_2_Cl_2_ (1.5 mL) at rt. The resulting solution was stirred at room temperature for 16 h. The reaction mixture was co-distilled with toluene (3×) and then with acetonitrile on rotary evaporator, and was dried under vacuum to obtain compound **5**-Ac-OH (9 mg, yield: 98%) as a light brown solid. ^1^H NMR (400 MHz, CD_3_CN) δ 8.27 (dd, *J* = 8.0, 1.3 Hz, 1H), 8.12 (dd, *J* = 8.0, 0.8 Hz, 1H), 7.70 (t, *J* = 2.2 Hz, 4H), 7.59 (dd, *J* = 1.4, 0.8 Hz, 1H), 7.44 (brs, 2H), 7.27 (d, *J* = 8.9 Hz, 2H), 7.13 (dt, *J* = 8.7, 1.8 Hz, 2H), 6.82 (d, *J* = 8.6 Hz, 2H), 5.44 (dt, *J* = 3.4, 1.0 Hz, 2H), 5.39 (brs, 4H), 5.37–5.30 (m, 4H), 5.23–5.15 (m, 2H), 4.33–4.26 (m, 2H), 4.13 (dt, *J* = 5.8, 1.7 Hz, 4H), 3.31 (s, 6H), 2.15, 2.14 (s, 6H), 2.02, 2.01 (s, 6H), 1.98, 1.97 (s, 6H), 1.95, 1.95 (s, 6H), 1.74 (s, 3H), 1.66 (s, 3H). ^13^C NMR (125 MHz, CD_3_CN) δ 171.2 (C), 171.1 (C), 170.8, 170.7 (C), 170.7 (C), 170.6 (C), 169.9 (C), 166.3 (C), 157.3 (C), 156.0 (C), 155.4 (C), 149.3 (C), 146.5 (C), 145.2 (C), 137.6 (C), 131.9, 131.9 (CH), 131.8 (CH), 130.1 (C), 129.1 (CH), 129.0 (CH), 127.5 (C), 126.6 (CH), 125.4 (CH), 125.3 (CH), 123.0 (CH), 113.5, 113.5 (CH), 99.4, 99.4 (CH), 86.1 (C), 72.4, 72.4 (CH), 71.3 (CH), 69.2, 69.2 (CH), 68.2 (CH), 64.6, 64.61 (CH_2_), 62.5, 62.5 (CH_2_), 39.1 (C), 38.0 (CH_3_), 34.5 (CH_3_), 33.7 (CH_3_), 20.9, 20.9 (CH_3_), 20.8, 20.8 (CH_3_), 20.7 (CH_3_), 20.7 (CH_3_). HRMS (ESI) calcd for C_70_H_70_N_4_O_32_ [M+Na]^+^ 1501.3865, found 1501.3744.

### 4.4. Compound **1**

Compound **5**-Ac-OH (6 mg, 3.4 µmol), LiOH*H_2_O (2.4 mg, 56 µmol), MeOH (0.7 mL) and H_2_O (0.7 mL) were taken in a 5 mL round-bottom-flask and stirred at room temperature for 1.5 h. The reaction mixture was acidified with aq 1M HCl and concentrated under reduced pressure. The crude mixture was subjected to preparative HPLC (16 mm column C18, A:B 70:30 → 0:100 over 25 min, A–water + 0.1% TFA, B—acetonitrile + 0.1% TFA) to obtain compound **1** (3.0 mg, yield 70%) after lyophilization as an off-white solid. Analytical HPLC: 97% (peak area). ^1^H NMR (400 MHz, CD_3_OD) δ 8.30 (dd, *J* = 8.0, 1.3 Hz, 1H), 8.16–8.11 (m, 1H), 7.79–7.66 (m, 4H), 7.61 (s, 1H), 7.38 (d, *J* = 13.0 Hz, 4H), 7.14 (dd, *J* = 8.6, 2.1 Hz, 2H), 6.82 (d, *J* = 8.6 Hz, 2H), 5.41 (s, 4H), 4.96 (dd, *J* = 9.0, 7.7 Hz, 2H), 3.90 (t, *J* = 3.0 Hz, 2H), 3.85–3.77 (m, 2H), 3.76–3.67 (m, 6H), 3.60 (ddd, *J* = 9.7, 3.4, 1.9 Hz, 2H), 3.34 (s, 6H), 1.75 (s, 3H), 1.67 (s, 3H). HRMS (ESI) calcd for C_54_H_54_N_4_O_24_ [M+Na]^+^ 1165.3020, found 1165.3039.

### 4.5. Enzymatic Activation and Optical Characterization

β-galactosidase from *E. coli* (Grade VIII, lyophilized powder, ≥500 units/mg protein, #G5635) and 2-nitrophenyl β-D-galactopyranoside (ONPG, #N1127) were purchased from Sigma Aldrich, St. Louis, MO, USA. Dithiothreitol (DTT, #1610610) was purchased from Bio Rad. Stock solutions were prepared in 100 mM (pH = 7) of aq. phosphate buffer at a concentration of 1000 units/mL for the enzyme, in 20 mM for ONPG, and in 1 M for DTT. A 2 mM stock solution of compound **1** was prepared in DMSO.

The enzymatic activity of β-galactosidase with ONPG (Scheme S1) and with compound **1** (separate experiments) was studied in a previously described home-built setup [42] (Figure 2 and Figure S1). In brief, the sample was stirred and thermostatized during the reaction in a Peltier-based temperature-controlled cuvette holder (Luma 40, Quantum Northwest, Inc., Lake, WA, USA), while the absorption and emission of the sample’s solution was monitored at desired intervals with a fiber-based spectrometer (Flame-S-UV-Vis-ES, Ocean Insight, Orlando, FL, USA). For absorption measurements, deuterium and tungsten halogen sources were used for illumination (DH-2000-BAL, Ocean Insight), and fluorescence excitation (compound **1**) was performed in a 90° configuration with an LED source (M530 L3, Thorlabs Inc., Newton, NJ, USA). For screening, the substrate and enzyme concentration (Figures S2 and S3) absorption measurements were performed in a plate reader (CLARIOstar^®^ Plus, BMG Labtech, Ortenberg, Germany) operated with CLARIOstar^®^ software 5.4. The data were further processed in MARS software (BMG Labtech), and then analyzed and plotted with OriginPro 2020b (OriginLab Corporation, Northampton, MA, USA).

### 4.6. Photoactivation and Optical Characterization

The photolysis of compounds **1** and **2** was performed in the same setup used for enzymatic activation. In this case, a 365 nm LED (M365 L2, Thorlabs Inc.) at a 90° configuration was used to irradiate the samples for a set period of time, and then switched off to measure the absorption and the emission spectra at the corresponding irradiation time (Figure 2B and Figure S4). The resulting solutions after the irradiation was concluded (Figure S5) were injected (10 µL sample) via a Shimadzu LC-MS system, containing a Hypersil GOLD 50 × 2.1 mm 1.9 μm analytical column. The following method was used: isocratic 90:10 A:B for 2 min, then gradient 90:10–1:99 A:B over 5 min, then isocratic 1:99 A:B for 2 min, where solvent A is water + 0.1% *v*/*v* HCO_2_H, and solvent B is acetonitrile + 0.1% *v*/*v* HCO_2_H. A solvent flow rate of 0.5 mL/min was used, as well as a column temperature of 30 °C.

### 4.7. Labeling and Bio-Imaging

Cell culture of monoclonal *Vimentin–Halo*-expressing U-2 OS cells and transformation with the pSV-β-galactosidase control vector: Human monoclonal *Vimentin–Halo*-expressing osteosarcoma cells (U-2 OS) [43,44] were cultivated in McCoy’s medium (Thermo Fisher Scientific, Waltham, MA, USA), supplemented with 10% (*v*/*v*) fetal bovine serum (Thermo Fisher Scientific, Waltham, MA, USA) and 1% (*v*/*v*) sodium pyruvate (Sigma Aldrich, St. Louis, MO, USA), in a humidified 5% CO_2_ incubator at 37 °C. Transfection with the pSV-β-galactosidase control vector (Promega, Fitchburg, WI, USA, Cat.Nr. E1081) was performed using TurboFect^TM^ (Thermo Fisher Scientific, Waltham, MA, USA) according to the manufacturer’s protocol.

Staining of living U-2 OS cells expressing *Vimentin–Halo*: The day before transfection, 10,000 U2-OS cells per chamber of a chamber slide (ibidi GmbH, Gräfelfing, Germany; µ-Slide 8 Well Glass Bottom) were seeded in McCoy’s medium (Thermo Fisher Scientific, Waltham, MA, USA) supplemented with 10% (*v*/*v*) fetal bovine serum (Thermo Fisher Scientific, Waltham, MA, USA) and 1% (*v*/*v*) sodium pyruvate (Sigma Aldrich, St. Louis, MO, USA). Some 24 hours after transfection with the pSV-β-galactosidase control vector or mock treatment, respectively, staining with the Halo ligands was performed. Washing and imaging were performed in Opti-MEM^TM^ (Thermo Fisher Scientific, Waltham, MA, USA). For labeling, the cells were incubated with a 1 µM solution of a probe for 30 min at 37 °C in a humidified 5% CO_2_ incubator. Then, the cells were washed with Opti-MEM three times for a total period of 30 min, and afterwards imaged directly.

Confocal and STED microscopy of living U-2 OS cells: Confocal light microscopy was performed using a Leica TCS SP8 laser-scanning microscope (Leica Microsystems, Wetzlar, Germany). STED microscopy was performed using a quad scanning STED microscope (Abberior Instruments, Göttingen, Germany) equipped with a UPlanSApo 100×/1.40 Oil objective (Olympus, Tokyo, Japan). For dye excitation, a laser with an emission wavelength of 561 nm was used. To achieve super-resolution imaging, a STED laser with the emission wavelengths of 775 nm and a repetition rate of 40 MHz was applied. For detection, a filter set designed for a detection range between 580 and 630 nm was used. With the exception of contrast stretching, no further image processing was applied.

## Data Availability

The data presented in this study are available in the article and Supplementary Materials.

## Supplementary Materials

The following supporting information can be downloaded at: https://www.mdpi.com/article/10.3390/molecules29153596/s1, Scheme S1: Enzyme control with ONPG; Figure S1: Activation of ONPG and compound **1;** Figure S2: Activation of compound **1** at different substrate (A) and enzyme (B) concentrations; Figure S3: Michaelis–Menten constant (K_M_) for ONPG and compound **1**; Figure S4: Absorption and emission of compound **2**; Figure S5: LCMS after photolysis of compound **1** and **2**; Scheme S2: Synthesis of carbamate **4**; Scheme S3: Synthesis of compound **5**-H-HT; Scheme S4: Synthesis of carbamate **6**; Scheme S5: Synthesis of compound **2**; Scheme S6: Synthesis of compound **7**-H-HT; Supplementary references; ^1^H NMR spectra and ^13^C NMR spectra of synthesized compounds.
