# Supplementary material for: β-Galactosidase- and Photo-Activatable Fluorescent Probes for Protein Labeling and Super-Resolution STED Microscopy in Living Cells
Source: Molecules. 2024 Jul 30;29(15):3596. doi: 10.3390/molecules29153596 (PMC11314211; doi:10.3390/molecules29153596)

Supporting Information

β-Galactosidase- and Photo-Activatable Fluorescent Probes for Protein Labeling and Super-resolution STED Microscopy in Living Cells

Taukeer A. Khan*[a], Stefan Stoldt[a], Mariano L. Bossi*[b], Vladimir N. Belov*[a] and Stefan W. Hell[a,b]

[a] Dr. T. A. Khan, Dr. S. Stoldt, Dr. V. N. Belov, Prof. Dr. S. W. Hell
Department of NanoBiophotonics
Max Planck Institute for Multidisciplinary Sciences (MPI-NAT)
Am Fassberg 11, 37077 Göttingen (Germany)

[b] Dr. M. L. Bossi, Prof. Dr. S. W. Hell
Department of Optical Nanoscopy
Max Planck Institute for Medical Research (MPI-MR)
Jahnstrasse 29, 69120 Heidelberg (Germany)

* Corresponding authors: taukeer.khan@mpinat.mpg.de, vladimir.belov@mpinat.mpg.de, Mariano.Bossi@mr.mpg.de

Table of contents:

1. Activation and optical characterization

3. Synthetic procedures

4. Supplementary references

5. NMR spectra and HPLC traces

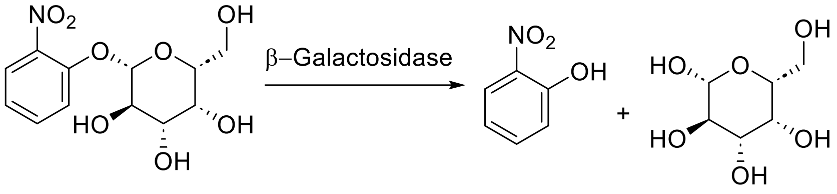

ONPG

Scheme S1: Enzyme control with *o*-nitrophenyl β-D-galactopyranoside (ONPG)

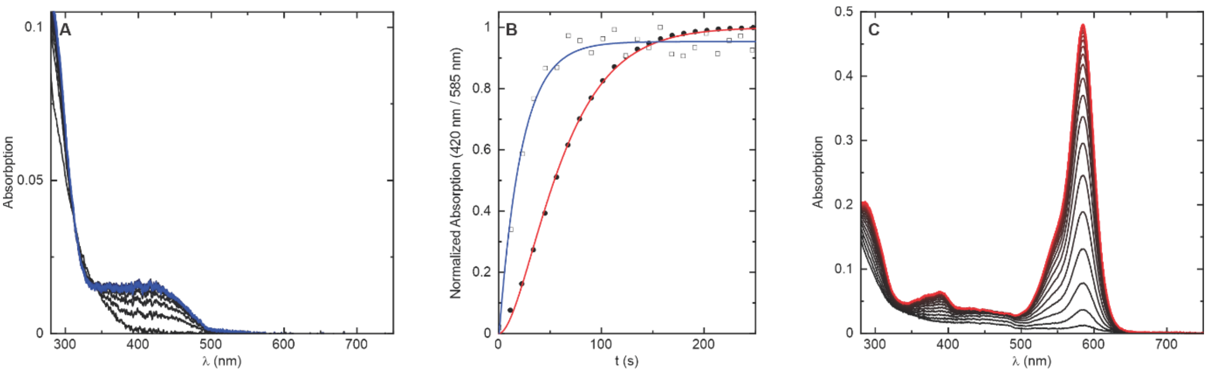

Figure S1: Activation of *o*-nitrophenyl β-D-galactopyranoside (ONPG) and compound **1** (main text) at the same concentration (6.7 μM) in a phosphate buffer (pH = 7) solution at 37 °C with 20 mM dithiothreitol (DTT), upon the addition of β-galactosidase (3 units/ml). (A) Absorption changes of ONPG; (C) Absorption changes of **1**; (B) Normalized absorption changes at 420 nm for ONPG (hollow squares) and at 585 nm for compound **1** (filled circles). The blue and red lines correspond to a mono and a biexponential (with $k_1 = k_2$) fit, respectively.

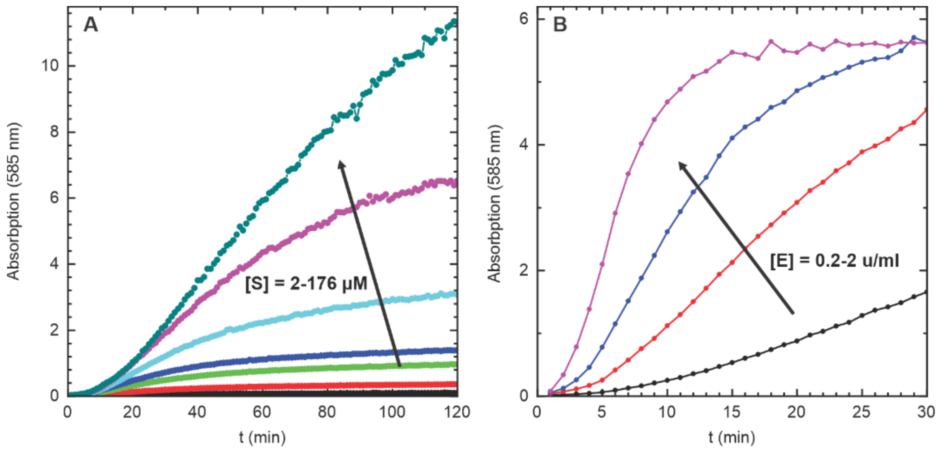

Figure S2: Activation of compound **1** (main text) at different substrate (A) and enzyme (B) concentrations, in aqueous phosphate buffer (pH = 7) solution at 37 °C with 20 mM DTT.

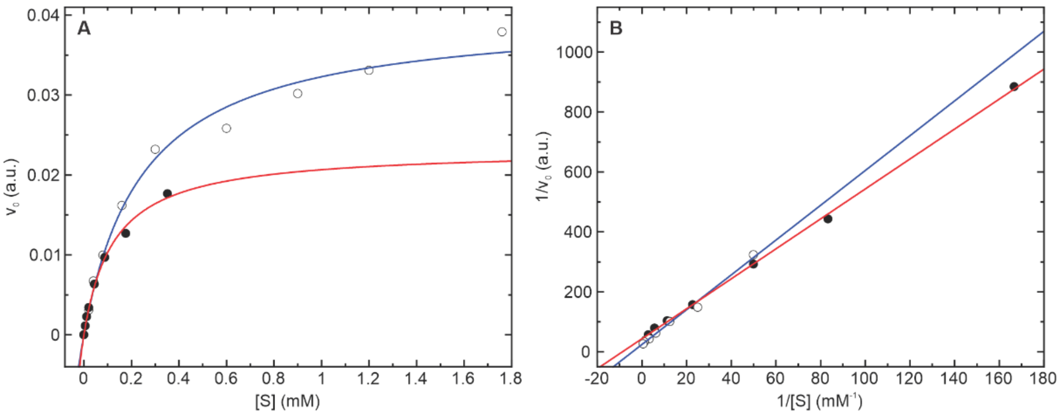

Figure S3: Determination of the Michaelis-Menten constant (K_M) for the reference substrate (ONPG) and compound **1** (main text). Initial reaction rates calculated from linear fits of the absorption changes at 420 nm for ONPG (hollow circles) and at 450 nm for compound **1** (filled circles), from the experiments performed in a plate-reader at different substrate concentrations (T = 37 °C, [enzyme] = 0.2 units/ml). (A) Experimental values of initial rates with a fit to the Michaelis equation (blue and red lines, respectively). (B) Corresponding Lineweaver–Burk plots. The calculated Km is 0239 mM for ONPG and 0.115 mM for compound **1**. The v_{MAX} are not compared because the absorption coefficient of compound **1** at 450 nm is unknown (the rates are expressed in different units).

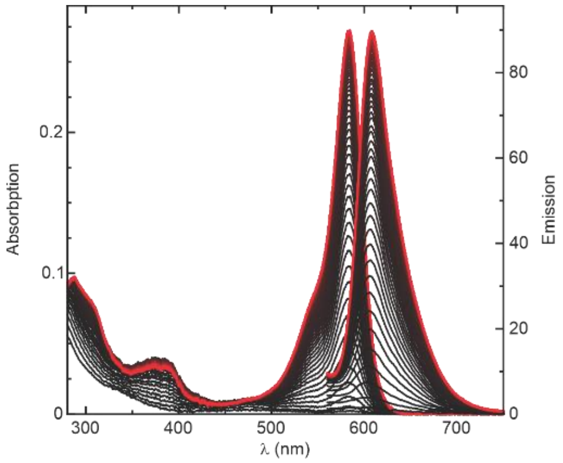

Figure S4. Photo-activation of the carboxylic acid, **2.** Absorption and emission changes of a 1.3 µM solution of **2** in aqueous phosphate buffer (pH = 7) at 37 °C.

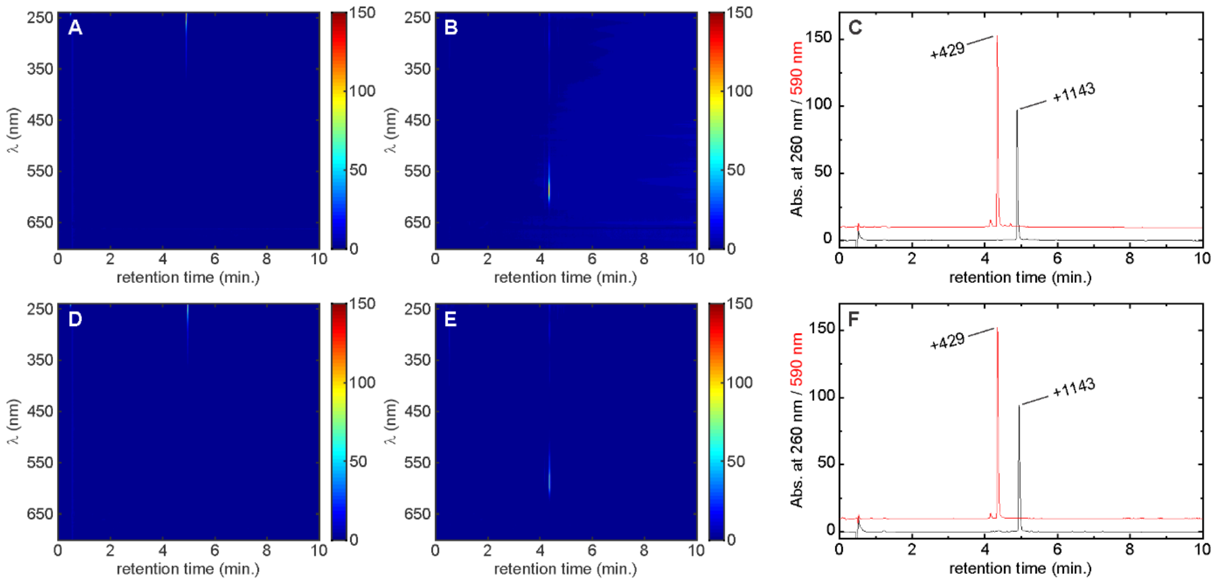

Figure S5: LCMS analysis of the reaction mixtures after photolysis. Chromatograms (2D maps) of a solution of compound **1** before (A) and after (B) irradiation. The chromatogram at the indicated wavelengths (260 nm and 590 nm for the starting and the irradiated solution, respectively) are presented in C. The m/z observed is indicated for each peak. The corresponding results obtained for compound **2** are presented in D-E and F.

Synthesis of Intermediates and target compounds

Scheme S2 (Synthesis of carbamate **4**)

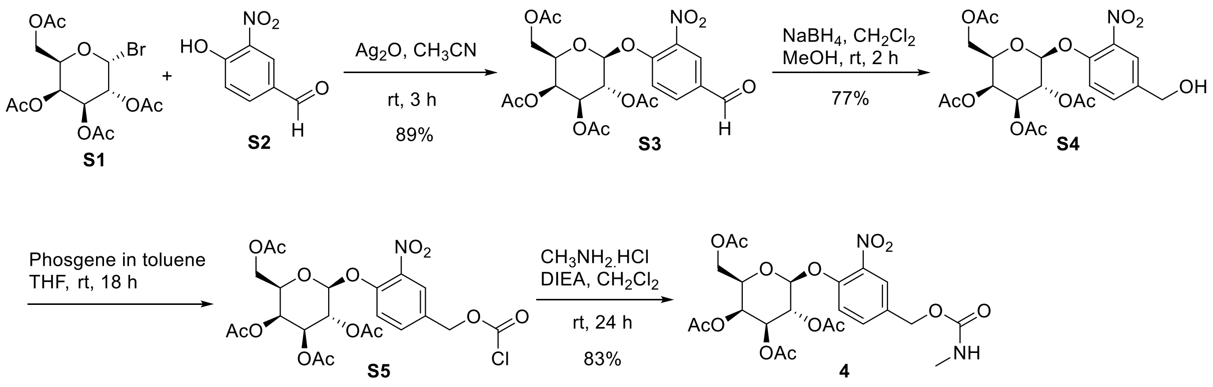

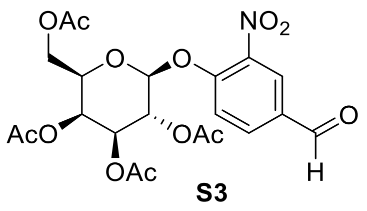

(2R,3S,4S,5R,6S)-2-(Acetoxymethyl)-6-(4-formyl-2-nitrophenoxy)tetrahydro-2H-pyran-3,4,5-triyl triacetate (S3): [S1] 4-Hydroxy-3-nitrobenzaldehyde (500 mg, 3.0 mmol) and silver oxide (700 mg, 3.0 mmol) were added to a solution of 2,3,4,6-tetra-*O*-acetyl-*α*-D-galactopyranosylbromide (from Sigma Aldrich, 865 mg, 2.1 mmol) in anhydrous acetonitrile (25 mL). The reaction mixture was stirred in the dark for 3 h at room temperature. The resulting precipitate was filtered on Celite®Hyflo Super-cel, filtrate was concentrated and the residue was dissolved in ethyl acetate. And then the solution was washed with saturated sodium bicarbonate solution and brine. The organic layer was dried over $MgSO_4$ and evaporated. This crude product (solid) was washed with 10% EtOAc in *n*-hexane to obtain the desired compound **S3** (930 mg, yield 89%) as a light pale-yellow solid.

^{1}H NMR (400 MHz, $CDCl_3$) δ 9.98 (s, 1H), 8.31 (d, *J* = 2.0 Hz, 1H), 8.07 (dd, *J* = 8.6, 2.0 Hz, 1H), 7.49 (d, *J* = 8.7 Hz, 1H), 5.59 (dd, *J* = 10.5, 7.8 Hz, 1H), 5.50 (dd, *J* = 3.5, 1.1 Hz, 1H), 5.21 (d, *J* = 7.9 Hz, 1H), 5.13 (dd, *J* = 10.4, 3.4 Hz, 1H), 4.31 – 4.11 (m, 3H), 2.20 (s, 3H), 2.13 (s, 3H), 2.09 (s, 3H), 2.03 (s, 3H).

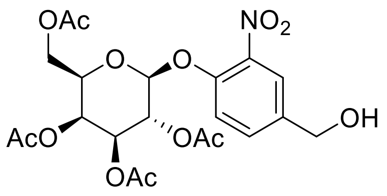

S4

(2R,3S,4S,5R,6S)-2-(Acetoxymethyl)-6-(4-(hydroxymethyl)-2-nitrophenoxy)tetrahydro-2H-pyran-3,4,5-triyl triacetate (S4): Sodium borohydride (137 mg, 3.6 mmol) was added to a solution of compound **S3** (900 mg, 1.8 mmol) in CH_2Cl_2/MeOH (95:5, 90 mL). The reaction mixture was stirred at room temperature for 2 h. The reaction was quenched by adding cold water (10 mL), and the solvent was evaporated. More water (50 mL) was added, and the mixture extracted with ethyl acetate (3 x). The organic layer was washed with brine, dried over Na_2SO_4. and concentrated. The residue was washed with diethyl ether and dried under vacuum to yield compound **S4** (690 mg, yield 77% yield) as a white solid. [S1]

^{1}H NMR (400 MHz, $CDCl_3$) δ 7.81 (d, *J* = 2.1 Hz, 1H), 7.53 (dd, *J* = 8.6, 2.2 Hz, 1H), 7.36 (d, *J* = 8.6 Hz, 1H), 5.55 (dd, *J* = 10.5, 7.9 Hz, 1H), 5.47 (dd, *J* = 3.5, 1.2 Hz, 1H), 5.11 (dd, *J* = 10.5, 3.4 Hz, 1H), 5.06 (d, *J* = 8.0 Hz, 1H), 4.74 (s, 2H), 4.31 – 4.15 (m, 2H), 4.07 (ddd, *J* = 7.1, 6.2, 1.2 Hz, 1H), 2.20 (s, 3H), 2.14 (s, 3H), 2.08 (s, 3H), 2.02 (s, 3H).

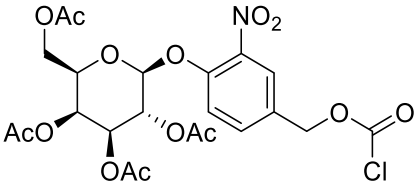

S5

Ac-Gal-*m*-NO$_2$Bn-chloroformate (S5): Phosgene (20% in toluene, 0.126 mL, 0.253 mmol) was added to a solution of compound **S4** (55 mg, 0.11 mmol) in THF (1 mL) at rt, and the reaction mixture was stirred at room temperature for 18 h. The excess of phosgene was removed by purging with argon gas and trapping into Na_2CO_3 solution. The mixture was concentrated and dried under high vacuum to yield the chloroformate (**S5**) (59 mg, crude product). The crude material was used in next step.

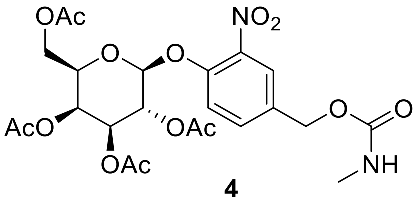

4

Ac-Gal-*m*-NO$_2$Bn-carbamate (4): CH_3NH_2.HCl (39 mg, 0.57 mmol, 1.5 eq) and *N,N*-diisopropylethylamine (0.265 mL, 1.52 mmol, 4 eq) were added to a solution of compound **S5** (214 mg, 0.38 mmol, 1 eq) in CH_2Cl_2 (6 mL) at rt, and reaction mixture was stirred under argon gas at rt for 24 h. The solvent was evaporated, and the residue was taken up in water and extracted with EtOAc (3 ×). The combined organic solutions were washed with brine, dried (Na_2SO_4), filtered and concentrated in vacuo. The crude material was purified on silica gel (Puriflash 12 g) using 0-80% EtOAc in *n*-hexane to afford carbamate **4** (177 mg, yield 83% yield) as a white solid.

^{1}H NMR (400 MHz, $CDCl_3$) δ 7.80 (d, *J* = 2.1 Hz, 1H), 7.52 – 7.50 (m, 1H), 7.33 (d, *J* = 8.6 Hz, 1H), 5.54 (dd, *J* = 10.5, 7.9 Hz, 1H), 5.47 (dd, *J* = 3.4, 1.0 Hz, 1H), 5.09 (dd, *J* = 10.5, 3.4 Hz, 1H), 5.09 (s, 2H), 5.06 (d, *J* = 8.2 Hz, 1H), 4.70 (brs, 1H), 4.27 – 4.14 (m, 2H), 4.06 (t, *J* = 6.6 Hz, 1H), 2.82 (d, *J* = 5.0 Hz, 3H), 2.19 (s, 3H), 2.13 (s, 3H), 2.07 (s, 3H), 2.02 (s, 3H).

^{13}C NMR (101 MHz, $CDCl_3$) δ 170.3, 170.1 (2x), 169.3, 156.4, 148.9, 141.2, 133.1 (2x), 124.5, 119.8, 100.7, 71.4, 70.5, 67.8, 66.7, 64.7, 61.3, 27.6, 20.6 (3x), 20.5.

HRMS (ESI) calcd for $C_{23}H_{28}N_2O_{14}$ $[M+Na]^+$ 579.1433, found 579.1431.

Scheme S3 (Synthesis compound **5**-H-HT)

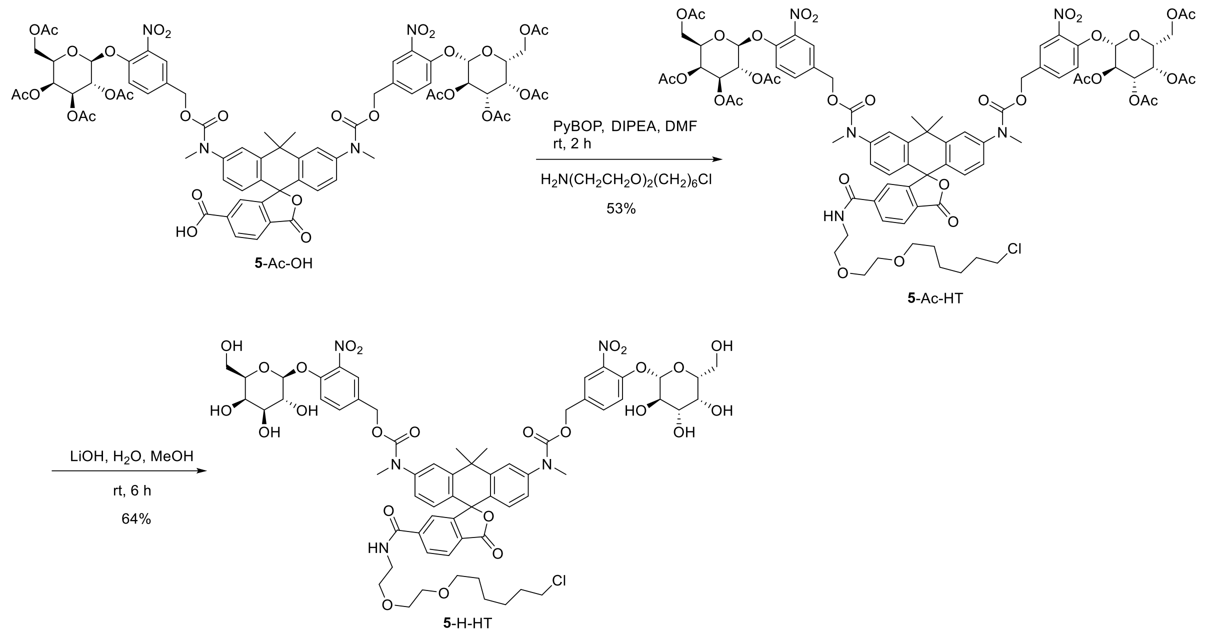

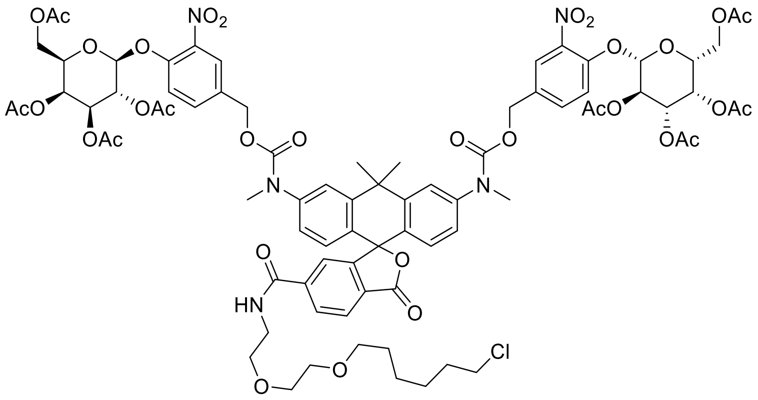

5-Ac-HT

Ac-Gal-(*m*-NO_2-Bn)-580CP-HaloTag (5-Ac-HT): Compound **5**-Ac-OH (6 mg, 4 µmol, 1 eq), HaloTag-Amine (1.4 mg, 6 µmol, 1.5 eq) and *N,N*-diisopropylethylamine (3 µL, 17 µmol, 4 eq) were taken in DMF (1 mL) and then PyBOP (2.6 mg, 5 µmol, 1.2 eq) was added at rt. The reaction mixture was stirred at rt for 2 h, concentrated under vacuum, and the residue was subjected to silica gel chromatography (0–10% MeOH/CH_2Cl_2, linear gradient) to provide compound **5**-Ac-HT (3.6 mg, yield 53%) as a white solid.

^{1}H NMR (400 MHz, CD_3CN) δ 8.07 (qd, *J* = 8.1, 1.1 Hz, 2H), 7.79 (d, *J* = 2.1 Hz, 2H), 7.70 (t, *J* = 1.8 Hz, 2H), 7.59 (dd, *J* = 8.7, 2.2 Hz, 2H), 7.44 – 7.34 (m, 3H), 7.23 (t, *J* = 5.5 Hz, 1H), 7.14 (ddd, *J* = 8.6, 2.3, 0.9 Hz, 2H), 6.79 (dd, *J* = 8.5, 1.2 Hz, 2H), 5.44 (ddd, *J* = 3.5, 2.3, 1.1 Hz, 2H), 5.38 – 5.29 (m, 4H), 5.21 – 5.16 (m, 2H), 5.14 (s, 4H), 4.31 – 4.26 (m, 2H), 4.25 – 4.11 (m, 4H), 3.58 – 3.49 (m, 6H), 3.49 – 3.39 (m, 4H), 3.32 (s, 6H), 3.31 – 3.28 (m, 2H), 2.15 (s, 6H), 2.03 (s, 6H), 2.00, 2.00 (s, 6H), 1.98 (s, 6H), 1.75 (s, 3H), 1.74 – 1.66 (m, 2H), 1.64 (s, 3H), 1.47 – 1.22 (m, 6H).

^{13}C NMR (126 MHz, CD_3CN) δ 171.2 (C), 171.1 (C), 170.8 (C), 170.3 (C), 170.0 (C), 166.4 (C), 155.9 (C), 155.6 (C), 149.6 (C), 146.6 (C), 145.3 (C), 142.5 (C), 141.6 (C), 134.5 (CH), 133.4 (C), 129.6 (CH), 129.2 (CH), 129.2 (CH), 129.0 (C), 128.9 (C), 126.4 (CH), 125.3 (CH), 125.2 (CH), 123.0 (CH), 119.2 (CH), 100.5 (CH), 86.1 (C), 72.4 (CH), 71.5 (CH_2), 71.2 (CH), 70.8 (CH_2), 70.7 (CH_2), 69.8 (CH_2), 68.8 (CH), 68.1 (CH), 66.4 (CH_2), 62.2 (CH_2), 46.2 (CH_2), 40.6 (CH_2), 39.1 (C), 38.0 (CH_3), 34.6 (CH_3), 33.5 (CH_3), 33.2 (CH_2), 30.2 (CH_2), 27.3 (CH_2), 26.1 (CH_2), 20.9 (CH_3), 20.8 (CH_3), 20.79 (CH_3), 20.76 (CH_3).

HRMS (ESI) calcd for $C_{80}H_{90}ClN_5O_{33}$ $[M+Na]^+$ 1706.5099, found 1706.5083.

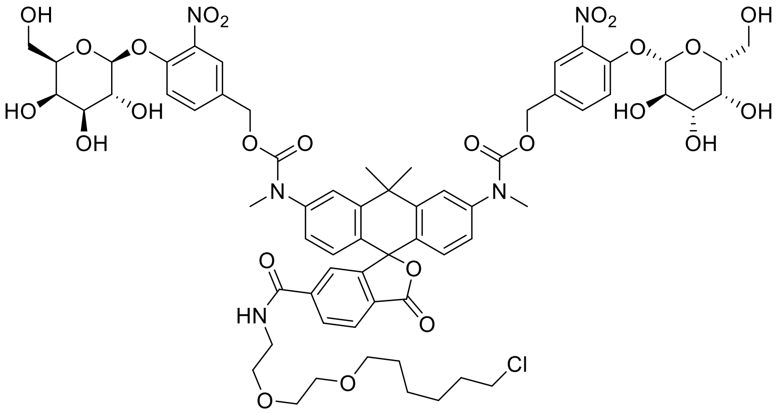

5-H-HT

Gal-(*m*-NO$_2$-Bn)-HaloTag Probe (5-H-HT): A mixture of compound **5**-Ac-HT (3.6 mg, 2 µmol), LiOH*H_2O (3 mg, 71 µmol), MeOH (0.8 ml) and H_2O (0.8 ml) was stirred at rt for 6 h. The reaction mixture was acidified with aq 1M HCl, extracted with EtOAc (6 x), dried (Na_2SO_4) and concentrated. The crude product was purified by prep. HPLC (16 mm column C18, A:B 80:20 → 0:100 over 25 min, A – water + 0.1% TFA, B – acetonitrile + 0.1% TFA) to get compound **5**-H-HT (2.5 mg, yield: 64%) lyophilization as an off-white solid. Analytical HPLC purity: 95%.

^{1}H NMR (400 MHz, DMSO-d_6) δ 8.79 (t, *J* = 5.7 Hz, 1H), 8.21 – 8.10 (m, 2H), 7.86 (d, *J* = 2.1 Hz, 2H), 7.77 (d, *J* = 2.3 Hz, 2H), 7.63 (dd, *J* = 8.7, 2.1 Hz, 2H), 7.52 (t, *J* = 1.0 Hz, 1H), 7.39 (dd, *J* = 8.9, 1.2 Hz, 2H), 7.16 (dd, *J* = 8.5, 2.2 Hz, 2H), 6.66 (d, *J* = 8.6 Hz, 2H), 5.11 (s, 4H), 5.02 (dd, *J* = 7.7, 1.7 Hz, 2H), 3.69 (d, *J* = 3.3 Hz, 2H), 3.62 (t, *J* = 6.2 Hz, 2H), 3.59 – 3.51 (m, 6H), 3.50 – 3.36 (m, 10H), 3.35 – 3.24 (m, 4H), 3.28 (s, 6H) 1.75 (s, 3H), 1.68 – 1.59 (m, 2H), 1.62 (s, 3H), 1.39 (p, *J* = 6.8 Hz, 2H), 1.35 – 1.21 (m, 6H).

HRMS (ESI) calcd for $C_{70}H_{70}N_4O_{32}$ $[M+Na]^+$ 1370.4254, found 1370.4224.

Scheme S4 (Synthesis of carbamate **6**)

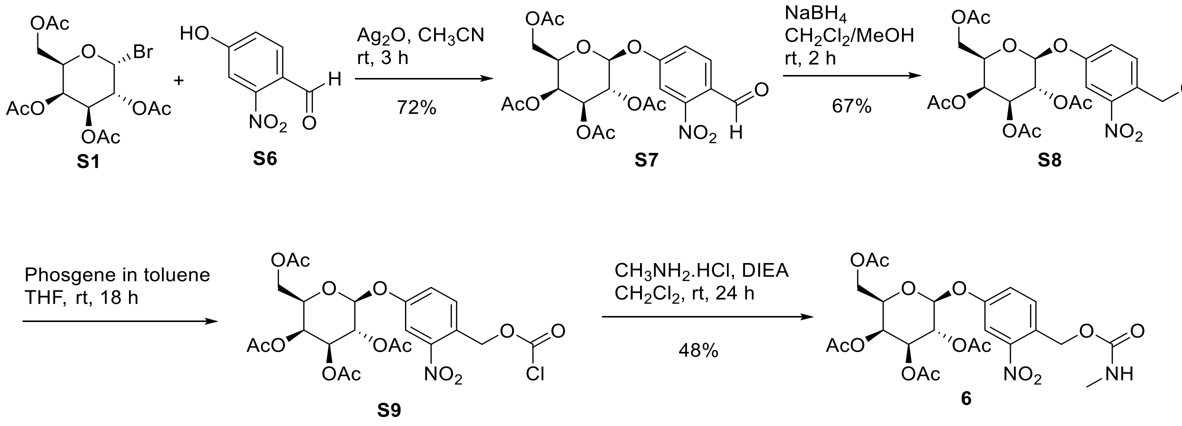

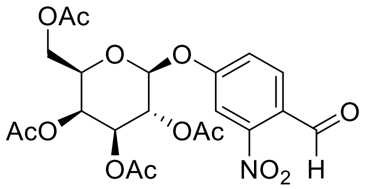

S7

(2R,3S,4S,5R,6S)-2-(Acetoxymethyl)-6-(4-formyl-3-nitrophenoxy)tetrahydro-2H-pyran-3,4,5-triyl triacetate (S7): 2-Nitro-4-hydroxybenzaldehyde (330 mg, 2 mmol) and silver oxide (456 mg, 2 mmol) were added to a solution of 2,3,4,6-tetra-O-acetyl-α-D-galactopyranosylbromide (580 mg, 1.42 mmol) in anhydrous acetonitrile (15 mL). The reaction mixture was stirred in the dark for 18 h at room temperature. The resulting precipitate was filtered on Celite, filtrate was concentrated and the residue was dissolved in ethyl acetate. And then the solution was washed with saturated sodium bicarbonate solution and brine. The organic layer was dried over $MgSO_4$ and evaporated. This crude product was purified by silica gel chromatography (0–60% EtOAc/*n*-Hexane, linear gradient) to obtain the desired compound **S7** (575 mg, Yield: 82%) as an off-white solid.

^{1}H NMR (400 MHz, $CDCl_3$) δ 10.32 (s, 1H), 7.97 (d, *J* = 8.6 Hz, 1H), 7.69 (d, *J* = 2.4 Hz, 1H), 7.34 (dd, *J* = 8.6, 2.5, Hz, 1H), 5.53 (dd, *J* = 10.4, 7.9 Hz, 1H), 5.49 (d, *J* = 3.4 Hz, 1H), 5.23 (d, *J* = 7.9 Hz, 1H), 5.15 (dd, *J* = 10.5, 3.4 Hz, 1H), 4.23 – 4.14 (m, 3H), 2.19 (s, 3H), 2.09 (s, 3H), 2.08 (s, 3H), 2.02 (s, 3H).

^{13}C NMR (101 MHz, $CDCl_3$) δ 186.8 (CHO), 170.4 (CO), 170.0 (CO), 169.9 (CO), 169.2 (CO), 160.1 (C), 151.2 (C), 131.4 (CH), 125.6 (C), 121.8 (CH), 111.9 (CH), 98.6 (CH), 71.9 (CH), 70.5 (CH), 68.1 (CH), 66.8 (CH), 61.7 (CH_2), 20.7 (CH_3), 20.6 (CH_3), 20.6 (CH_3), 20.5 (CH_3).

HRMS (ESI) calcd for $C_{21}H_{23}N_1O_{13}$ $[M+Na]^+$ 520.1062, found 520.1039.

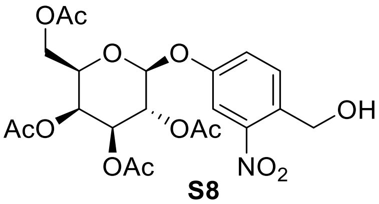

S8

(2R,3S,4S,5R,6S)-2-(Acetoxymethyl)-6-(4-(hydroxymethyl)-3-nitrophenoxy)tetrahydro-2H-pyran-3,4,5-triyl triacetate (S8): Sodium borohydride (84 mg, 2.2 mmol) was added to a solution of compound

S7 (550 mg, 1.1 mmol) in CH_2Cl_2/MeOH (95:5, 55 mL). The reaction mixture was stirred at room temperature for 2 h. The reaction was quenched with cold water (6 mL), and the solvent was evaporated, more water (30 mL) was added and extracted with ethyl acetate (3 x). The organic layer was washed with brine, dried over Na_2SO_4. and concentrated. The product was purified by silica gel chromatography (0–80% EtOAc/*n*-Hexane, linear gradient) to yield compound **S8** (410 mg, 74% yield) as a white solid.

^{1}H NMR (400 MHz, $CDCl_3$) δ 7.76 (d, *J* = 2.6 Hz, 1H), 7.66 (d, *J* = 8.6 Hz, 1H), 7.29 (dd, *J* = 8.5, 2.6 Hz, 1H), 5.55 – 5.44 (m, 2H), 5.17 – 5.08 (m, 2H), 4.92 (s, 2H), 4.21 – 4.11 (m, 3H), 2.19 (s, 3H), 2.09 (s, 3H), 2.08 (s, 3H), 2.02 (s, 3H).

^{13}C NMR (101 MHz, $CDCl_3$) δ 170.5 (CO), 170.1 (CO), 170.0 (CO), 169.3 (CO), 156.1 (C), 148.0 (C), 131.5 (C), 131.2 (CH), 122.9 (CH), 112.7 (CH), 99.2 (CH), 71.6 (CH), 70.6 (CH), 68.3 (CH), 66.9 (CH), 62.1 (CH_2), 61.7 (CH_2), 20.7 (CH_3), 20.6 (CH_3), 20.6 (CH_3), 20.5 (CH_3).

HRMS (ESI) calcd for $C_{21}H_{25}N_1O_{13}$ $[M+Na]^+$ 522.1218, found 522.1198.

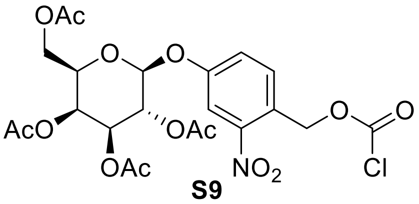

S9

Ac-Gal-*o*-NO$_2$Bn-chloroformate (S9): Phosgene 20% in toluene (0.46 mL, 9.2 mmol) was added to a solution of compound **S8** (200 mg, 0.4 mmol) in dry THF (4 mL) at rt and reaction mixture was stirred at room temperature for 18 h. Excess phosgene was removed by purging argon gas and trapping into Na_2CO_3 solution. The mixture was concentrated and dried under high vacuum to yield the chloroformate (**S9**) (220 mg, crude product). The crude material was used in next step.

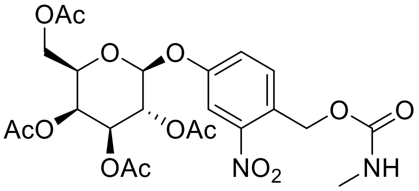

6

Ac-Gal-*o*-NO$_2$Bn-carbamate (6): CH_3NH_2.HCl (39 mg, 0.57 mmol, 1.5 eq) and *N,N*-diisopropylethylamine (0.265 mL, 1.52 mmol, 4 eq) were added to a solution of compound **S9** (213 mg, 0.38 mmol, 1 eq) in CH_2Cl_2 (5 mL) at rt and reaction mixture was further stirred at rt for 24 h under argon gas. The solvent was evaporated and residue was taken up in water and extracted with EtOAc (3 ×). The combined organic solutions were washed with brine, dried (Na_2SO_4), filtered and concentrated in vacuo. The crude material was purified on silica gel (Puriflash 12 g) by Biotage isolera using 0-80% EtOAc in *n*-hexane to afford carbamate **6** (176 mg, 82% yield) as an off-white solid.

^{1}H NMR (400 MHz, $CDCl_3$) δ 7.75 (d, *J* = 2.6 Hz, 1H), 7.52 (d, *J* = 8.6 Hz, 1H), 7.25 (dd, *J* = 8.6 Hz, *J* = 2.6 Hz, 1H), 5.56 – 5.42 (m, 4H), 5.16 – 5.05 (m, 2H), 4.74 (brs, 1H), 4.19 (dd, *J* = 6.2, 3.1 Hz, 2H), 4.16 – 4.08 (m, 1H), 2.83 (d, *J* = 4.9 Hz, 3H), 2.19 (s, 3H), 2.09 (s, 3H), 2.08 (s, 3H), 2.02 (s, 3H).

^{13}C NMR (101 MHz, $CDCl_3$) δ 170.5 (CO), 170.1 (CO), 170.0 (CO), 169.3 (CO), 156.4 (CO, amide), 156.2 (C), 148.1 (C), 130.5 (CH), 127.7 (C), 122.5 (CH), 112.6 (CH), 99.2 (CH), 71.6 (CH), 70.6 (CH), 68.3 (CH), 66.9 (CH), 62.9 (CH_2), 61.7 (CH2), 27.6 (N-CH_3), 20.7 (CH_3), 20.6 (CH_3), 20.6 (CH_3), 20.5 (CH_3).

HRMS (ESI) calcd for $C_{23}H_{28}N_2O_{14}$ $[M+Na]^+$ 579.1433, found 579.1423.

Scheme S5 (Synthesis compound **2**)

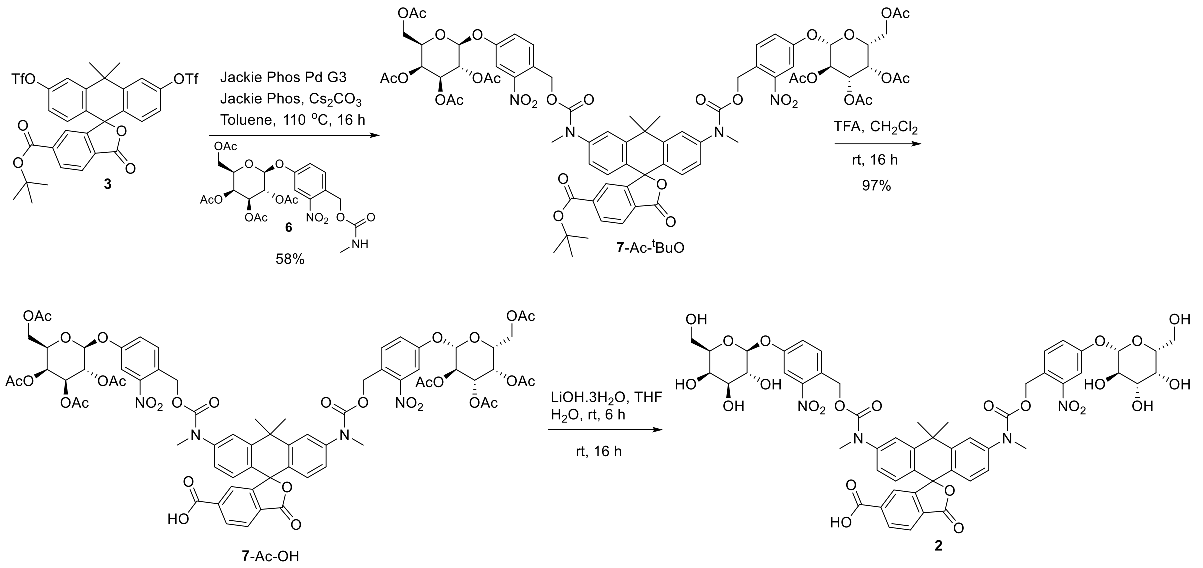

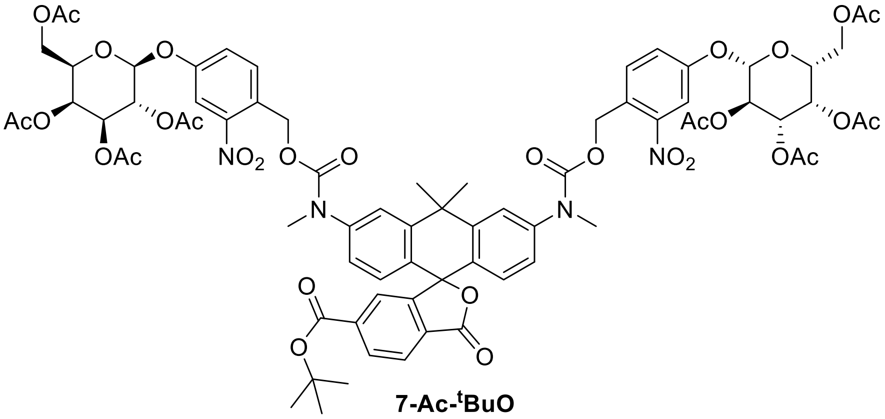

7-Ac-^{t}BuO

Compound 7-Ac-^{t}BuO: A mixture of compound **3**[S2] (20 mg, 28 µmol, 1 eq), compound **6** (38 mg, 66 µmol, 2.4 eq), Jackie Phos Pd G3 (4.8 mg, 4.1 µmol, 15 mol%), Jackie Phos Ligand (3.3 mg, 4.1 µmol, 15 mol%), Cs_2CO_3 (25.5 mg, 80 µmol, 2.8 eq) and freshly dried 3A MS (7 beads) were taken in a microwave vial (5 mL) and flushed with argon. Degassed dry toluene (1 mL) was added, the vial was sealed and heated at 110 °C for 16 h in oil bath. Upon cooling, the resulting mixture was filtered on celite, washed with EtOAc (5 x), and the filtrate was evaporated. The residue was purified by flash column chromatography on silica gel using 0 – 100% ethyl acetate in *n*-hexane to get compound **7**-Ac-^{t}BuO (25 mg, yield: 58%) as an off-white solid.

^{1}H NMR (400 MHz, $CDCl_3$) δ 8.21 (dd, *J* = 8.0, 1.4 Hz, 1H), 8.07 (dd, *J* = 8.0, 0.7 Hz, 1H), 7.73 (t, *J* = 2.3 Hz, 2H), 7.65 (s, 1H), 7.58 (s, 2H), 7.36 (brs, 2H), 7.21 (dd, *J* = 8.8, 2.6 Hz, 2H), 7.08 – 7.01 (m, 2H), 6.74 (dd, *J* = 8.5, 1.0 Hz, 2H), 5.55 – 5.43 (m, 8H), 5.11 – 5.11 (m, 4H), 4.20 – 4.11 (m, 6H), 3.35 (s, 6H, NCH_3), 2.18, 2.18 (s, 6H, COCH_3), 2.06, 2.06 (s, 6H, COCH_3), 2.05, 2.04 (s, 6H, COCH_3), 2.01, 2.01 (s, 6H, COCH_3), 1.84 (s, 3H, C$(CH_3)_2$), 1.72 (s, 3H, C$(CH_3)_2$), 1.55 (s, 9H, C$(CH_3)_3$).

^{13}C NMR (101 MHz, $CDCl_3$) δ 170.5, 170.5 (C), 170.1 (C), 169.9 (C), 169.3 (C), 169.2 (C), 164.1 (C), 156.3 (C), 154.7 (C), 154.5 (C), 148.1 (C), 145.8 (C), 143.9 (C), 143.9 (C), 138.2 (C), 130.7 (CH), 130.5 (CH), 129.2 (C), 128.5 (CH), 126.9 (C), 125.3 (CH), 124.8 (CH), 124.1 (CH), 122.4 (CH), 122.2 (CH),

112.8, 112.7 (CH), 99.0 (CH), 85.6 (C), 82.7 (C), 71.6, 71.5 (CH), 70.6 (CH), 68.3 (CH), 66.9 (CH), 64.1, 64.0 (CH_2), 61.6, 61.6 (CH_2), 38.3 (C), 37.6 CH_3), 34.8 (CH_3), 33.1 (CH_3), 28.0 (CH_3), 20.7, 20.7 (CH_3), 20.6(CH_3), 20.5 (CH_3), 20.5 (CH_3).

HRMS (ESI) calcd for $C_{74}H_{78}N_4O_{32}$ $[M+Na]^+$ 1557.4491, found 1557.4499.

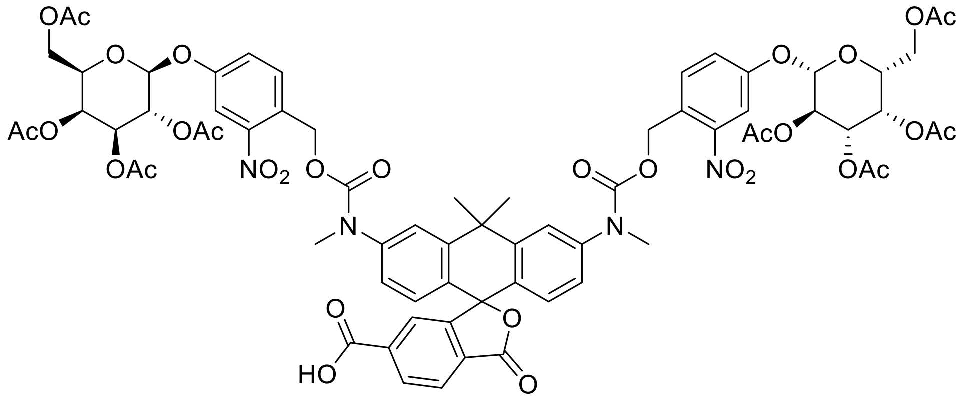

7-Ac-OH

Compound 7-Ac-OH: Trifluoroacetic acid (0.3 mL) was added dropwise to a solution of **7**-Ac-^{t}BuO (10 mg, 6.5 µmol) in CH_2Cl_2 (1.5 mL) at rt. The resulting solution was stirred at room temperature for 16 h. The reaction mixture was co-distilled with toluene (3 x) and then with acetonitrile on rotary evaporator, and was dried under vacuum to get compound **7**-Ac-OH (9 mg, yield: 97%) as a light brown solid.

^{1}H NMR (400 MHz, CD_3CN) δ 8.27 (dd, *J* = 8.0, 1.3 Hz, 1H), 8.12 (dd, *J* = 8.0, 0.8 Hz, 1H), 7.70 (t, *J* = 2.2 Hz, 4H), 7.59 (dd, *J* = 1.4, 0.8 Hz, 1H), 7.44 (brs, 2H), 7.27 (d, *J* = 8.9 Hz, 2H), 7.13 (dt, *J* = 8.7, 1.8 Hz, 2H), 6.82 (d, *J* = 8.6 Hz, 2H), 5.44 (dt, *J* = 3.4, 1.0 Hz, 2H), 5.39 (brs, 4H), 5.37 – 5.30 (m, 4H), 5.23 – 5.15 (m, 2H), 4.33 – 4.26 (m, 2H), 4.13 (dt, *J* = 5.8, 1.7 Hz, 4H), 3.31 (s, 6H), 2.15, 2.14 (s, 6H), 2.02, 2.01 (s, 6H), 1.98, 1.97 (s, 6H), 1.95, 1.95 (s, 6H), 1.74 (s, 3H), 1.66 (s, 3H).

^{13}C NMR (126 MHz, CD_3CN) δ 171.2 (C), 171.1 (C), 170.8, 170.7 (C), 170.7 (C), 170.6 (C), 169.9 (C), 166.3 (C), 157.3 (C), 156.0 (C), 155.4 (C), 149.3 (C), 146.5 (C), 145.2 (C), 137.6 (C), 131.9, 131.9 (CH), 131.8 (CH), 130.1 (C), 129.1 (CH), 129.0 (CH), 127.5 (C), 126.6 (CH), 125.4 (CH), 125.3 (CH), 123.0 (CH), 113.5, 113.5 (CH), 99.4, 99.4 (CH), 86.1 (C), 72.4, 72.4 (CH), 71.3 (CH), 69.2, 69.2 (CH), 68.2 (CH), 64.6, 64.6 (CH_2), 62.5, 62.5 (CH_2), 39.1 (C), 38.0 (CH_3), 34.5 (CH_3), 33.7 (CH_3), 20.9, 20.9 (CH_3), 20.8, 20.8 (CH_3), 20.8 (CH_3), 20.7 (CH_3).

HRMS (ESI) calcd for $C_{70}H_{70}N_4O_{32}$ $[M+Na]^+$ 1501.3865, found 1501.3744.

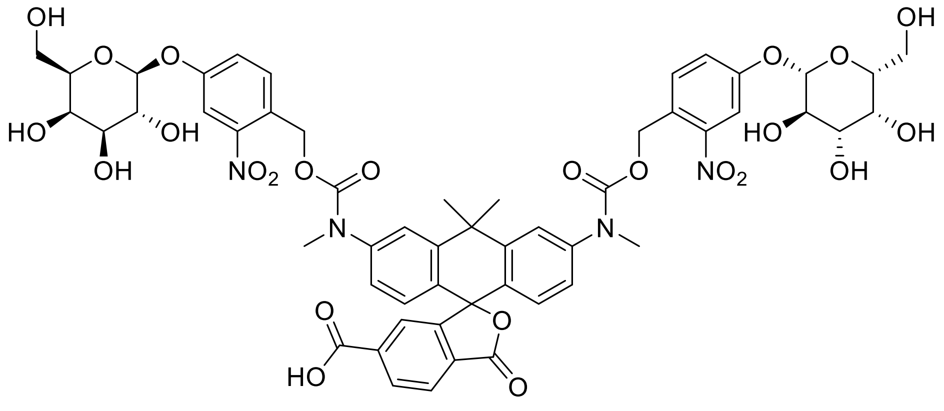

2

β-Gal-*o*-NO$_2$Bn-580CP carboxylic acid (2): Compound **7**-Ac-OH (6 mg, 3.4 µmol), LiOH*H$_2$O (2.4 mg, 56 µmol), MeOH (0.7 ml) and H$_2$O (0.7 ml) were taken in a 5 mL round-bottom-flask and stirred at rt for 1.5 h. The reaction mixture was acidified with aq 1M HCl and concentrated under reduced pressure. The crude mixture was subjected to preparative HPLC (16 mm column C18, A:B 70:30 → 0:100 over 25 min, A – water+ 0.1% TFA, B – acetonitrile + 0.1% TFA) to get compound **2** (3 mg, yield 70%) after lyophilization as an off-white solid. Analytical HPLC purity: 95%.

1H-NMR:

^{1}H NMR (400 MHz, CD$_3$OD) δ 8.30 (dd, *J* = 8.0, 1.3 Hz, 1H), 8.16 – 8.11 (m, 1H), 7.79 – 7.66 (m, 4H), 7.61 (s, 1H), 7.38 (d, *J* = 13.0 Hz, 4H), 7.14 (dd, *J* = 8.6, 2.1 Hz, 2H), 6.82 (d, *J* = 8.6 Hz, 2H), 5.41 (s, 4H), 4.96 (dd, *J* = 9.0, 7.7 Hz, 2H), 3.90 (t, *J* = 3.0 Hz, 2H), 3.85 – 3.77 (m, 2H), 3.76 – 3.67 (m, 6H), 3.60 (ddd, *J* = 9.7, 3.4, 1.9 Hz, 2H), 3.34 (s, 6H), 1.75 (s, 3H), 1.67 (s, 3H).

HRMS (ESI) calcd for $C_{54}H_{54}N_4O_{24}$ $[M+Na]^+$ 1165.3020, found 1165.3039.

Scheme S6 (Synthesis compound 7-H-HT)

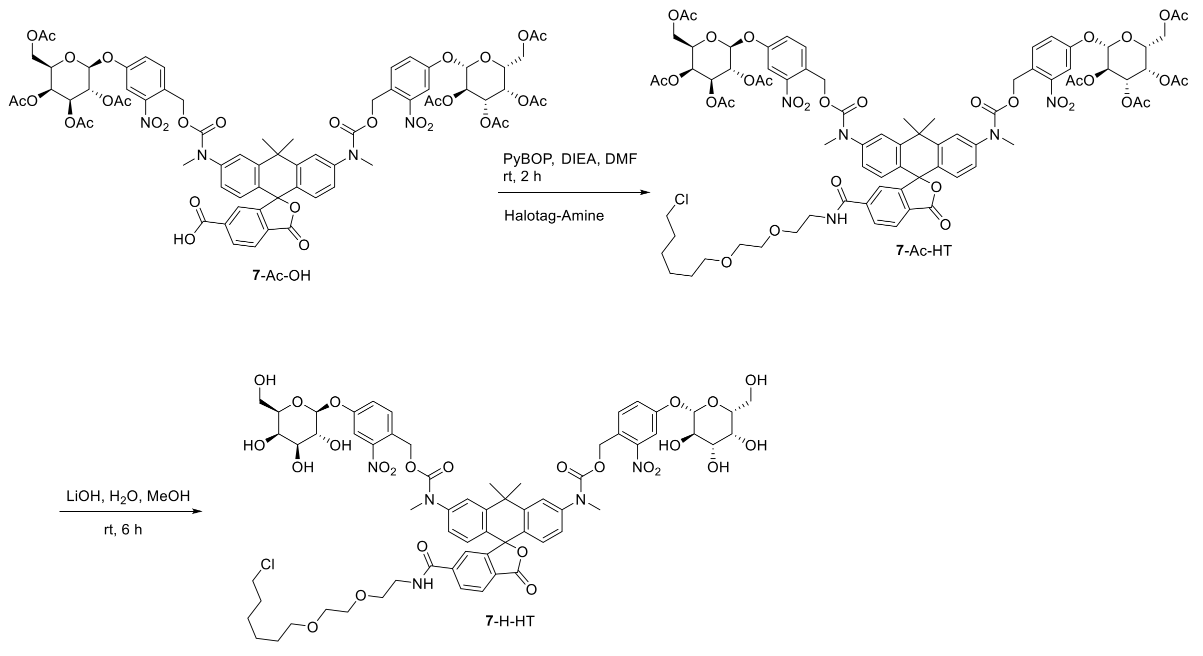

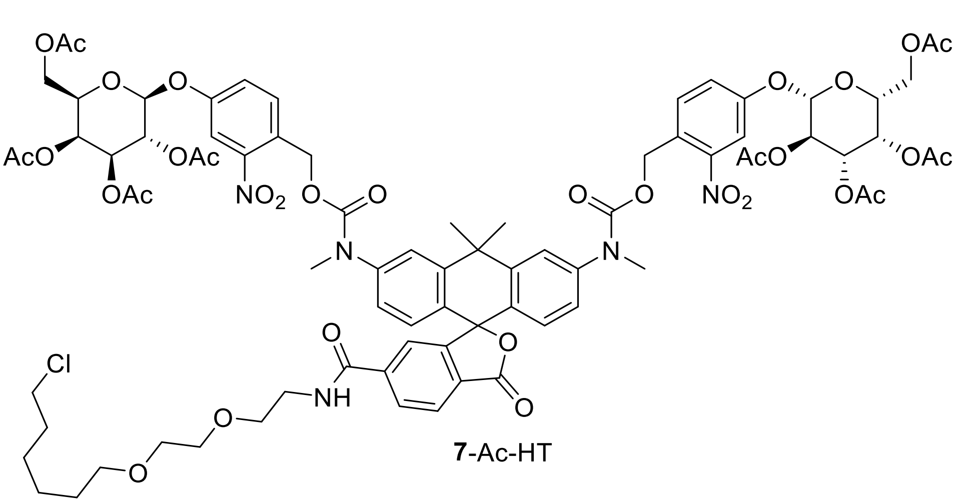

7-Ac-HT

Compound 7-Ac-HT: Compound **7**-Ac-OH (10 mg, 7 µmol, 1 eq), HaloTag-Amine (2.3 mg, 10 µmol, 1.5 eq) and *N,N*-diisopropylethylamine (5 µL, 27 µmol, 4 eq) were taken in dry DMF (1.5 mL) and then PyBOP (4.2 mg, 8 µmol, 1.2 eq) was added at rt, reaction mixture was stirred at rt for 2 h. The reaction mixture was concentrated under vacuum and the residue was purified by silica gel chromatography (0–10% MeOH/CH_2Cl_2, linear gradient) to provide compound **7**-Ac-HT (10 mg, yield: 88%) as a white solid.

^{1}H NMR (400 MHz, CD_3CN) δ 8.13 – 8.00 (m, 2H), 7.71 (dd, *J* = 2.6, 1.8 Hz, 4H), 7.46 (d, *J* = 8.3 Hz, 2H), 7.41 – 7.37 (m, 1H), 7.33 – 7.18 (m, 3H), 7.14 (ddd, *J* = 8.5, 2.3, 0.9 Hz, 2H), 6.81 (d, *J* = 8.6 Hz, 2H), 5.44 (td, *J* = 3.7, 1.1 Hz, 2H), 5.41 – 5.29 (m, 8H), 5.22 – 5.15 (m, 2H), 4.31 (tdd, *J* = 6.2, 2.9, 1.1 Hz, 2H), 4.16 – 4.10 (m, 4H), 3.58 – 3.40 (m, 10H), 3.32 (s, 6H), 3.30 (t, *J* = 8 Hz, 2H), 2.14 (s, 6H), 2.02, 2.01 (s, 6H), 1.98, 1.97 (s, 6H), 1.96 (s, 6H), 1.76 (s, 3H), 1.73 – 1.66 (m, 2H), 1.66 (s, 3H), 1.45 – 1.37 (m, 2H), 1.36 – 1.23 (m, 4H).

^{13}C NMR (126 MHz, CD_3CN) δ 171.1 (C), 171.1, 171.1 (C), 170.7 (C), 170.4, 170.4 (C), 170.1 (C), 166.4 (C), 157.3, 157.3 (C), 156.0 (C), 155.4 (C), 149.3 (C), 146.6 (C), 145.2 (C), 142.4 (C), 131.9, 131.9 (CH), 129.5 (CH), 129.2, 129.1 (CH), 129.1 (C), 128.7 (C), 127.5 (C), 126.4 (CH), 125.4 (CH), 125.3 (CH), 123.1 (CH), 122.9 (CH), 113.6 (CH), 99.4 (CH), 86.1 (C), 72.4, 72.4 (CH), 71.5 (CH_2), 71.3, 71.32(CH), 70.8 (CH_2), 70.7 (CH_2), 69.8 (CH_2), 69.2 (CH), 68.2, 68.2 (CH), 64.6 (CH_2), 62.5, 62.5 (CH_2), 46.1 (CH_2), 40.6 (CH_2), 39.1 (C), 38.0 (CH_3), 34.6 (CH_3), 33.6 (CH_3), 33.2 (CH_2), 30.1 (CH_2), 27.3 (CH_2), 26.0 (CH_2), 20.9, 20.8 (CH_3), 20.8 (CH_3), 20.8 (CH_3), 20.7 (CH_3).

HRMS (ESI) calcd for $C_{80}H_{90}ClN_5O_{33}$ [M+Na]$^+$ 1706.5099, found 1706.5070.

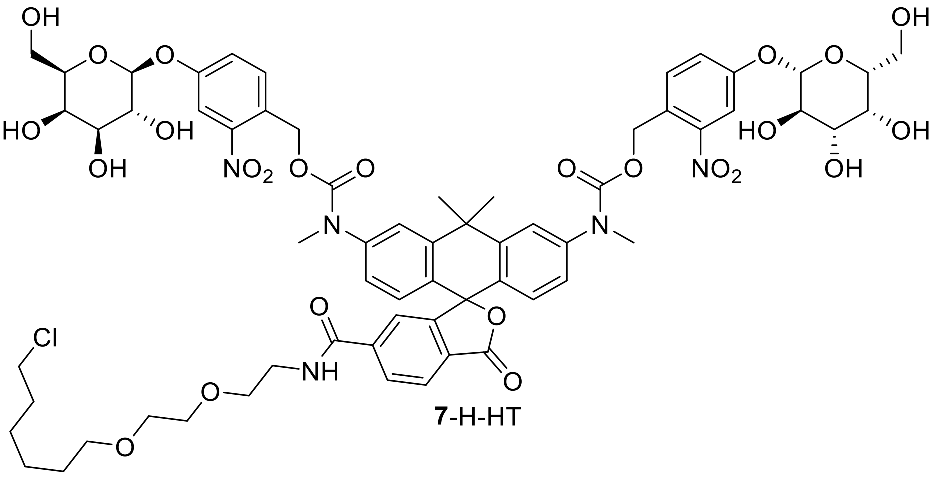

7-H-HT

β-Gal-*o*-NO_2Bn-580CP-HT (7-H-HT): A mixture of compound **7**-Ac-HT (5 mg, 3 µmol), LiOH*H_2O (2.4 mg, 57 µmol), MeOH (0.8 ml) and H_2O (0.8 ml) was stirred at rt for 6 h. The reaction mixture was acidified with 1N HCl, extracted with EtOAc (6 x), dried (Na_2SO_4) and concentrated. Crude mixture was purified by reverse phase flash chromatography (Interchim, 16 mm, column C18, A: B 20:80 → 0:100 over 25 min, A – acetonitrile, B – water + 0.1% TFA) to get compound **7**-H-HT (3 mg, yield: 64%) as an off-white solid. Analytical HPLC purity: 93.2%.

^{1}H NMR (400 MHz, DMSO-*d*$_6$) δ 8.79 (t, *J* = 5.6 Hz, 1H), 8.20 – 8.08 (m, 2H), 7.78 (d, *J* = 2.3 Hz, 2H), 7.70 – 7.62 (m, 2H), 7.51 (d, *J* = 6.8 Hz, 3H), 7.36 (dd, *J* = 8.6, 2.6 Hz, 2H), 7.16 (d, *J* = 8.6 Hz, 2H), 6.68 (d, *J* = 8.6 Hz, 2H), 5.34 (s, 4H), 5.22 (d, *J* = 5.0 Hz, 2H), 4.93 (dd, *J* = 7.6, 3.5 Hz, 2H), 4.88 (d, *J* = 5.7 Hz, 2H), 4.63 (td, *J* = 5.6, 2.2 Hz, 2H), 4.53 (d, *J* = 4.6 Hz, 2H), 3.70 – 3.68 (m, 2H), 3.63 – 3.51 (m, 8H), 3.47 – 3.36 (m, 6H), 1.76 (s, 3H), 1.67 – 1.60 (m, 2H), 1.63 (s, 3H), 1.41 – 1.34 (m, 2H), 1.32 – 1.19 (m, 4H).

HRMS (ESI) calcd for $C_{64}H_{74}ClN_5O_{25}$ [M+Na]$^+$ 1370.4254, found 1370.4259.

Supplementary references

[S1]

Kim, E.-J.; Kumar, R.; Sharma, A.; Yoon, B.; Kim, H. M.; Lee, H.; Hong, K. S.; Kim, J. S. In vivo imaging of b-galactosidase stimulated activity in hepatocellular carcinoma using ligand-targeted fluorescent probe. *Biomaterials* **2017**, *122*, 83−90.

[S2]

Aktalay, A.; Khan, T. A.; Bossi, M. L.; Belov, V. N.; Hell, S. W. Photoactivatable Carbo- and Silicon-Rhodamines and Their Application in MINFLUX Nanoscopy. *Angew. Chem. Int. Ed.* **2023**, *62*, 41, e202302781; *Angew. Chem.* **2023**, *135*, 41, e202302781.

^{1}H-NMR and ^{13}C-NMR Spectra

^{1}H NMR (400 MHz, $CDCl_3$) of compound **4**

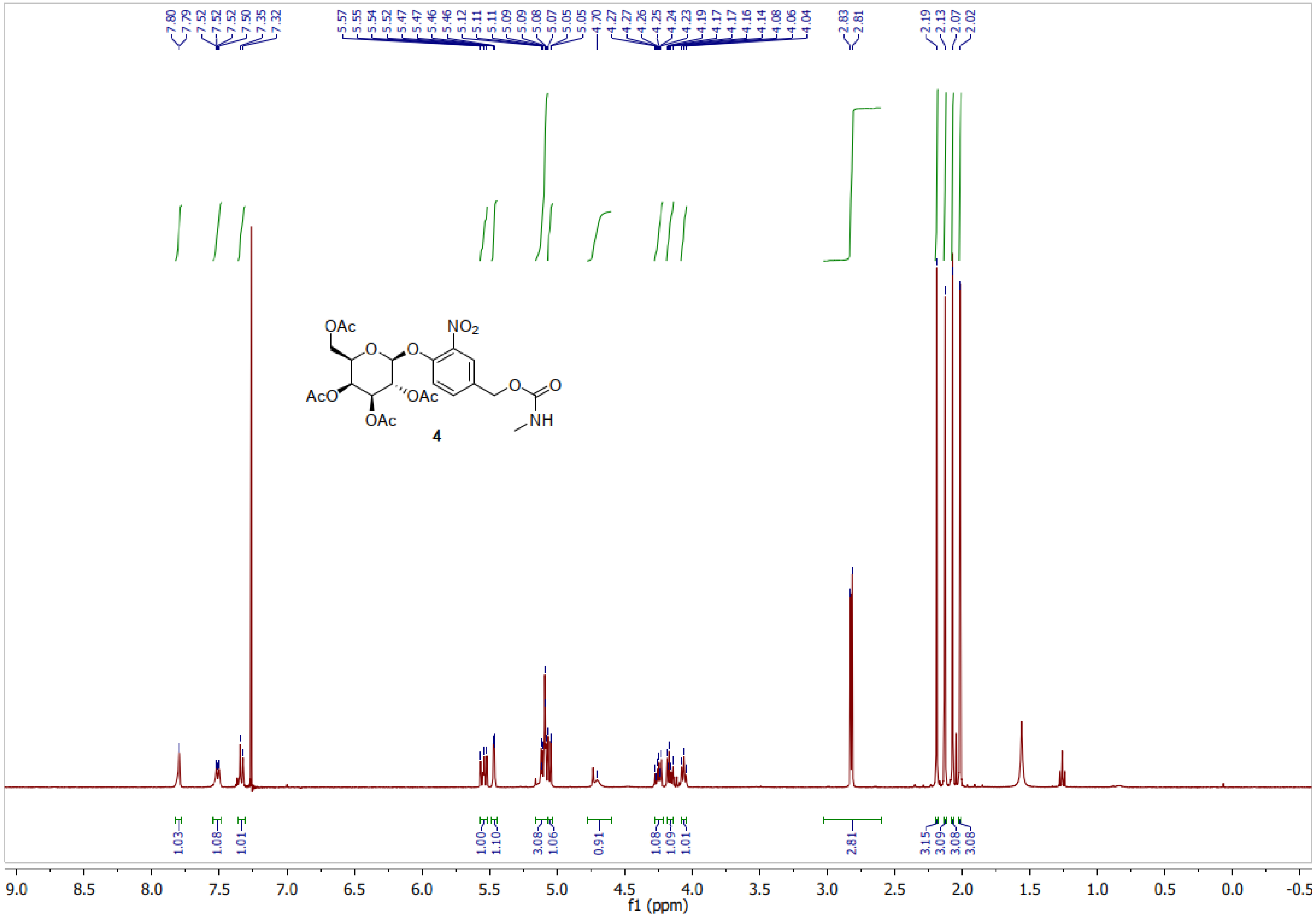

^{13}C NMR (101 MHz, $CDCl_3$) of compound **4**

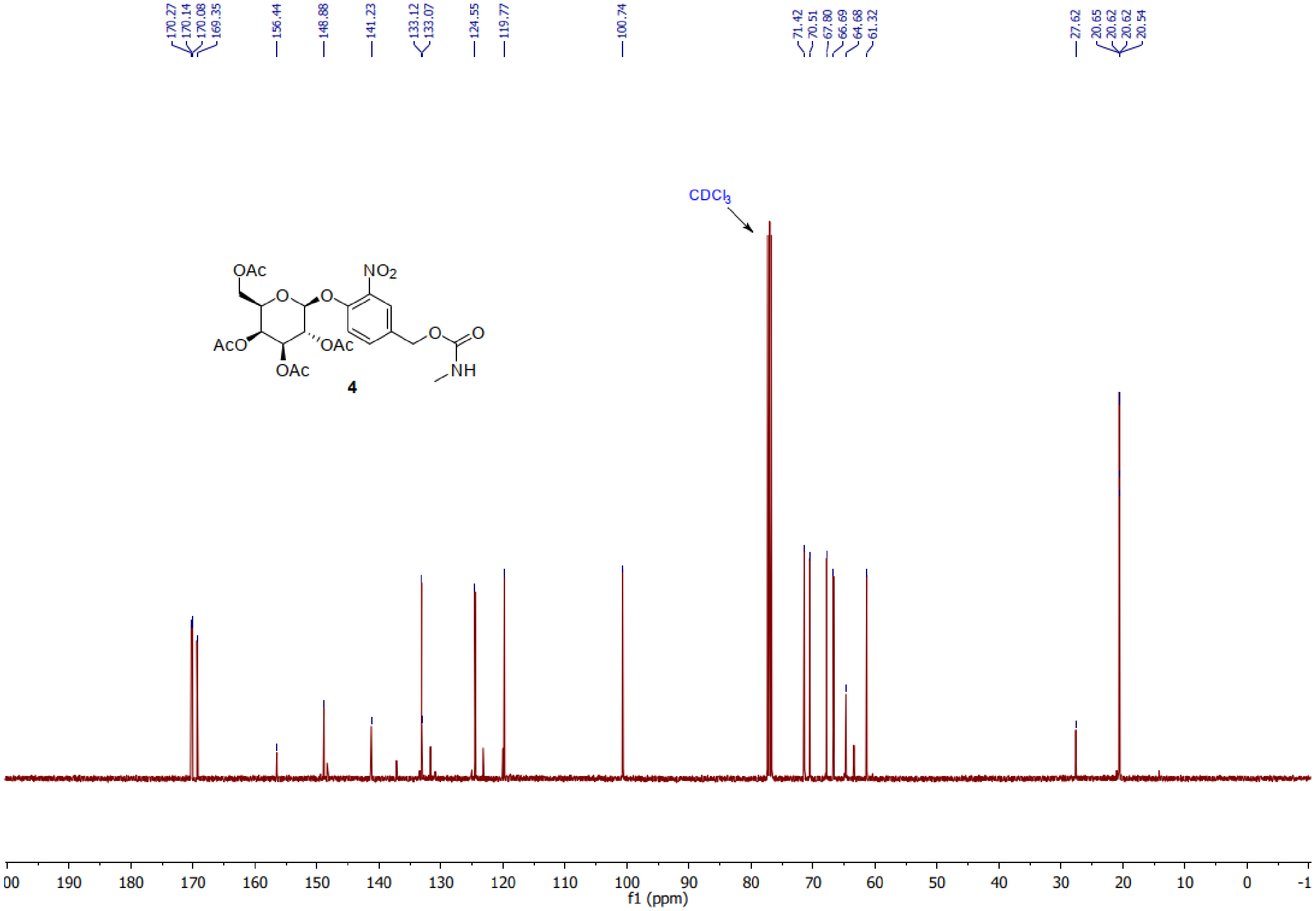

^{1}H NMR (400 MHz, CDCl3) of compound **5-**Ac-*t*BuO

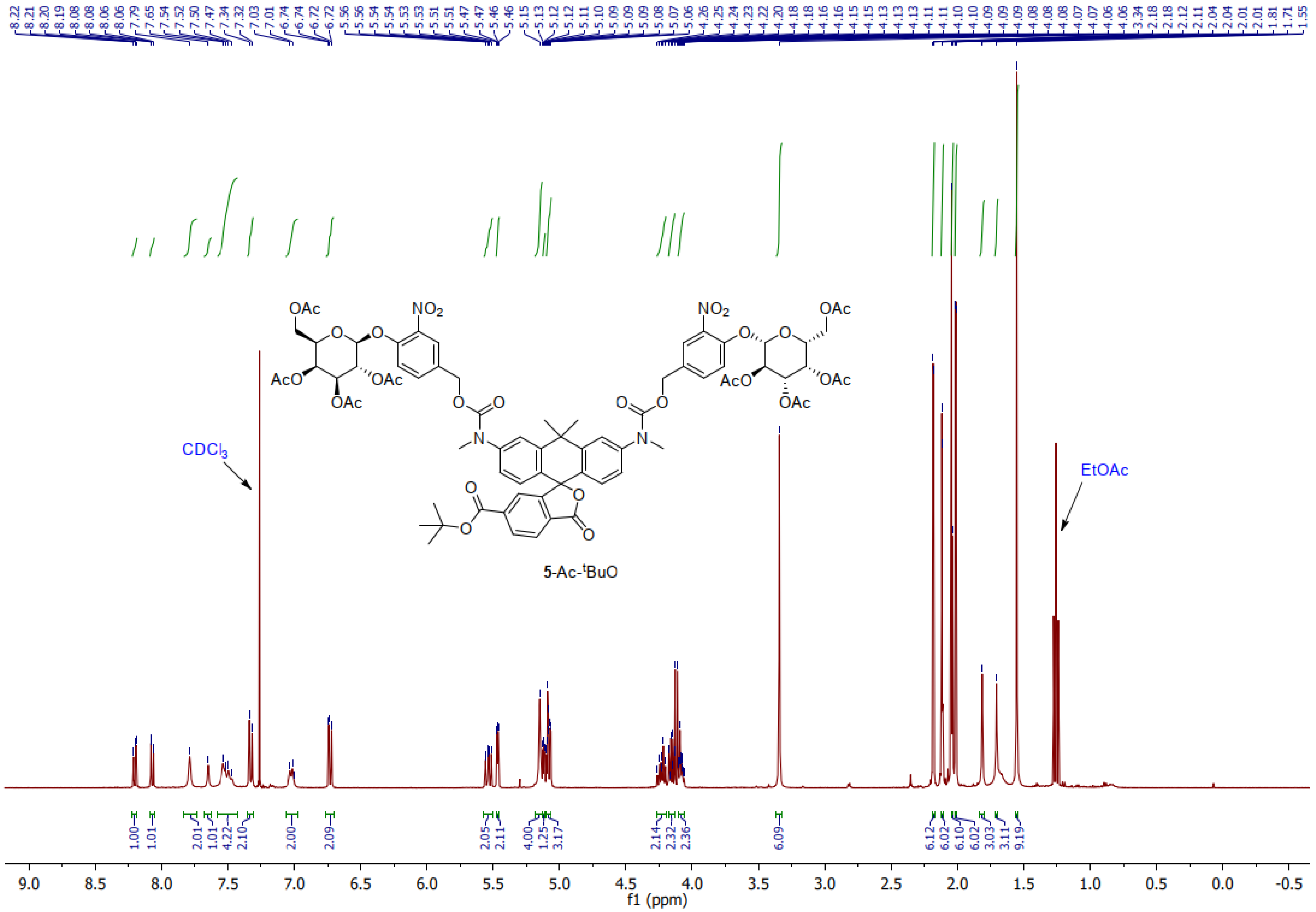

^{13}C NMR (126 MHz, $CDCl_3$) of **5**-Ac-^{t}BuO

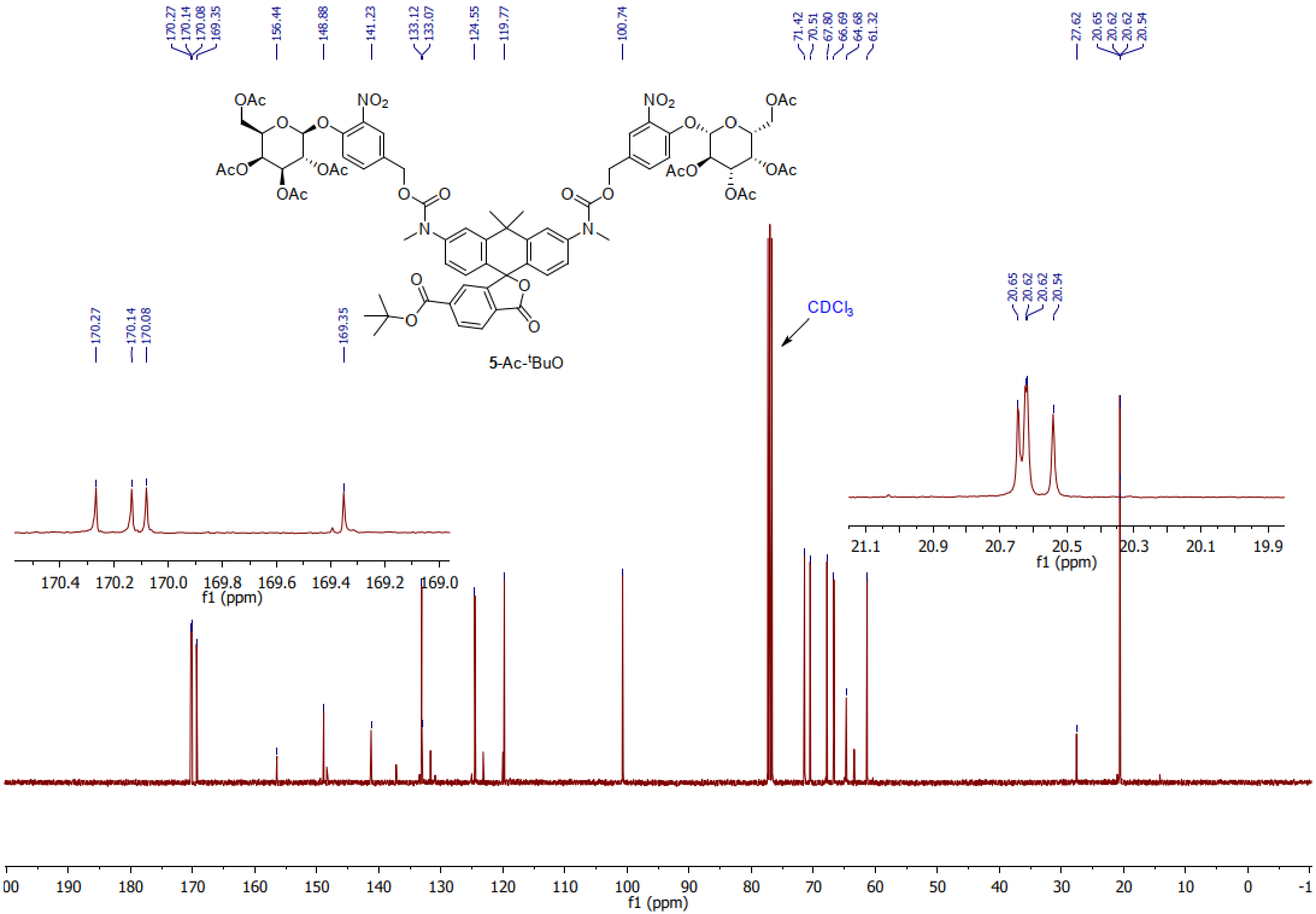

^{1}H NMR (400 MHz, CD_3CN) of compound **5**-Ac-OH

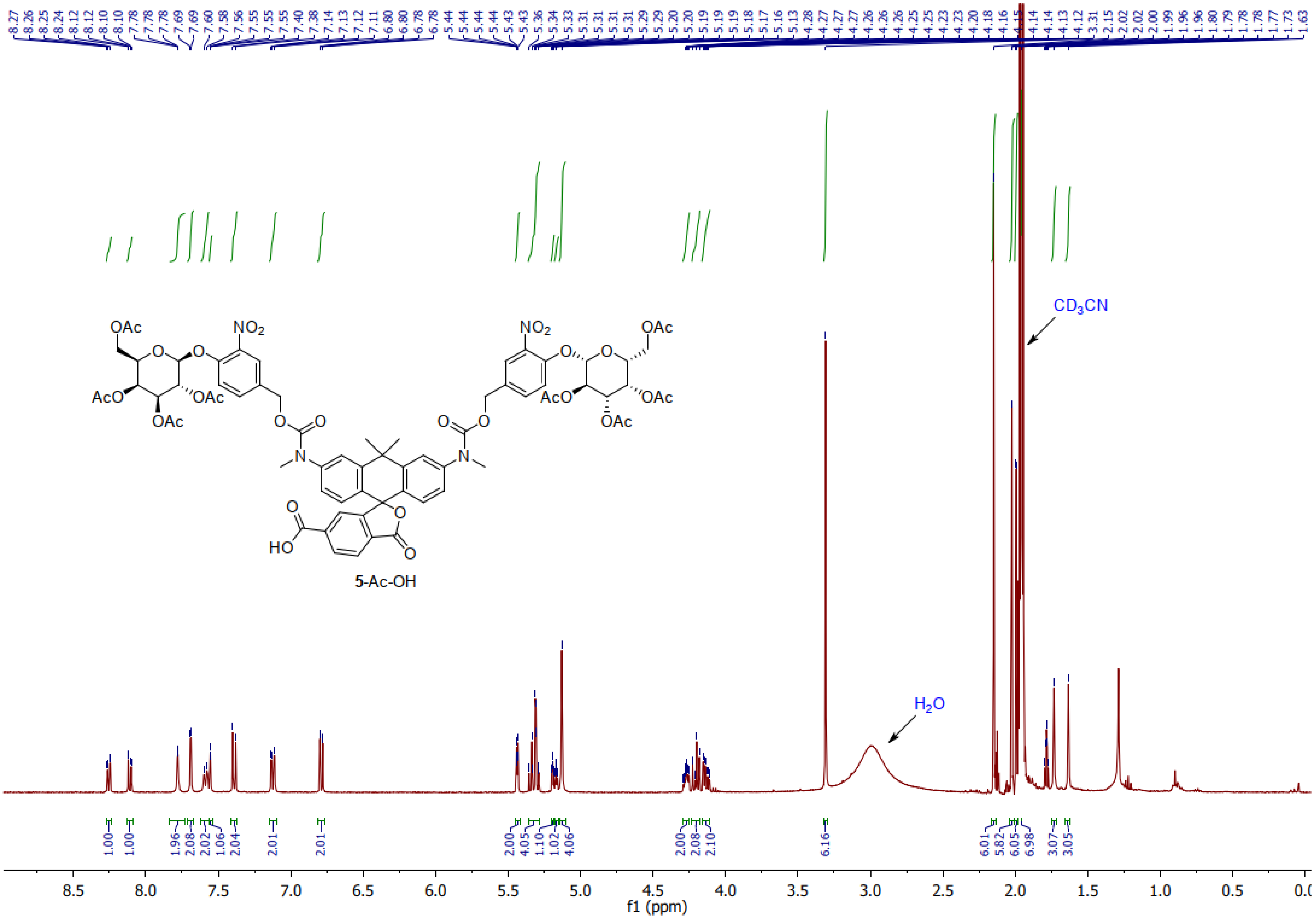

^{13}C NMR (126 MHz, CD_3CN) of compound **5**-Ac-OH

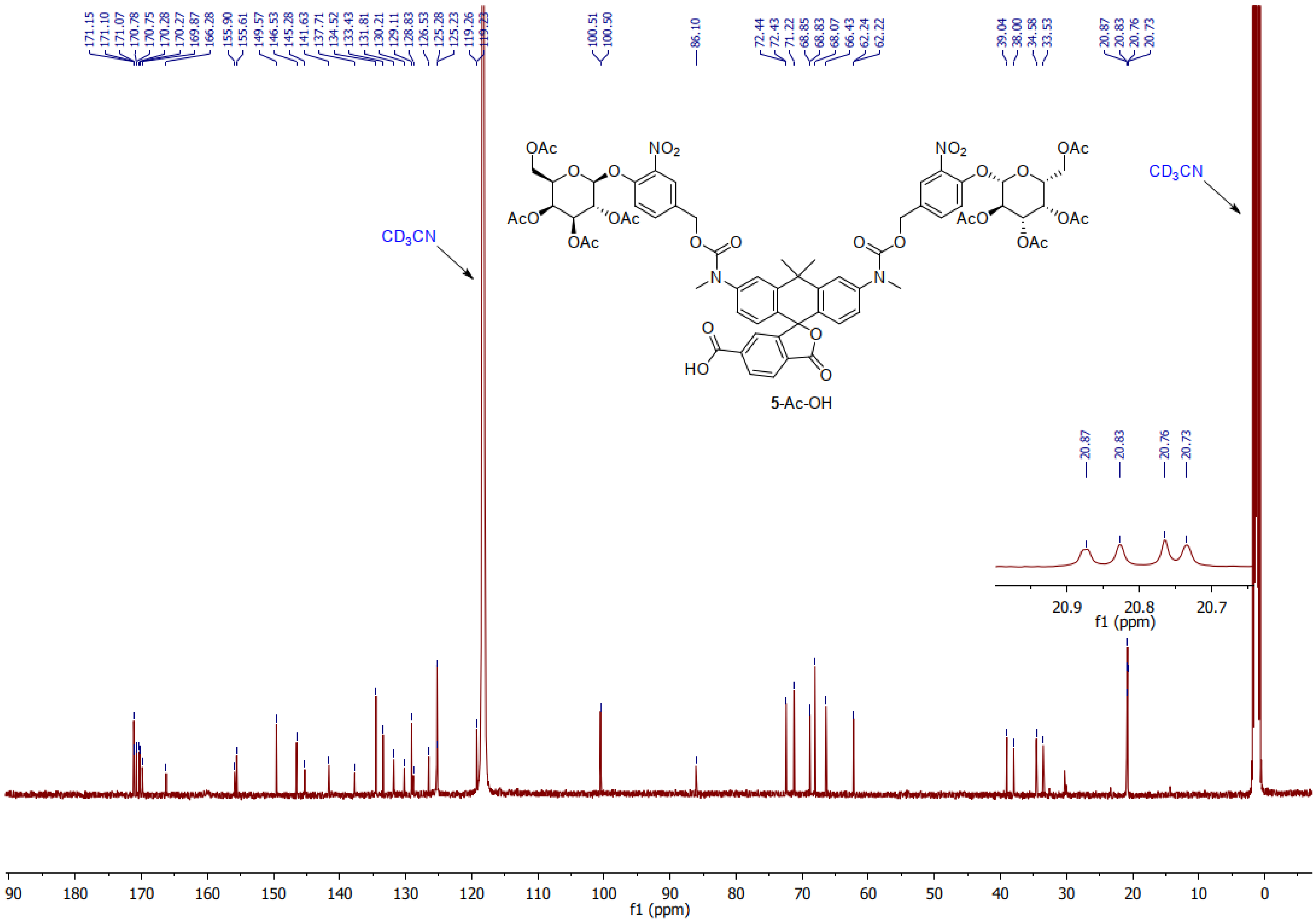

^{1}H NMR (400 MHz, CD_3OD) of compound **1**

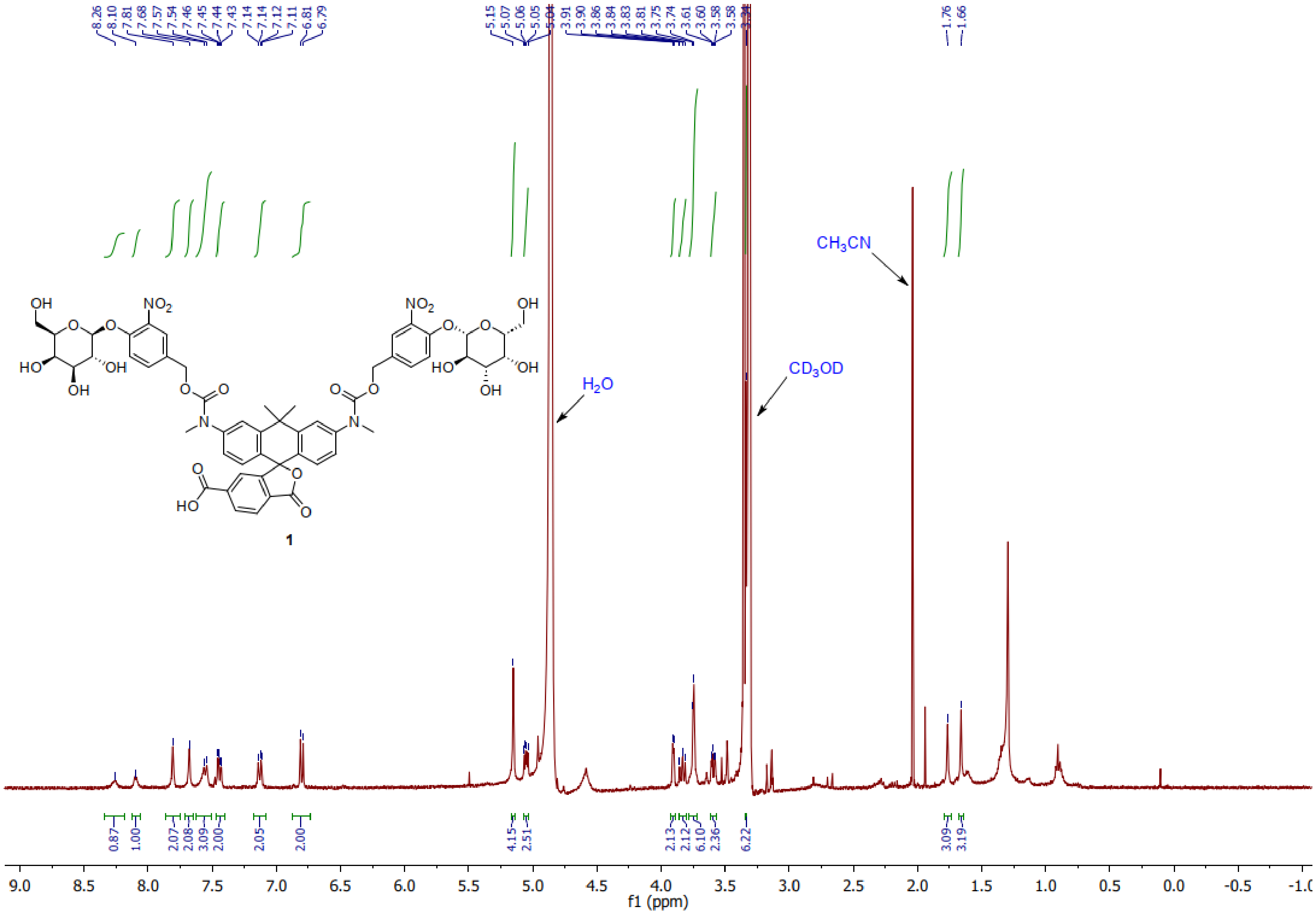

Analytical HPLC of compound **1**

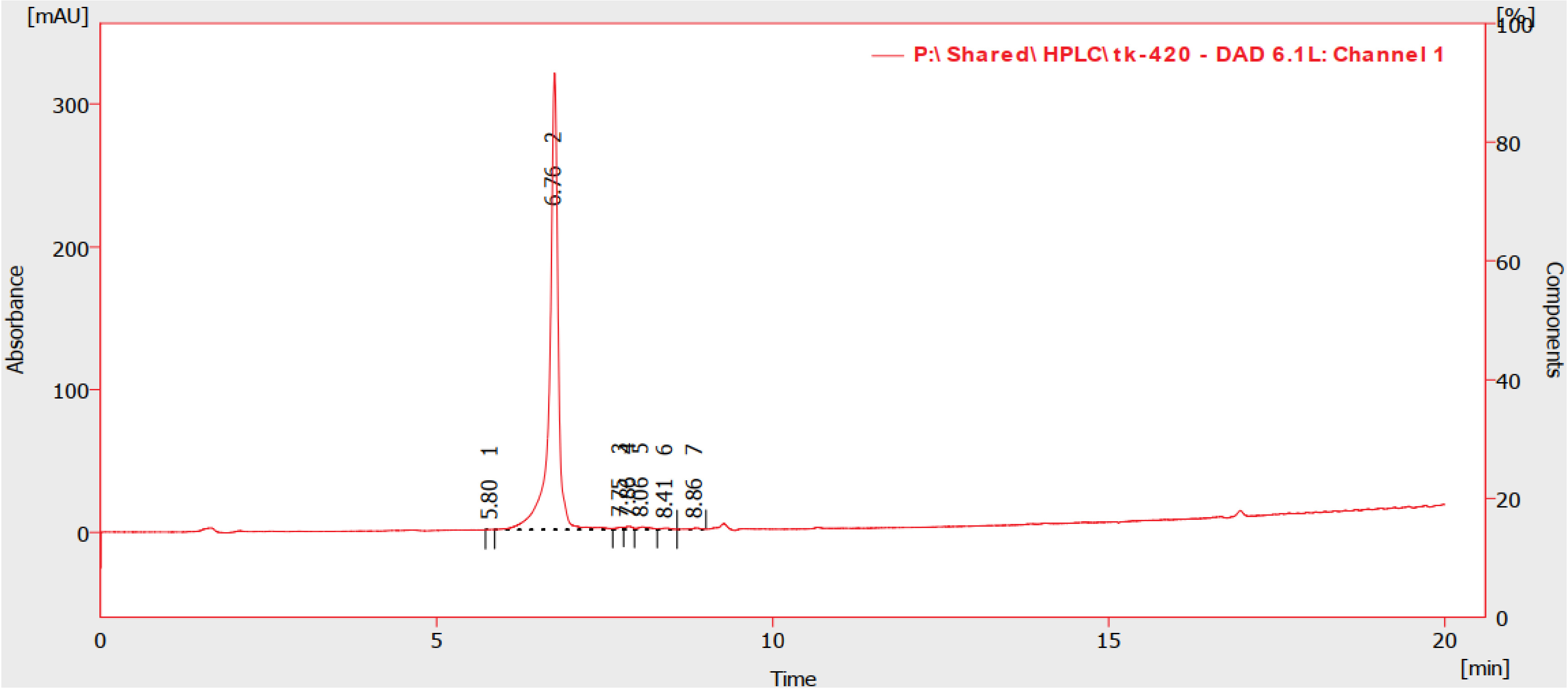

^{1}H NMR (400 MHz, CD_3CN) of compound **5**-Ac-HT

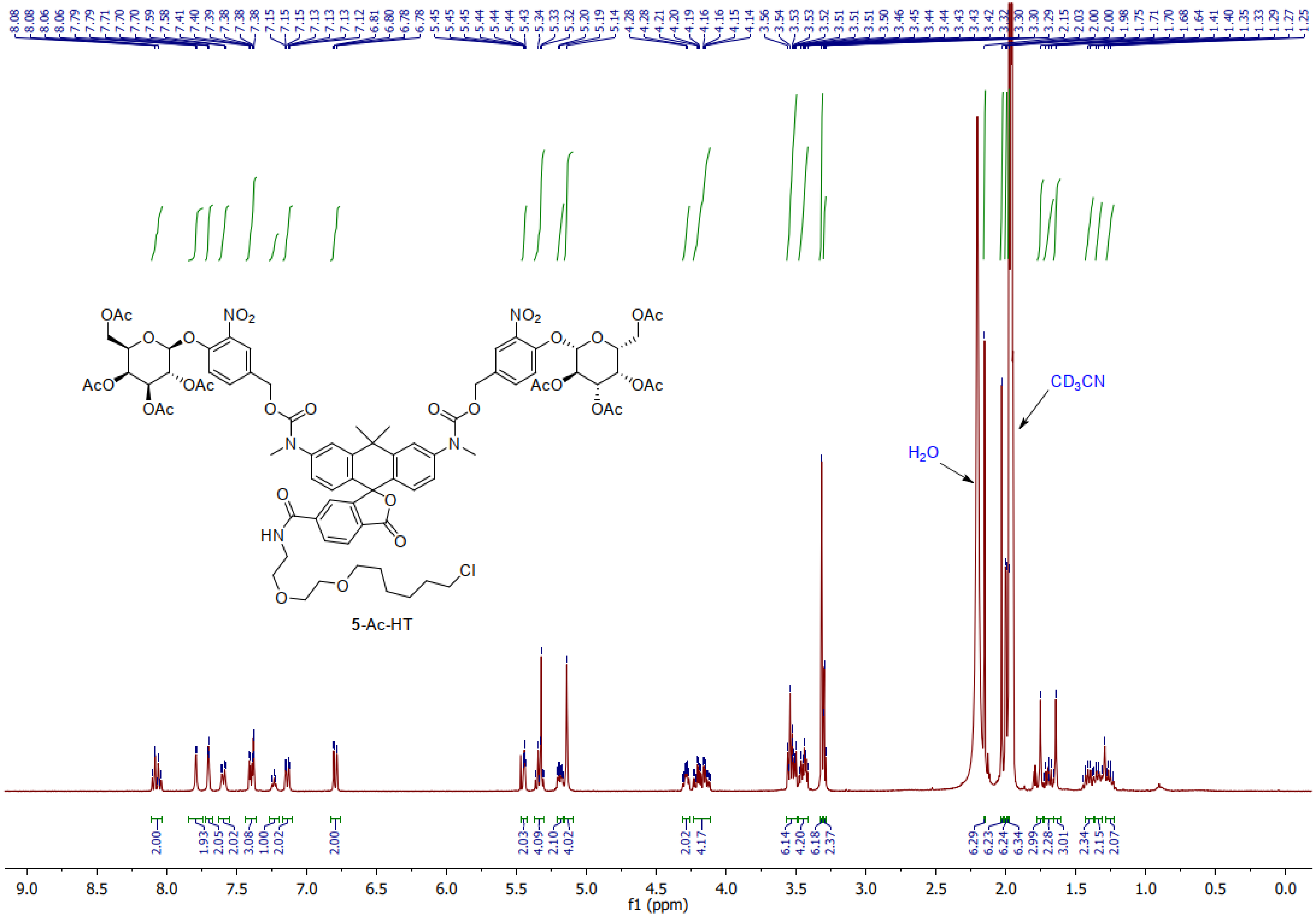

^{13}C NMR (126 MHz, CD_3CN) of compound **5**-Ac-HT

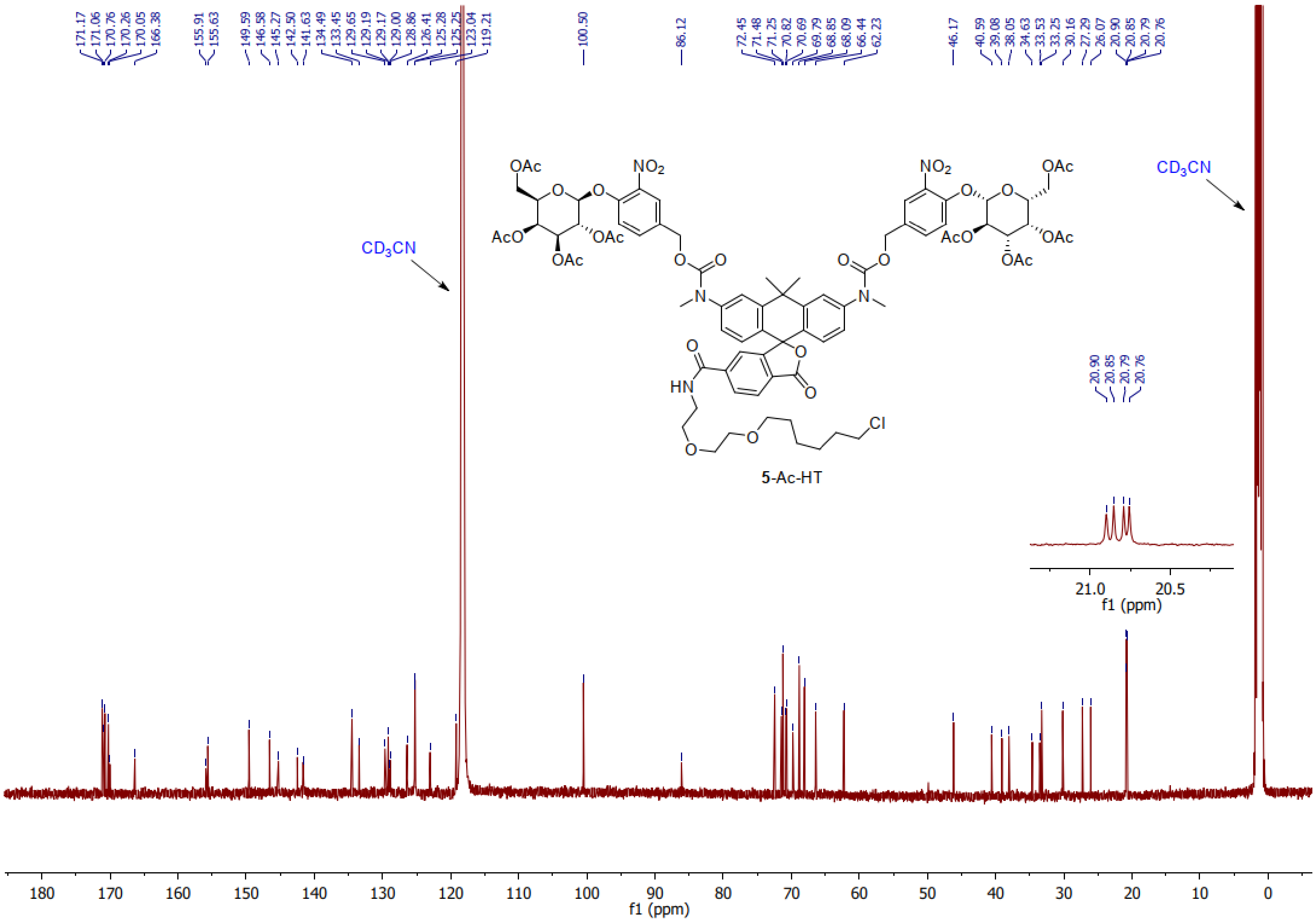

^{1}H NMR (400 MHz, DMSO-d_6) of compound **5**-H-HT

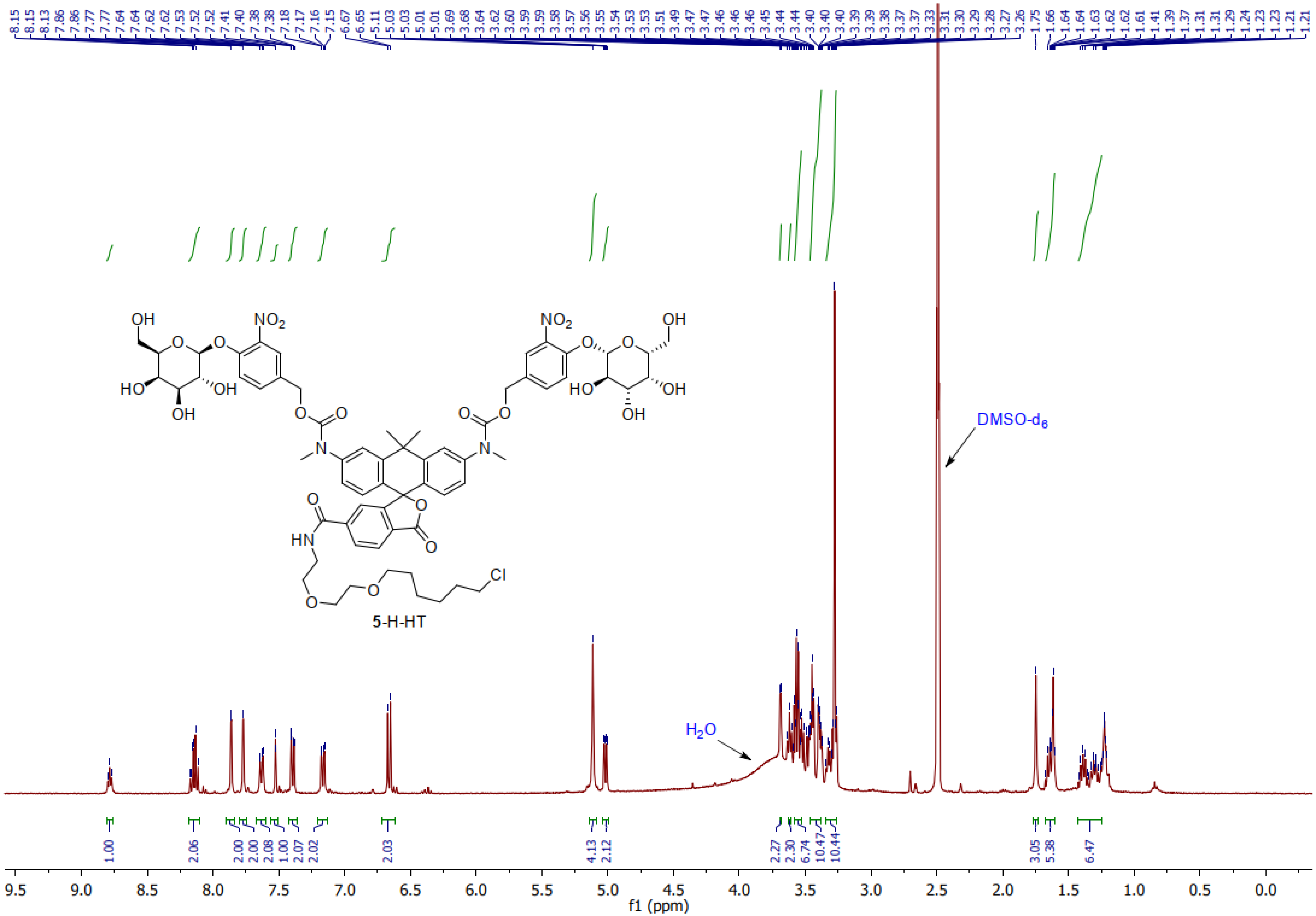

Analytical HPLC of compound **5**-H-HT

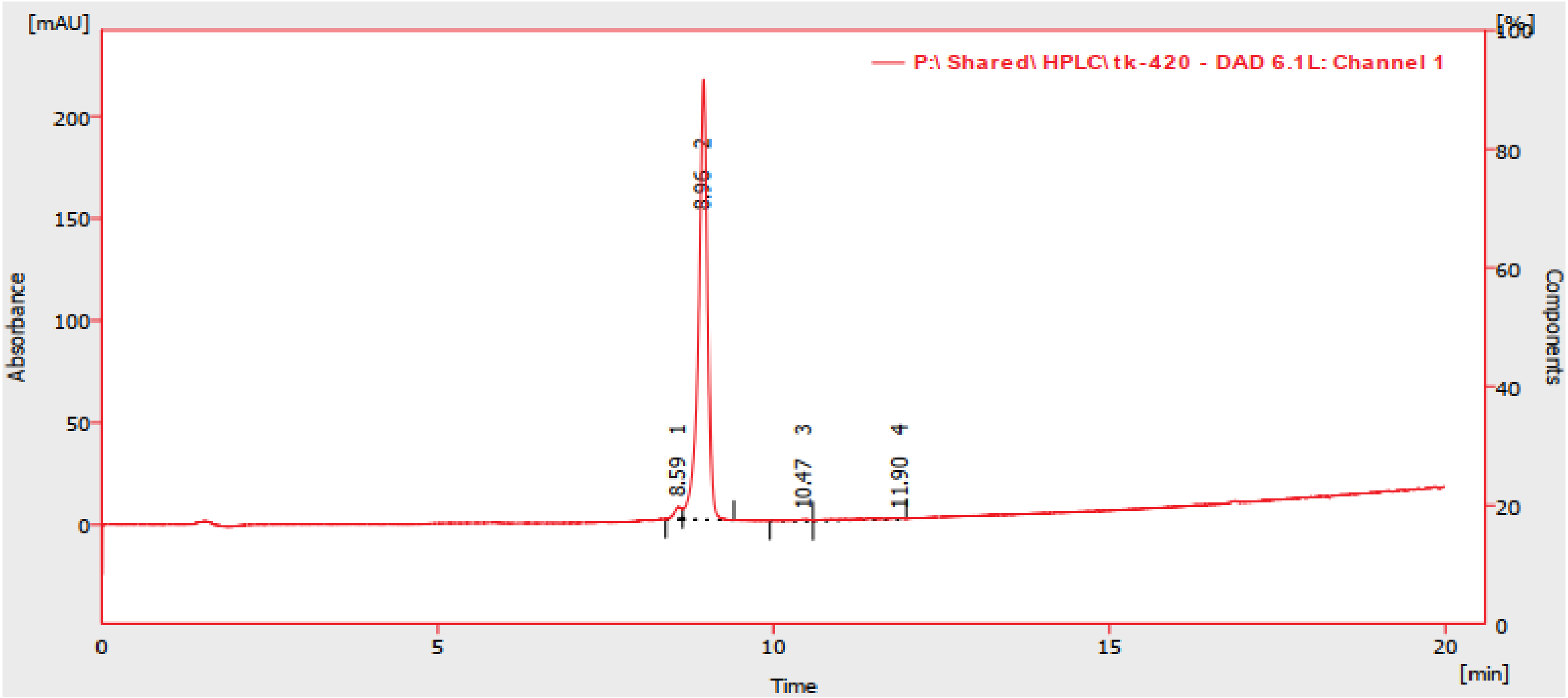

^{1}H NMR (400 MHz, $CDCl_3$) of compound **S7**

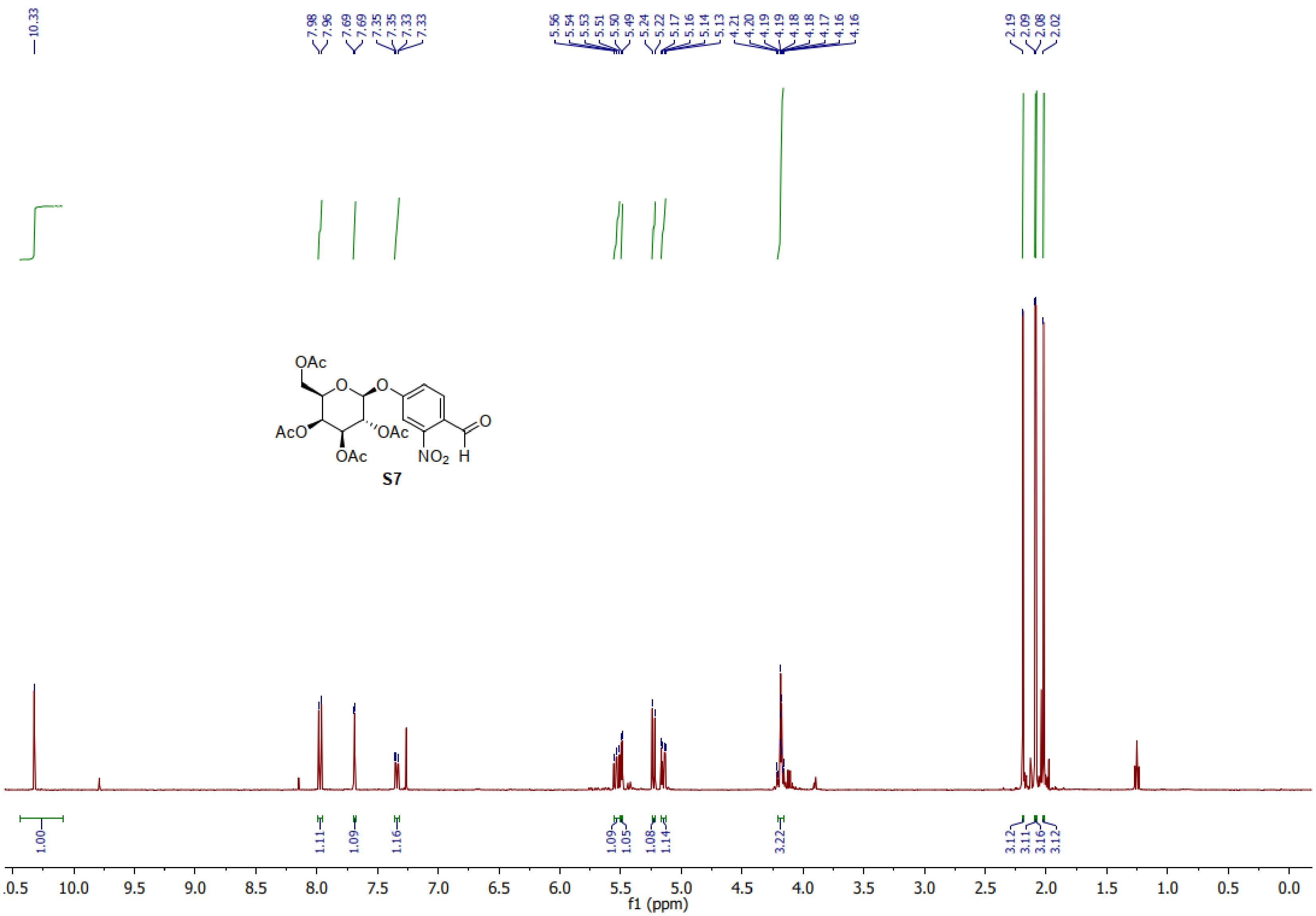

^{13}C NMR (101 MHz, $CDCl_3$) of compound **S7**

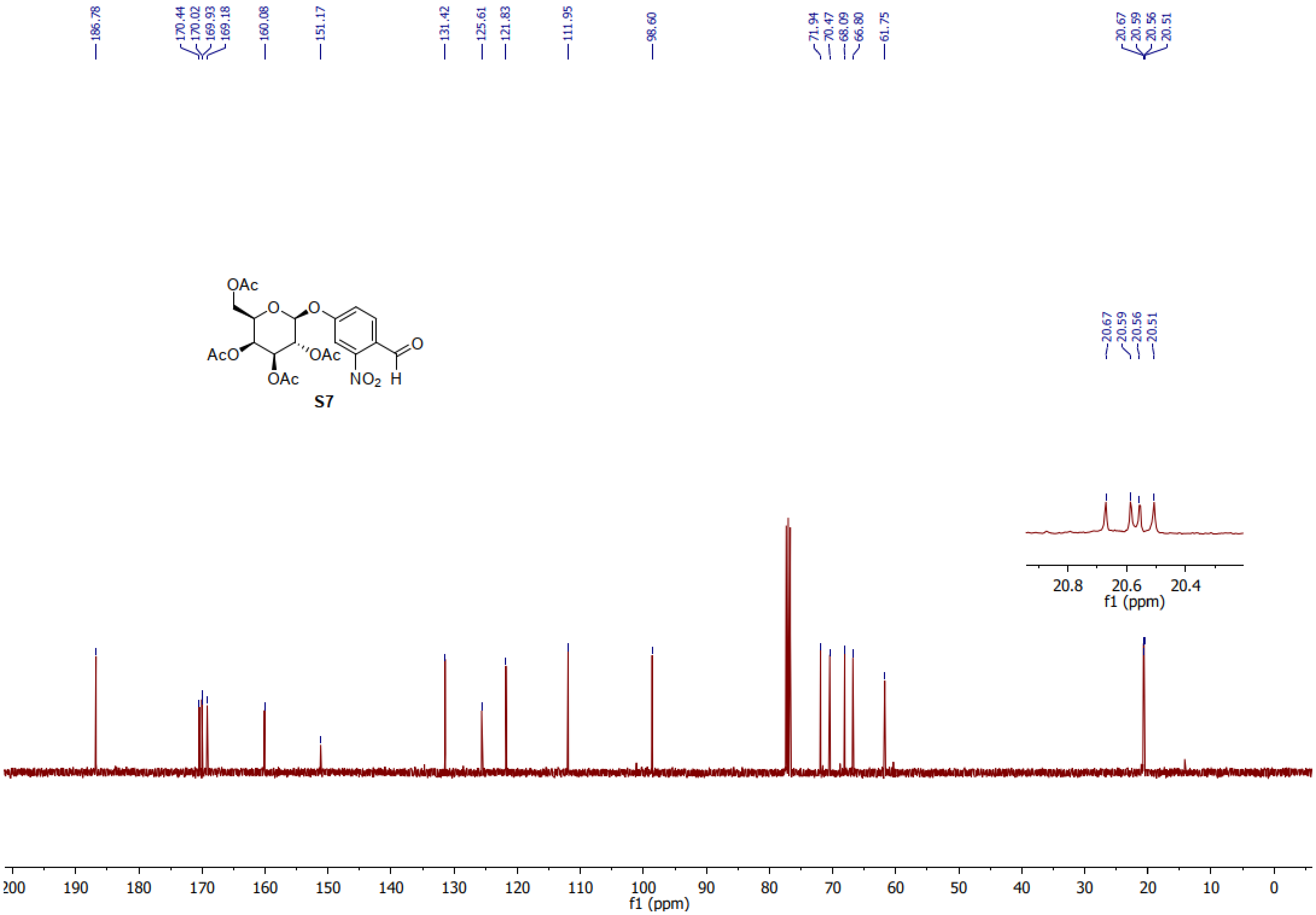

^{1}H NMR (400 MHz, $CDCl_3$) of compound **S8**

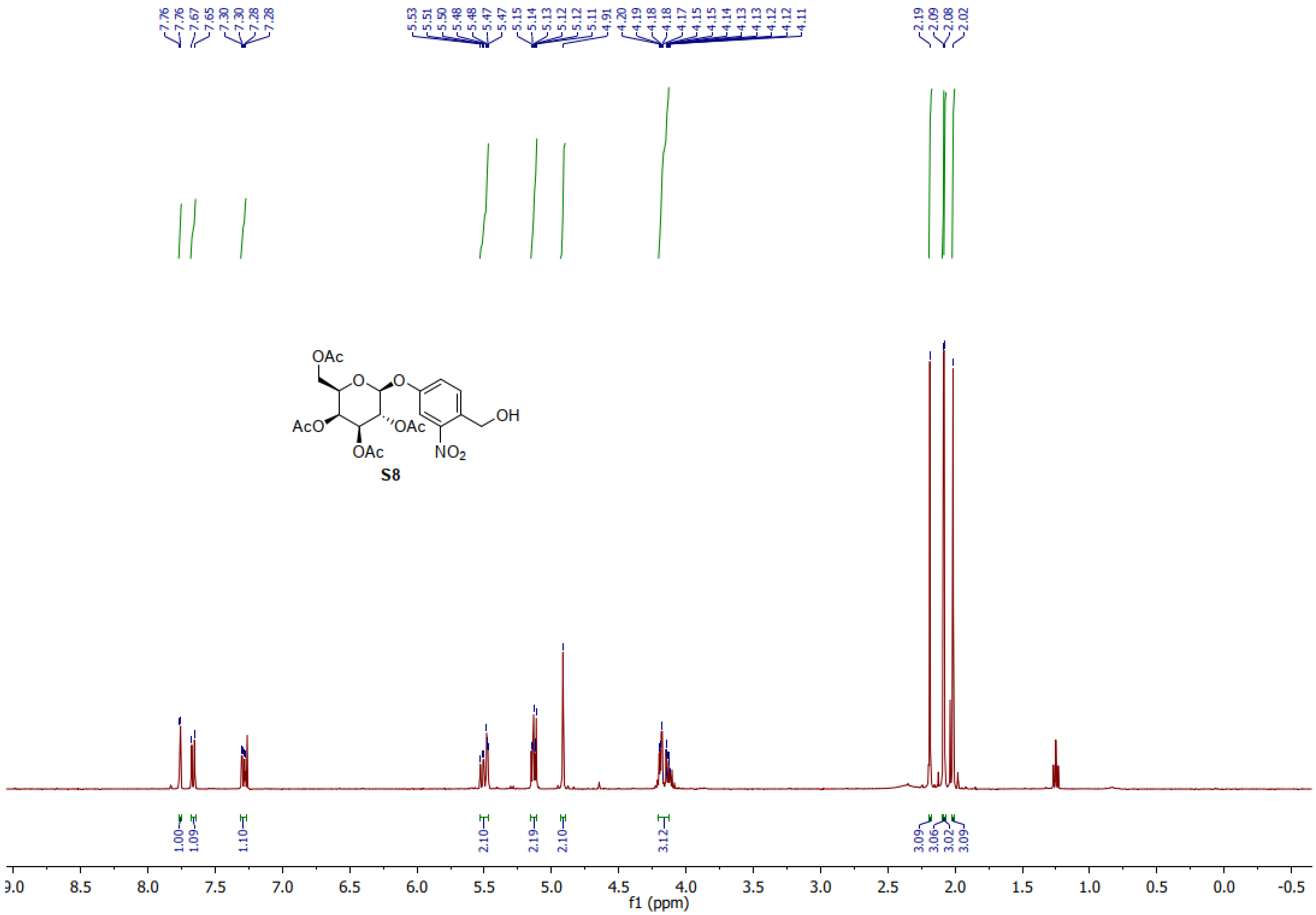

^{13}C NMR (101 MHz, $CDCl_3$) of compound **S8**

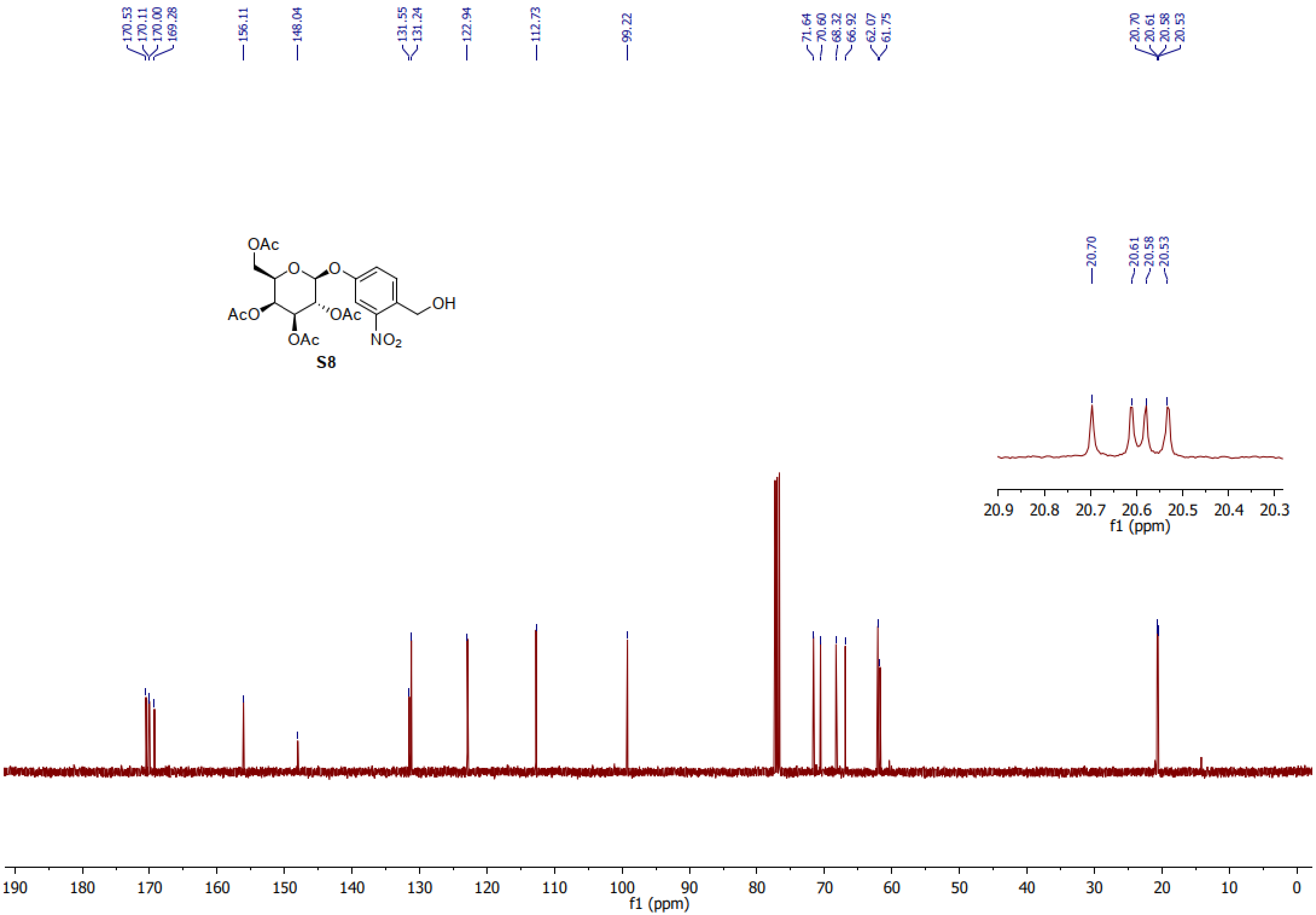

^{1}H NMR (400 MHz, $CDCl_3$) of compound **6**

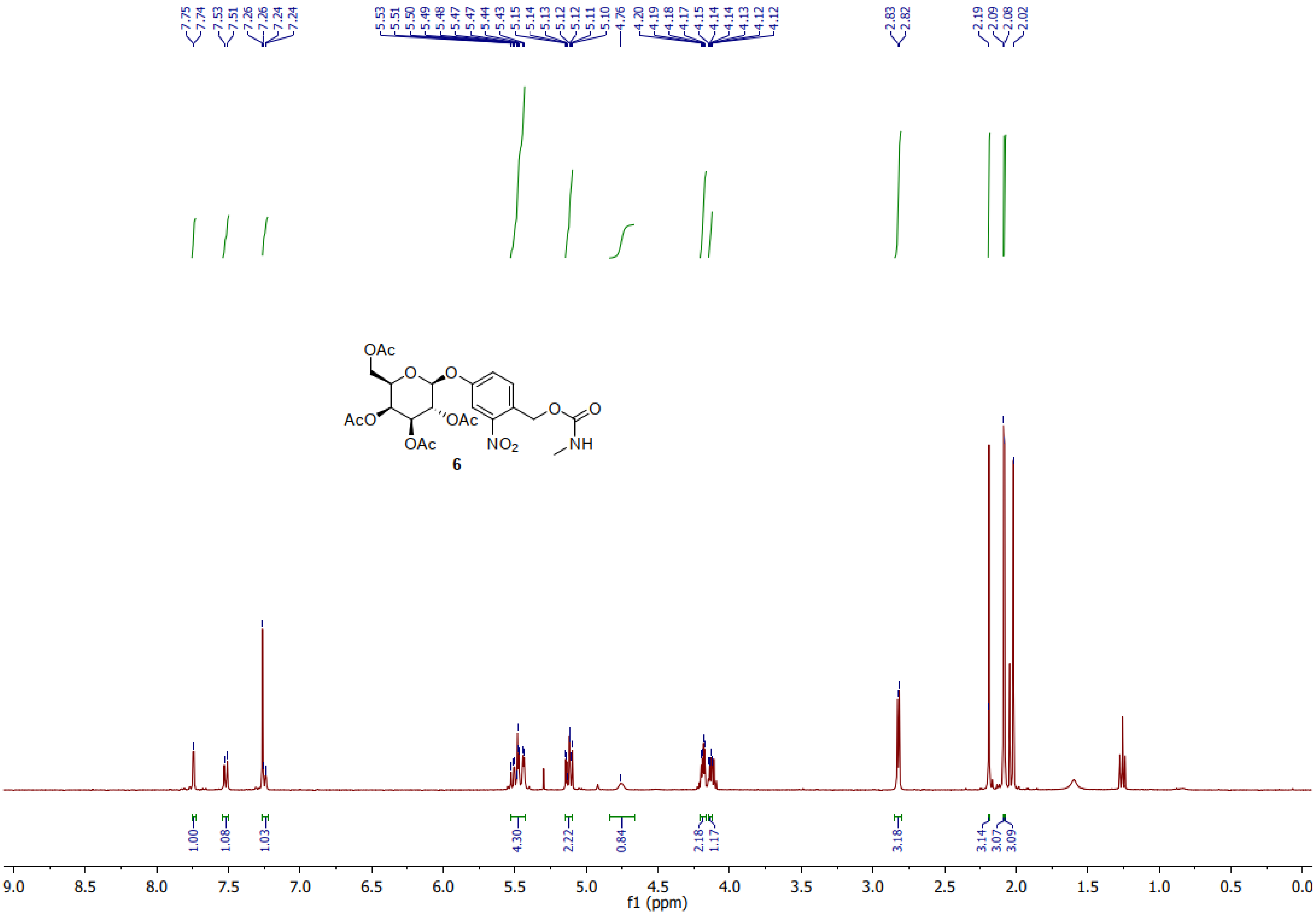

^{13}C NMR (101 MHz, $CDCl_3$) of compound **6**

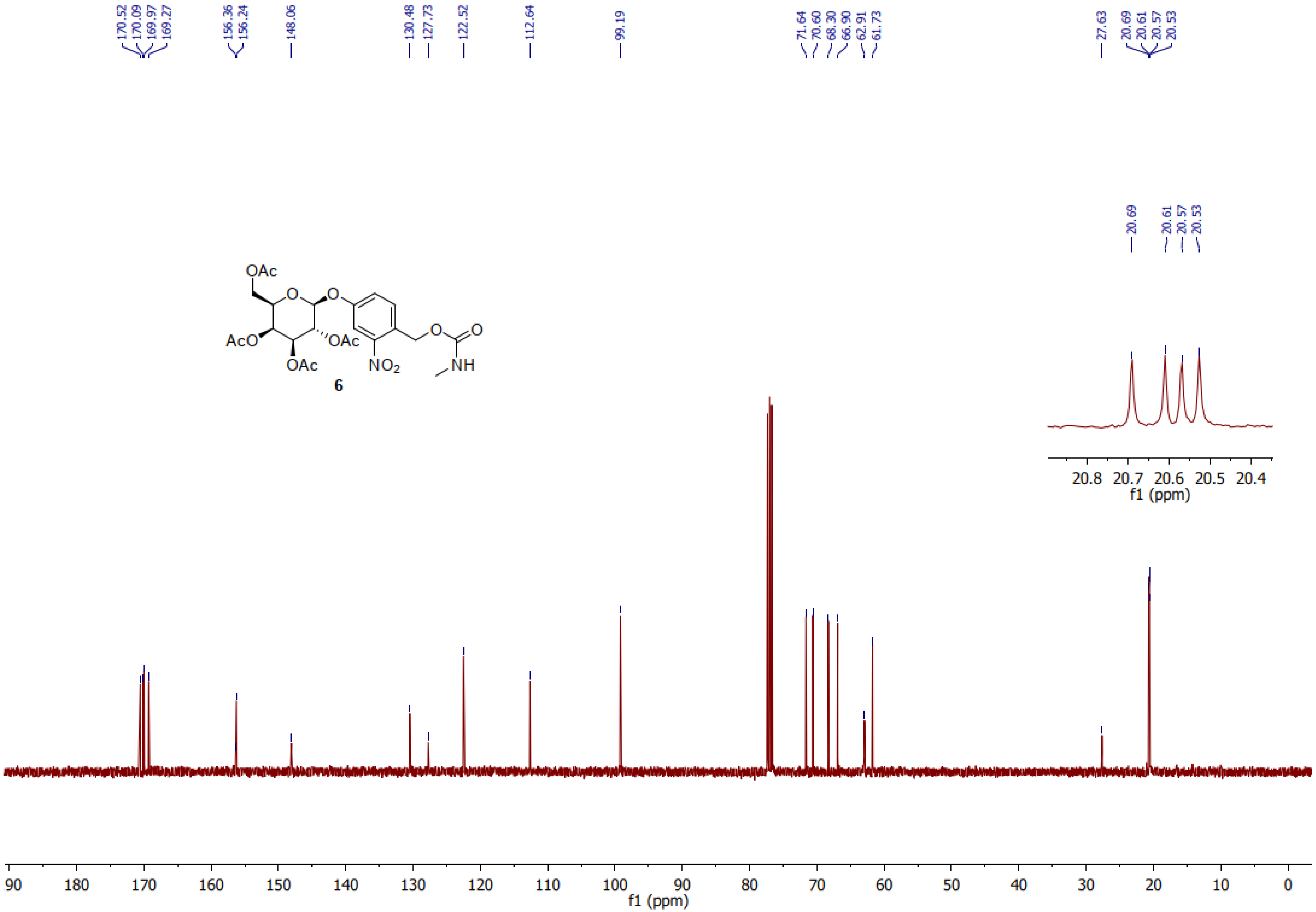

^{1}H NMR (400 MHz, $CDCl_3$) of compound **7**-Ac-^{t}BuO

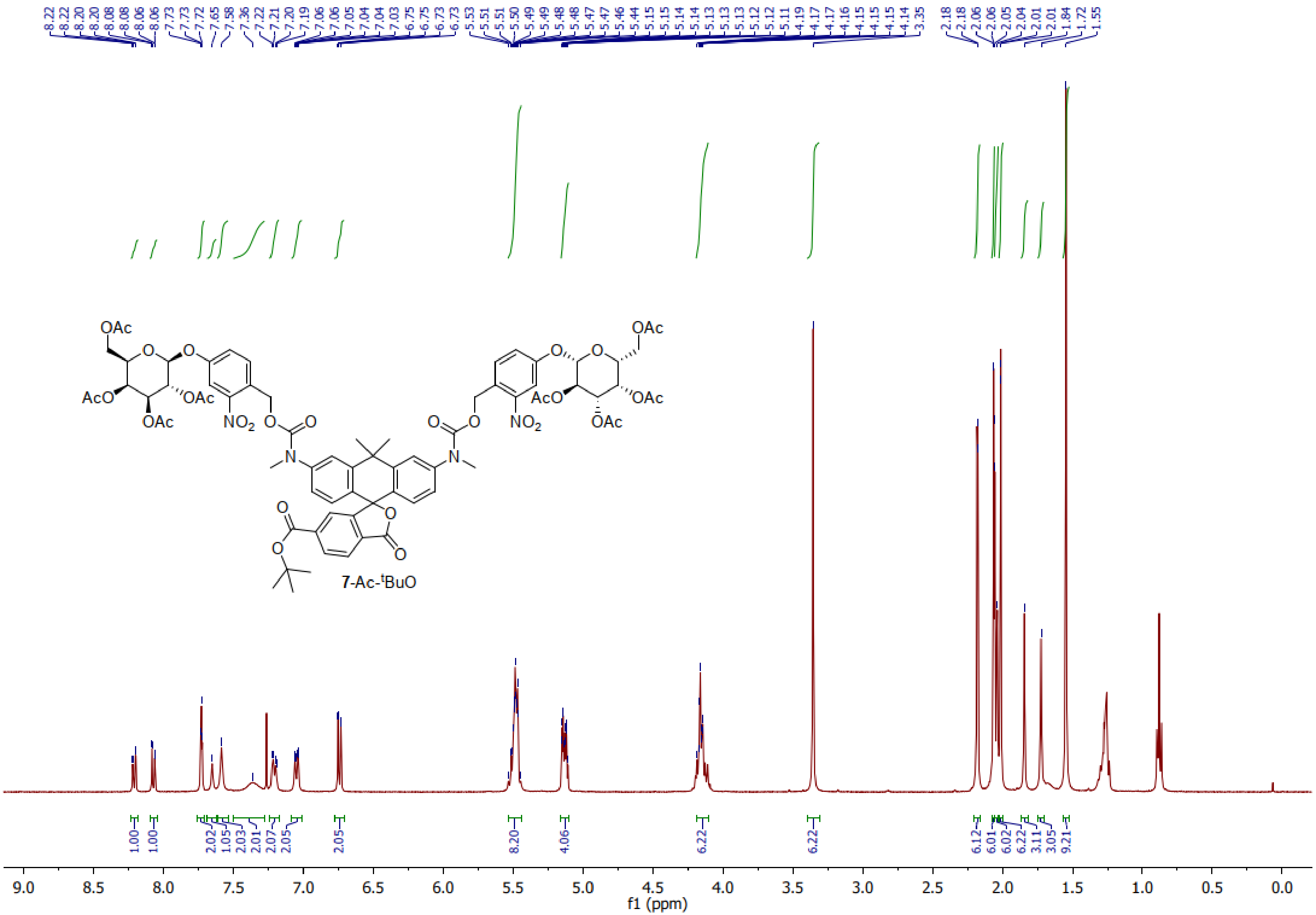

^{13}C NMR (101 MHz, CDCl$_3$) of **7**-Ac-^{t}BuO

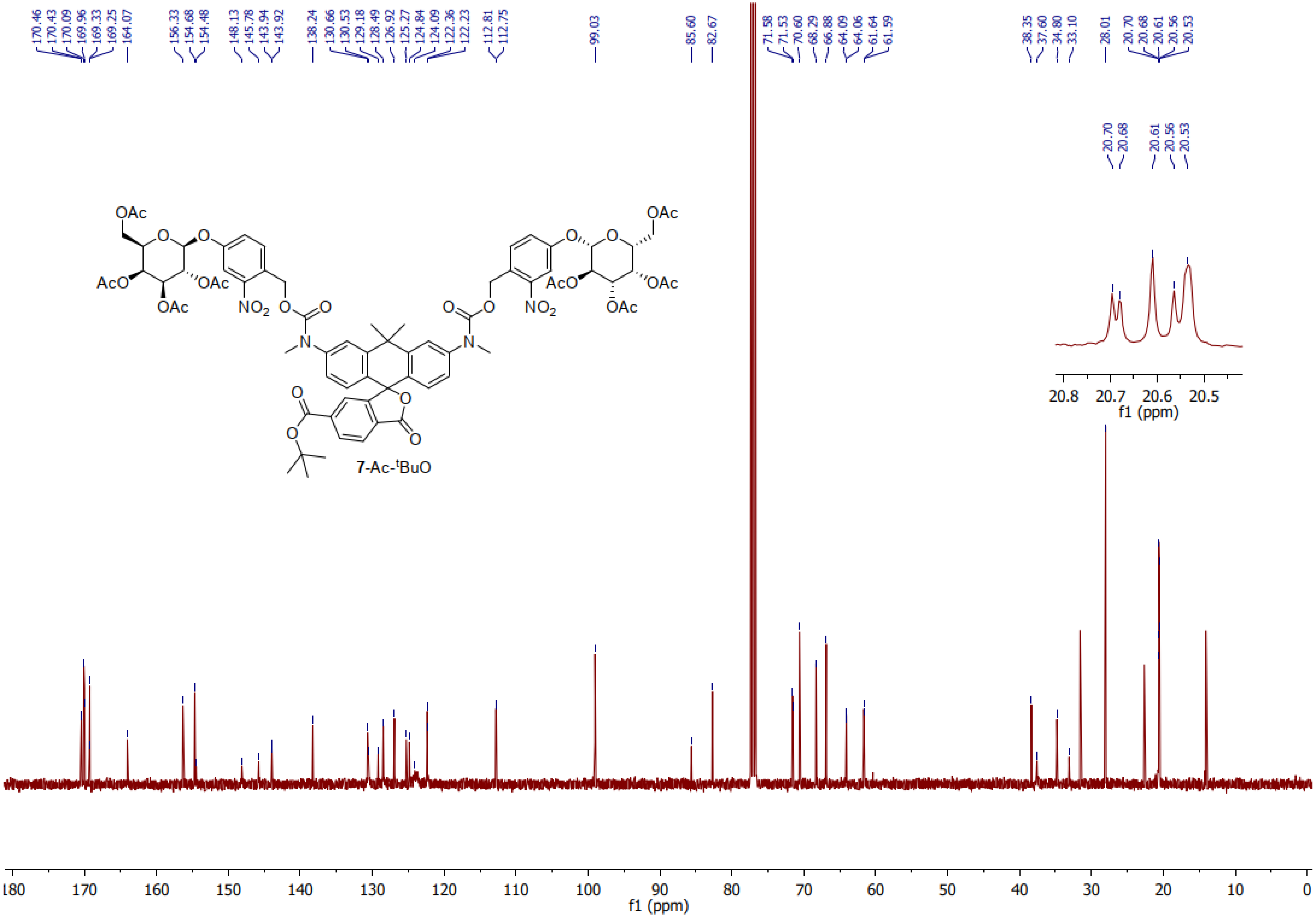

^{1}H NMR (400 MHz, CD_3CN) of compound **7**-Ac-OH

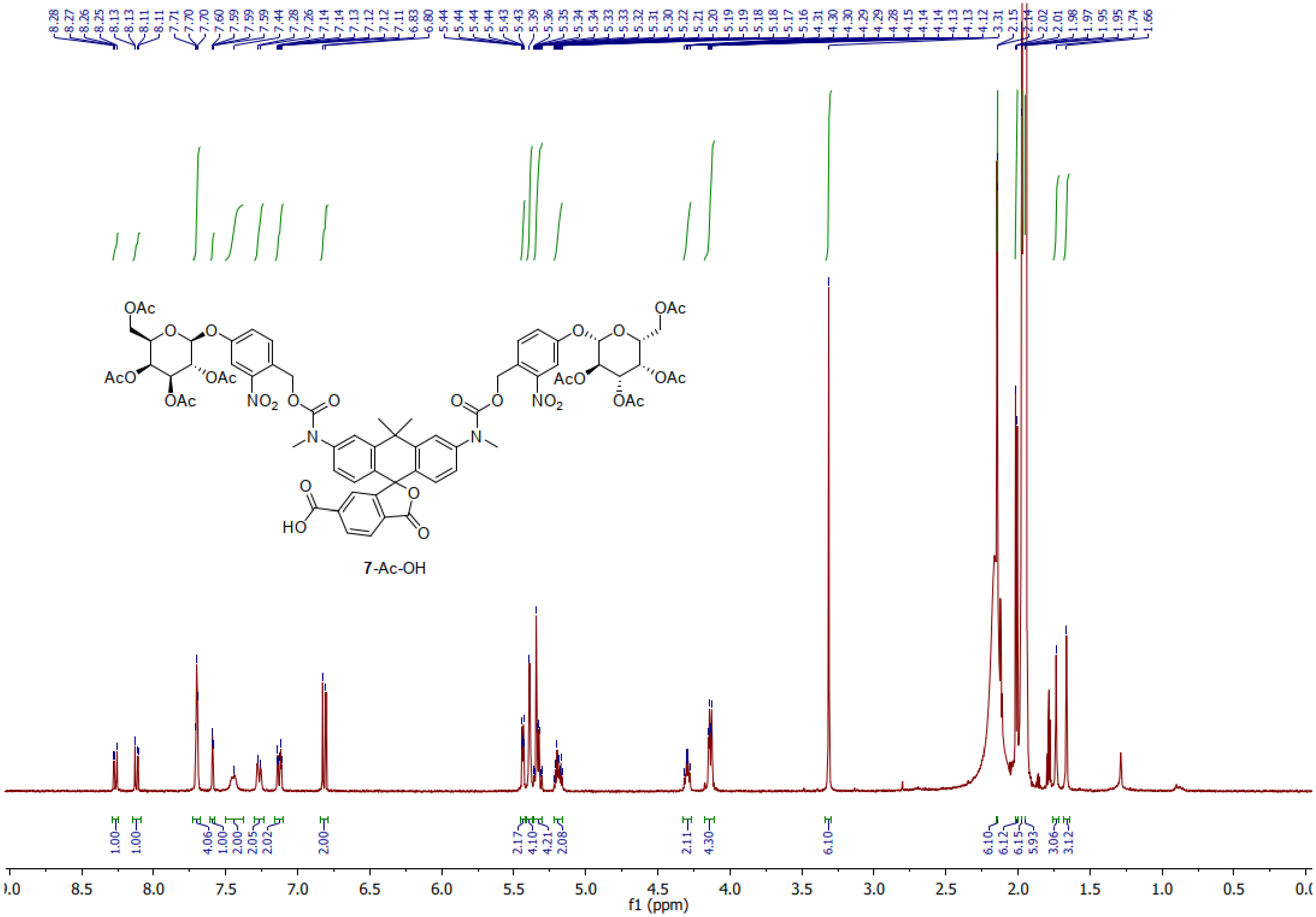

7-Ac-OH

^{13}C NMR (126 MHz, CD_3CN) of compound **7**-Ac-OH

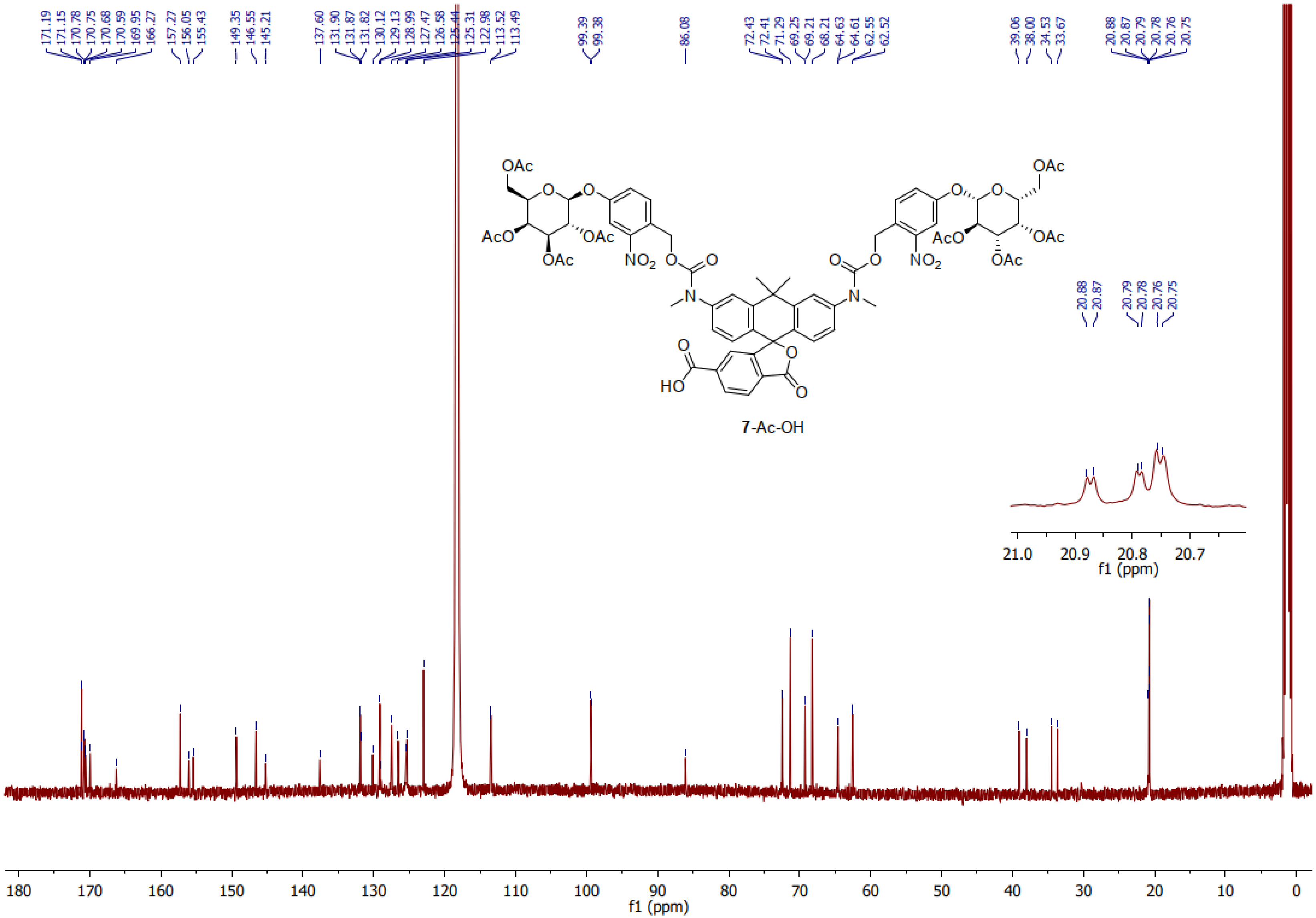

^{1}H NMR (400 MHz, CD_3OD) of compound **2**

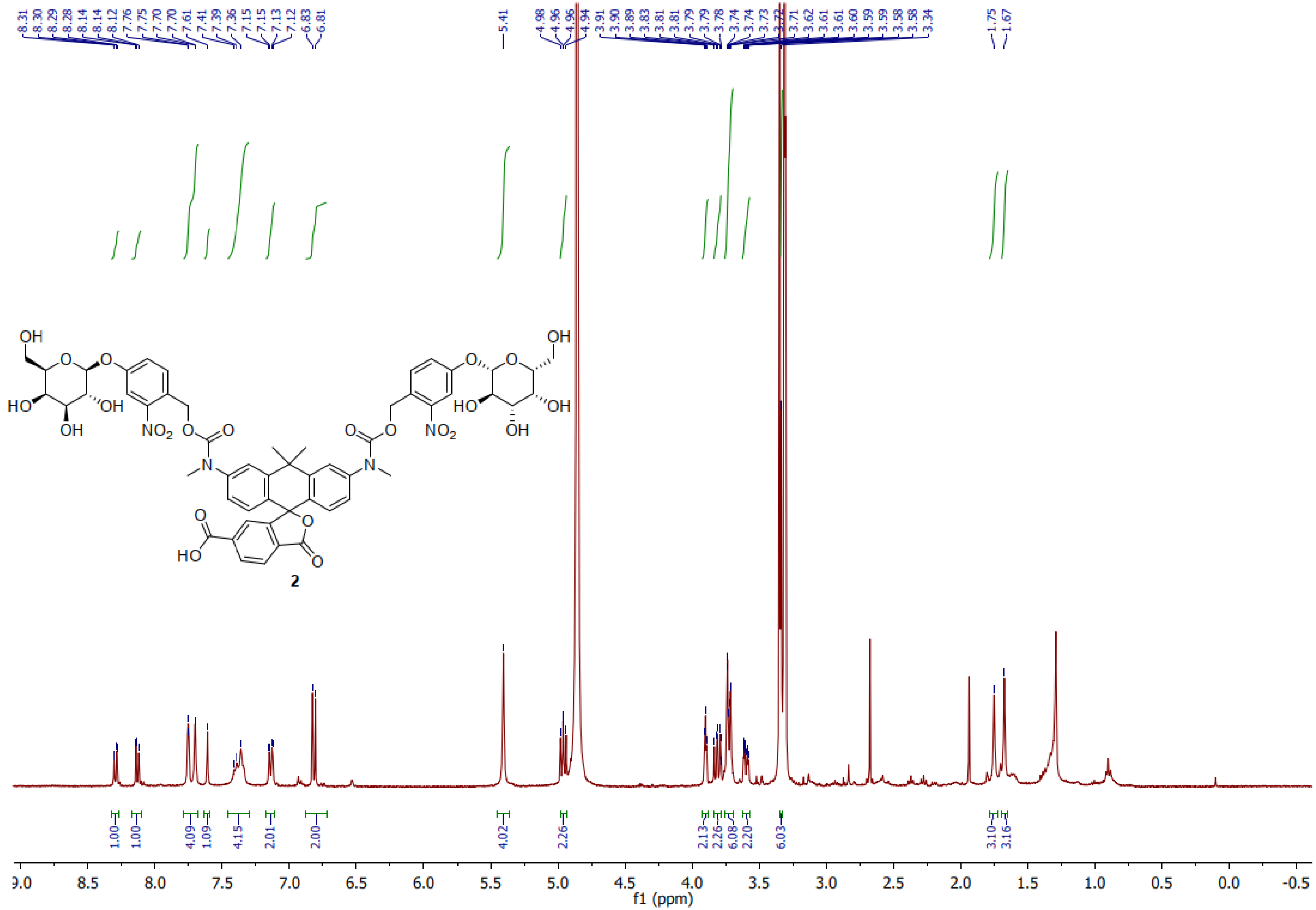

Analytical HPLC of compound **2**

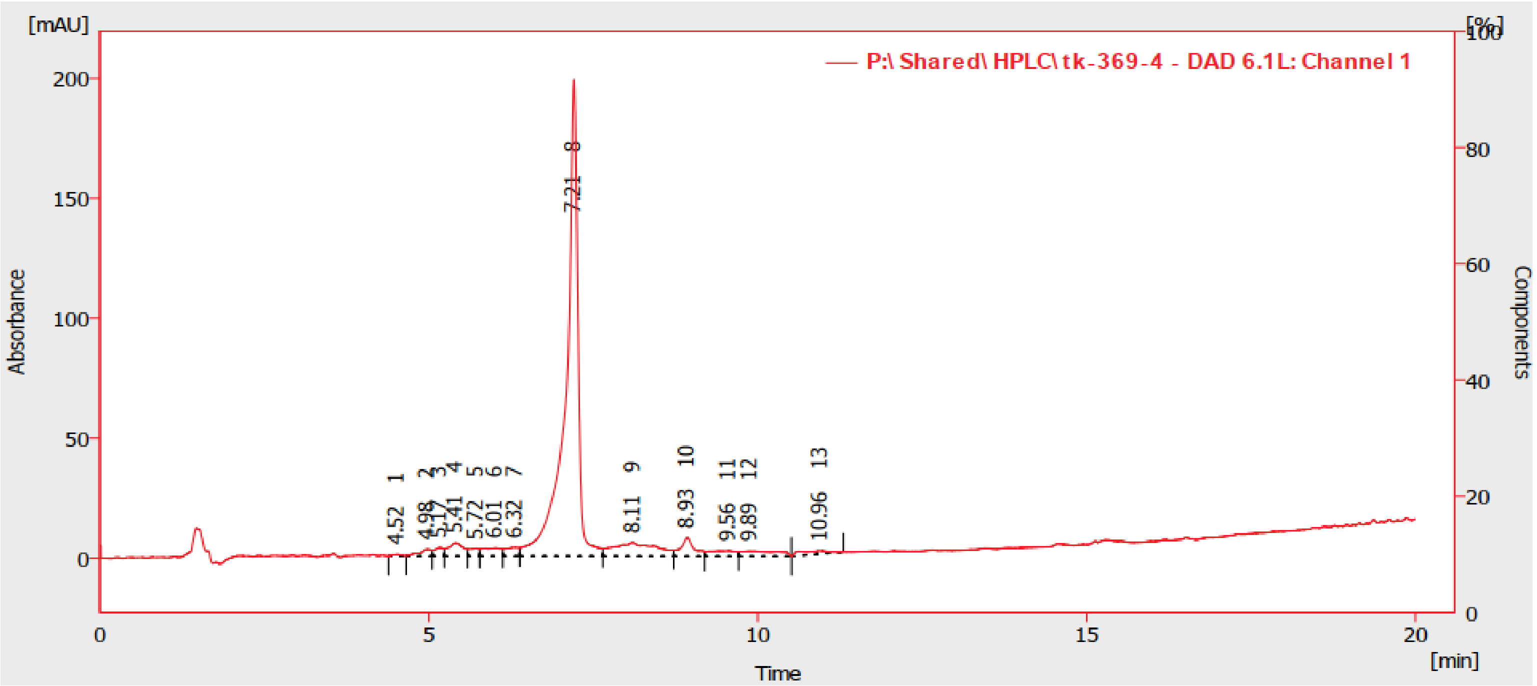

^1H NMR (400 MHz, CD_3CN) of compound **7**-Ac-HT

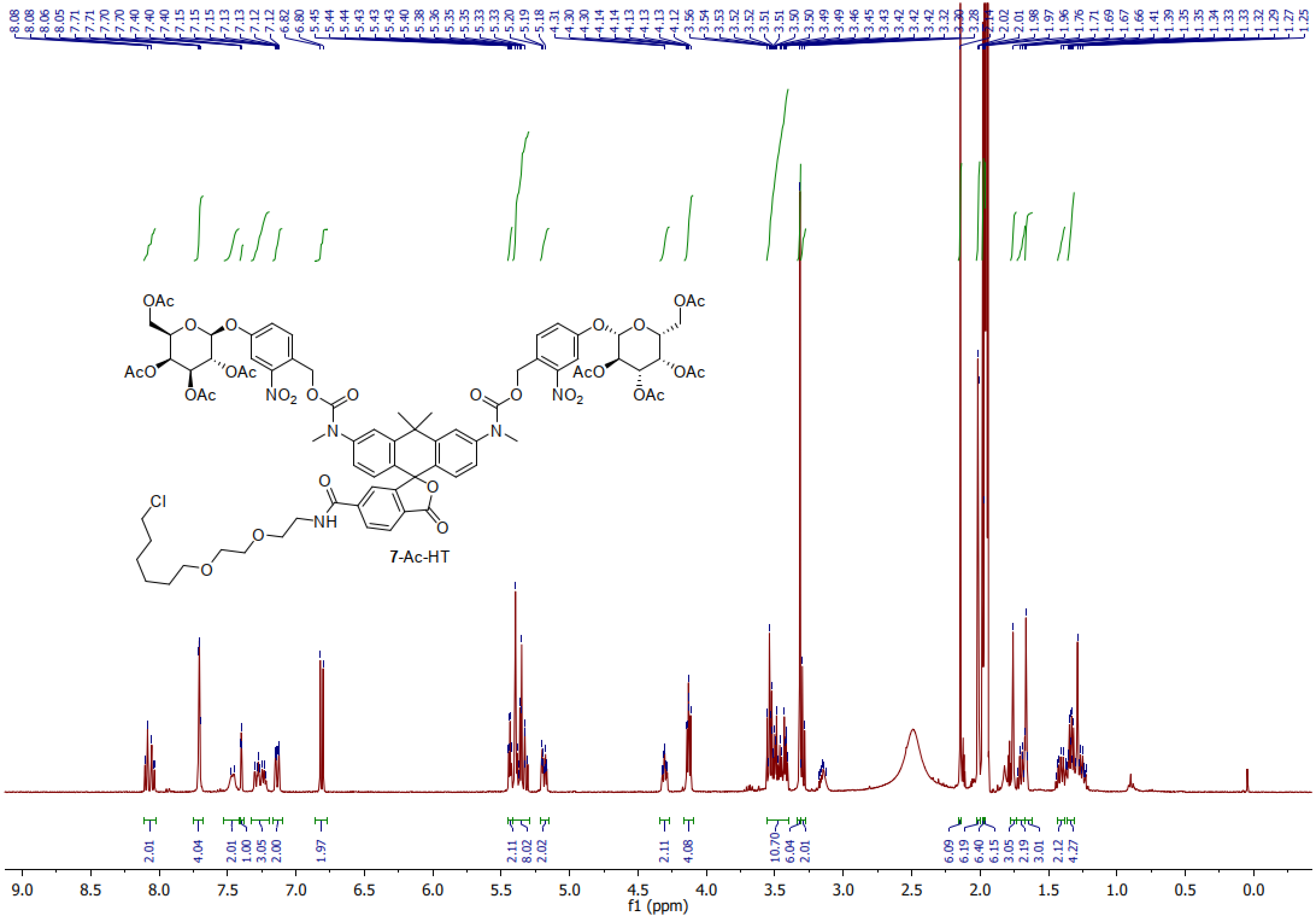

^{13}C NMR (126 MHz, CD_3CN) of compound **7**-Ac-HT

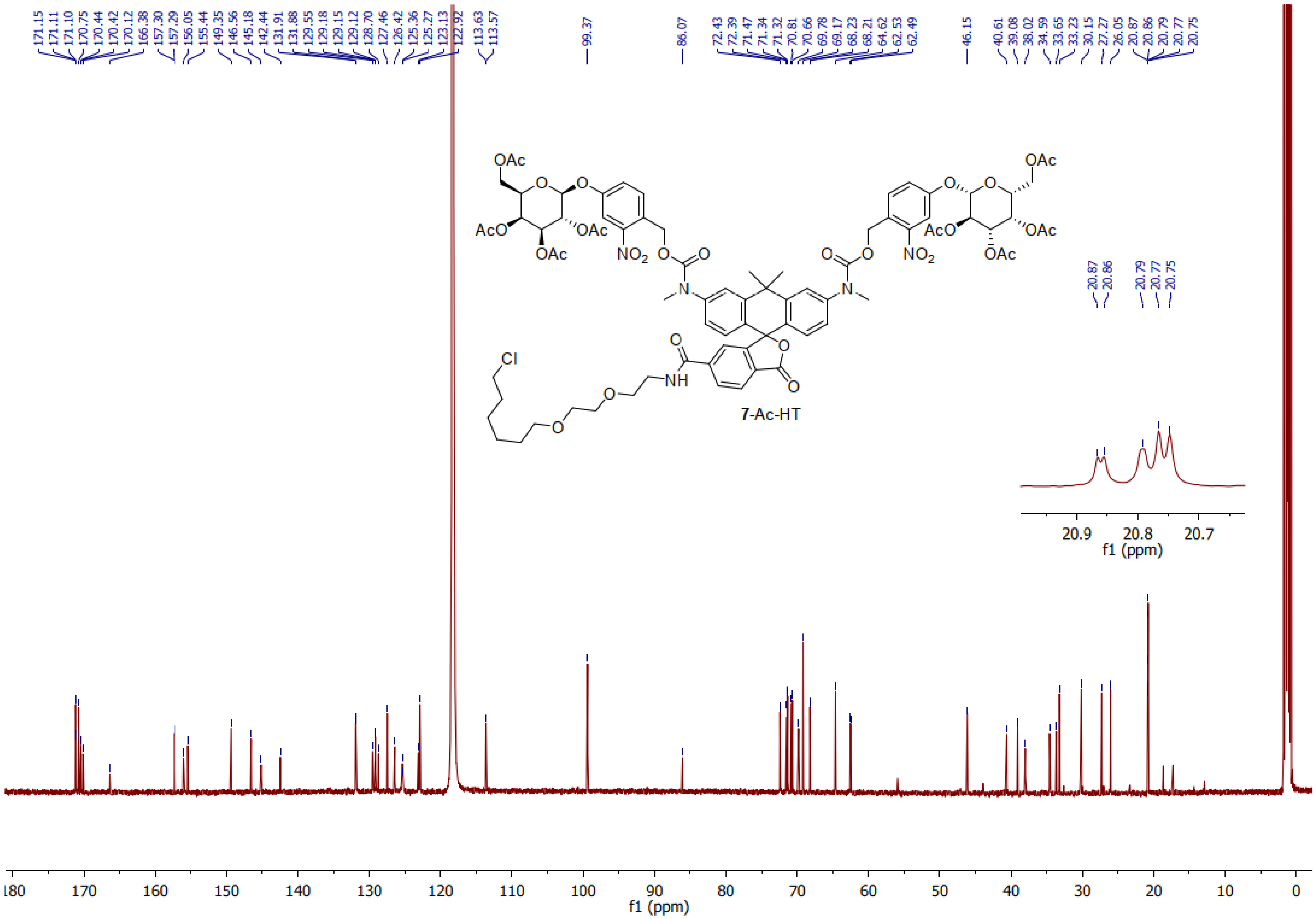

^{1}H NMR (400 MHz, DMSO-d_6) of compound **7**-H-HT

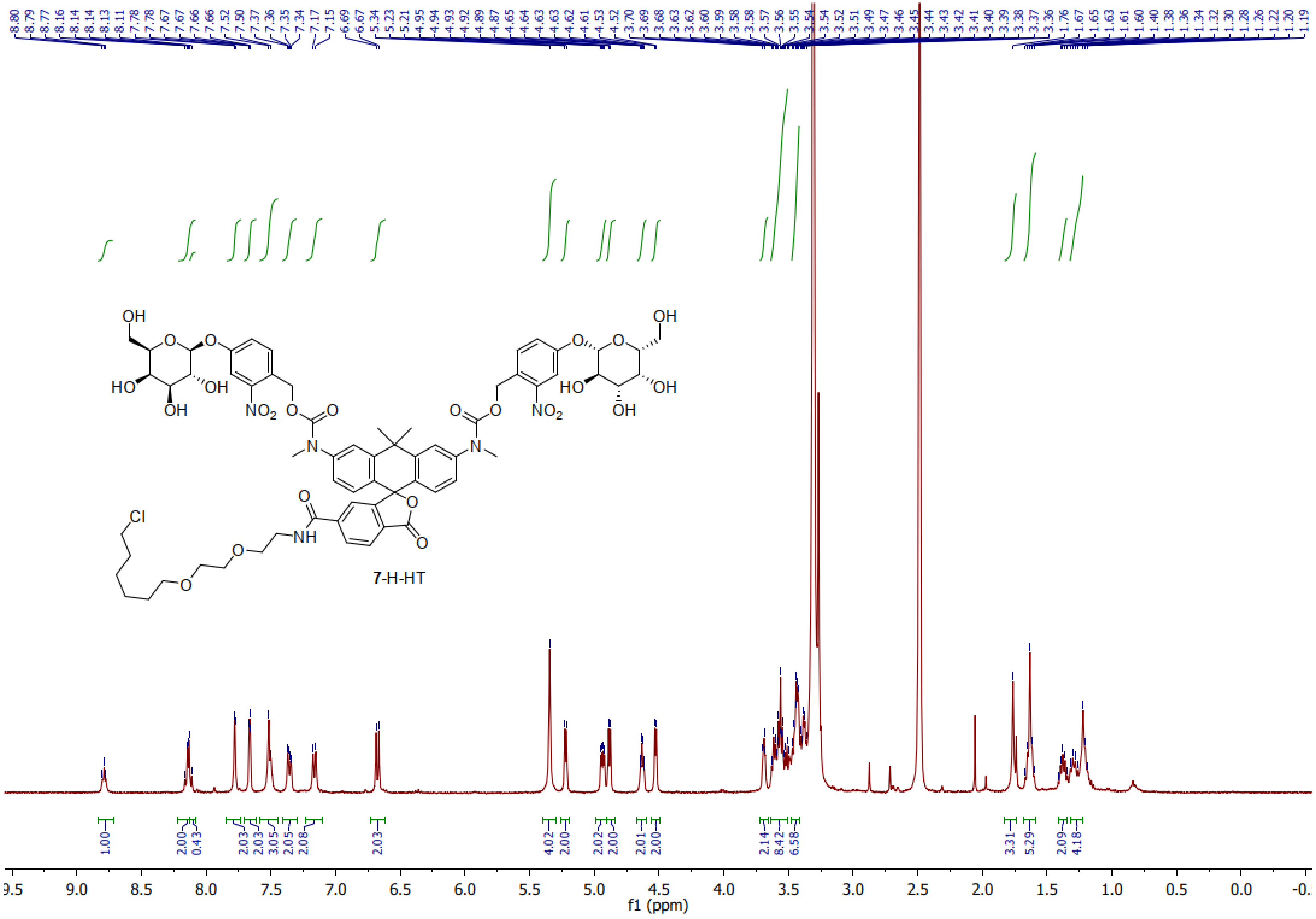

Analytical HPLC of compound **7**-H-HT

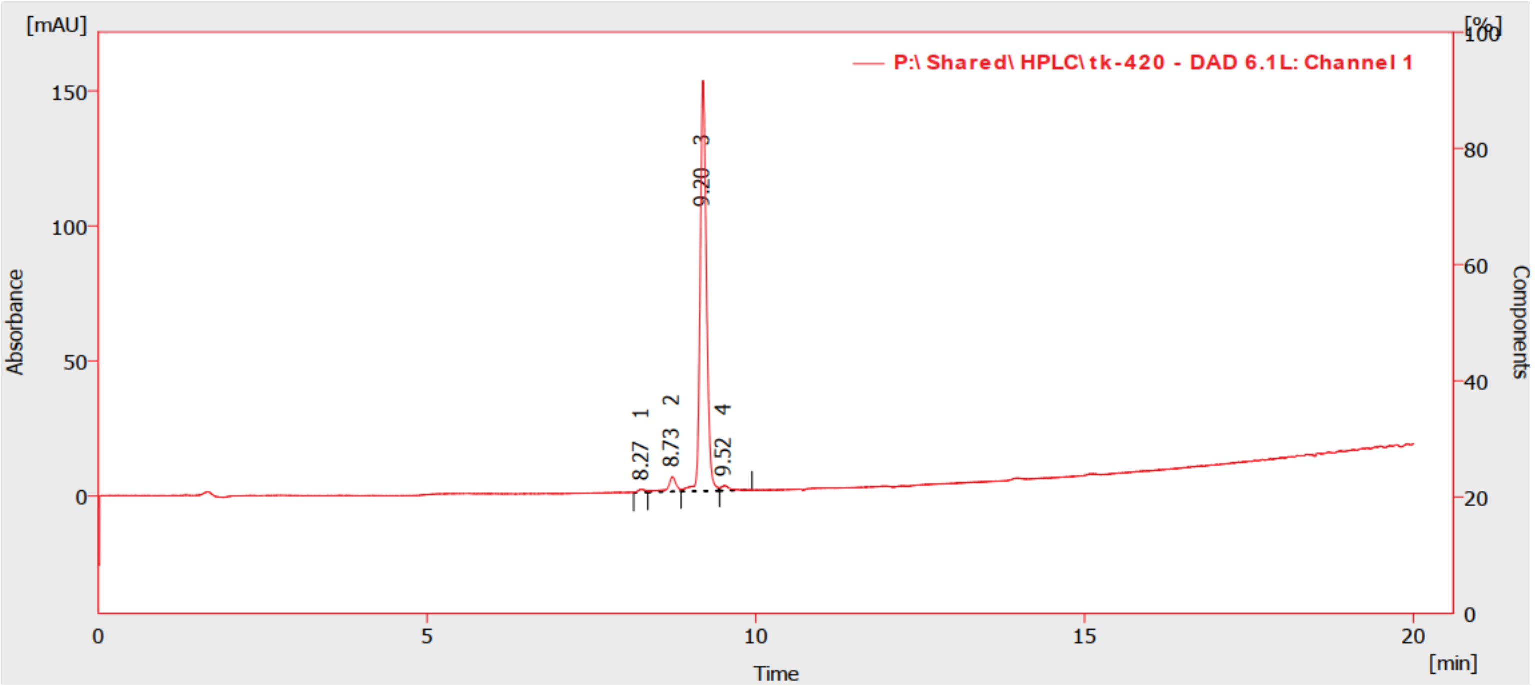

Supplement: Supplementary file 1 [file molecules-29-03596-s001.zip › molecules-3096062-supplementary.pdf]
